# Does Reproductive Success in Natural and Anthropogenic Populations of Generalist *Epipactis helleborine* Depend on Flower Morphology and Nectar Composition?

**DOI:** 10.3390/ijms24054276

**Published:** 2023-02-21

**Authors:** Emilia Brzosko, Andrzej Bajguz, Justyna Burzyńska, Magdalena Chmur

**Affiliations:** Department of Biology and Plant Ecology, Faculty of Biology, University of Bialystok, Ciolkowskiego 1J, 15-245 Bialystok, Poland

**Keywords:** broad-leaved helleborine, floral display, fruiting, nectar amino acids, nectar sugars, removal of pollinaria, road verges

## Abstract

The purpose of our study was to determine the role of flower structure and nectar composition in shaping the reproductive success (RS) of the generalist orchid Epipactis helleborine in natural and anthropogenic populations. We supposed that the distinct character of two groups of habitats creates different conditions for plant–pollinator relationships, thus influencing reproductive success in *E. helleborine* populations. Both pollinaria removal (PR) and fruiting (FRS) were differentiated between the populations. On average, FRS was almost two times higher in the anthropogenic than in the natural populations. The difference between the two population groups in PR was smaller but still statistically significant. RS parameters were correlated with some floral display and flower traits. Floral display influenced RS only in three anthropogenic populations. Flower traits had a weak influence on RS (10 of the 192 cases analyzed). The more important trait in shaping RS was nectar chemistry. The nectar of *E. helleborine* is relatively diluted with a lower sugar concentration in the anthropogenic than in the natural populations. In the natural populations, domination of sucrose over hexoses was found, while in the anthropogenic populations, hexoses were more abundant and the participation of sugars was balanced. In some populations, sugars influenced RS. In *E. helleborine* nectar, 20 proteogenic and 7 non-proteogenic amino acids (AAs) were found with a clear domination of glutamic acid. We noted relationships between some AAs and RS, but distinct AAs shaped RS in different populations, and their impact was independent of their participation. Our results indicate that the flower structure and nectar composition of *E. helleborine* reflect its generalistic character and meet the requirements of a wide range of pollinators. Simultaneously, the differentiation of flower traits suggests a variation in pollinator assemblages in particular populations. Knowledge about the factors influencing RS in distinct habitats helps to understand the evolutionary potential of species and to understand mechanisms and processes crucial for shaping interactions between plants and pollinators.

## 1. Introduction

Anthropogenic and climatic factors are the main causes of changes in the natural environment on different scales, from the population level to the geographical ranges of species. One of the plant groups especially sensitive to such alterations and simultaneously one of the most endangered plants are orchids [1,2]. Recently, more and more often, extinctions of orchid populations or even species due to anthropogenic driven disturbances have been documented in all climatic zones on all continents [1,2,3]. The threat concerns even the most valuable areas such as hotspots. The best examples are the depletion of many epiphytic orchids due to deforestation in tropical rainforests [4,5,6] or the disappearance of orchids from many biotopes in the Mediterranean hotspot due to changes in land use practices [7]. The main causes of orchid threat and rarity are habitat destruction and fragmentation, overcollection, climate change, and a variety of other human-induced issues [2,8,9,10,11,12]. Furthermore, the decline in many pollinators, including those crucial for the orchids [13,14,15,16], increases the risk of extinction of Orchidaceae representatives.

Simultaneously with the global decrease in orchid diversity, some species of this family show the ability to colonize secondary habitats: road verges, gravel pits, tree plantations, urban areas, cemeteries, and many others [8,17,18,19,20,21,22]. One of the orchid species that exists in different secondary habitats is an object of our study, *Epipactis helleborine*. It is a Eurasia species, which occurs in temperate and boreal zones. In Europe, it is distributed from Scandinavia and the British Isles to the Mediterranean and Urals, and in Asia, it grows in Asia Minor [23]. In North America, *E. helleborine* has a secondary geographical range. Kolanowska [24] suggests that the habitats available for this species will decrease due to climate change. *E. helleborine* is a long-lived, rhizomatous orchid. It has the ability of spontaneous autogamy, and pollinator behavior increases its self-pollination [22,25]. The shoots are straight, up to 100 cm high with few to up to 100 flowers. The shell-shaped hypochile and epichile knobs form a nectary, but the secreted material is significantly higher in hypochile cells [26]. The presence of more than 100 chemical compounds was found in *E. helleborine* nectar, and the main attractants are ethanol, eugenol, methoxyeugenol, and vanillin [27]. They are responsible for the characteristic behavior of visitors known as ‘drunken insects’ or ‘sluggish pollinators’ [28]. Mainly Vespidae and Apidae pollinate the flowers of *E. helleborine,* although representatives from Formicidae, Syrphidae, Coleoptera, and Lepidoptera may be pollinators [22,27,29]. *E. helleborine* grows mainly in neutral and alkaline, but sometimes in weakly acidic, soils and occupies a wide range of habitats [23] including secondary habitats [17,18].

The characteristics of anthropogenic habitats create different conditions for orchids compared to those observed in their natural environments. These differences include not only abiotic factors (e.g., soil parameters) but also biotic components, among which the most important are orchid partners (mycorrhizal fungi and pollinators) essential for their growth, development, and reproduction. Different soil resources in anthropogenic habitats influence the traits of orchid individuals such as size, flower production, nectar quantity, and quality [21,22,30,31,32]. The secondary character of the soil substrate in anthropogenic habitats may also change the composition and abundance of mycorrhizal fungi, on which the distribution and fitness of orchids depend [33,34]. In human-made habitats, different and often untypical plant communities have evolved, and their plant components determine the presence of specific insect assemblages (distinct from operating in natural habitats of orchids) required for pollination. Under such circumstances, flowers can adapt their properties according to pollinator types to achieve reproductive success, because pollination success depends on the mutual match between the structure of flowers and pollinators [35,36,37]. Numerous studies document the pollinator-mediated selection of flower traits in orchids [37,38,39,40,41,42,43,44,45,46]. The better mechanical fit between the flower and the pollinator increases the precision of pollen transfer, affecting the effectiveness of plant reproduction [35,47].

In nectariferous species, the distinct character of anthropogenic soils can also change the chemistry of the most effective reward, that is, the nectar [48,49,50,51]. Its quantity and quality should meet the pollinator’s requirements, according to the mouth apparatus and the dietary needs. The first articles on the topic, for example, Baker and Baker [52] and the results of further studies [53,54,55,56,57,58], show that nectar traits, especially sugar concentration and the ratios between them, are related to pollinator type. Plants produce nectar to attract pollinators, optimizing their composition according to the requirements of particular animals, and thus nectar composition may be an effect of pollinator-mediated selection [59]. Pollinators or their whole groups may show preferences for the nectar concentration, the number of sugars and amino acids (AAs), as well as particular nectar components. The most concentrated nectar was characterized in plants pollinated by bees, while the lowest was characterized in those pollinated by bats and hawkmoths [55,58,60,61]. In orchids, the concentration of sugars in nectar ranges from low to 90%, and nectar with the highest concentration was noted for bee-pollinated orchids [48]. The majority of plants produce nectar dominated by sucrose [54,62,63], but the domination of hexoses over sucrose is also noted [64,65,66]. Different pollinators often show distinct preferences for three main sugars (sucrose, glucose, and fructose). For example, bees and wasps select sucrose-dominated nectar [48,55], flies prefer hexoses over sucrose [50,52,53,54,66], and some birds and ants feed even on sucrose-free nectar [67]. Pollinator preferences may also concern the number and composition of AAs, and particular AAs may attract or discourage pollinators. Butterflies need a higher concentration of AAs in the nectar, while birds or flies choose nectar with a lower amount of these components [50].

Since orchid populations are usually small, the presence, diversity, and abundance of their pollinator assemblages depend on other plants useful to them, for example, by providing nectar. Plant communities rich in nectariferous species offer more nutritional resources, causing an increase in pollinator diversity and number, as well as the frequency of their visits, resulting in the increased reproductive success (RS) of particular species [68,69,70,71,72]. The diversity of insects is especially important for generalistic plants whose pollination depends on many pollinators. However, a higher diversity of plants may have the opposite effect on the effectiveness of pollination. When pollinators are not numerous and plant species share pollinators, the competition for them increases [69,70,73,74]. Pollinator deficiency is the main cause of low fruiting in orchids [75] and is often observed in anthropogenic populations [16]. It should also be emphasized that under new circumstances in secondary habitats, new relations between symbiotic partners may arise. This requires an adaptation of flowers to pollinators operating in changed habitats. As a result, the level of reproductive success in anthropogenic populations may differ from that observed in natural populations. Comparisons of the levels and factors that influence RS in anthropogenic and natural populations determine the amount of evolutionary potential of species and explain the mechanisms and processes crucial to shaping interactions between plants and pollinators.

Our research fits into the above-mentioned problems. The main objective of our study was to evaluate the level of female reproductive success (FRS, fruiting) and male reproductive success (PR, pollinaria removal) and to determine the role of flower morphology and nectar composition in shaping RS in natural and anthropogenic populations of the generalist orchid *E. helleborine*. We assumed that a high level of RS would be due to the following traits of this species, which increase the chance of effective pollination: (a) as a generalist, it is pollinated by a wide range of pollinators; (b) self-compatibility enables autogamy and geitonogamy; and (c) nectar production. We hypothesized the variation in RS and differentiated the role of particular flower traits in shaping RS for particular populations, where the greatest differences were expected between natural and anthropogenic ones. The answer to the question of how variation in flower traits is distributed in populations in distinct habitats and in which way they shape reproductive success is important to explain the evolutionary processes that cause changes in plant–pollinator interactions.

## 2. Results

### 2.1. Floral Display and Flower Structure

We found a wide range of values in floral display traits and statistically significant differences between *E. helleborine* populations (shoot high: F = 7.961, *p* = 0.0; inflorescence length: F = 2.502, *p* = 0.017; the number of flowers: F = 8.043, *p* = 0.0). The lowest shoots were in aBIA (50.8 ± 2.7 cm), while the highest were in the nZAB population (75.1 ± 1.9 cm) (Table 1). The shortest inflorescences were observed in aBIA (15.7 ± 1.4 cm) and nPOG2 (15.8 ± 0.8 cm), and the longest in nZAB (20.4 ± 0.7 cm) and nPOG1 (20.4 ± 1.3 cm). The lowest number of flowers developed in nPOG2 (7 ± 0.3), and the largest in aSUR (9.3 ± 0.2). Generally, the shoots in the natural populations were approximately 8 cm higher but developed six fewer flowers than in the anthropogenic populations (F = 14.844, *p* = 0.0002 and F = 18.988, *p* = 0.00003, respectively).

The values of the flower traits, excluding LE, differed significantly between populations (F = 2.53–8.86, *p* < 0.05). Some flower structures (EL, HL) were larger in the anthropogenic than in the natural populations, while the remaining were similar in both groups (Table 1).

### 2.2. Sugars in Nectar

**Figure 1:**
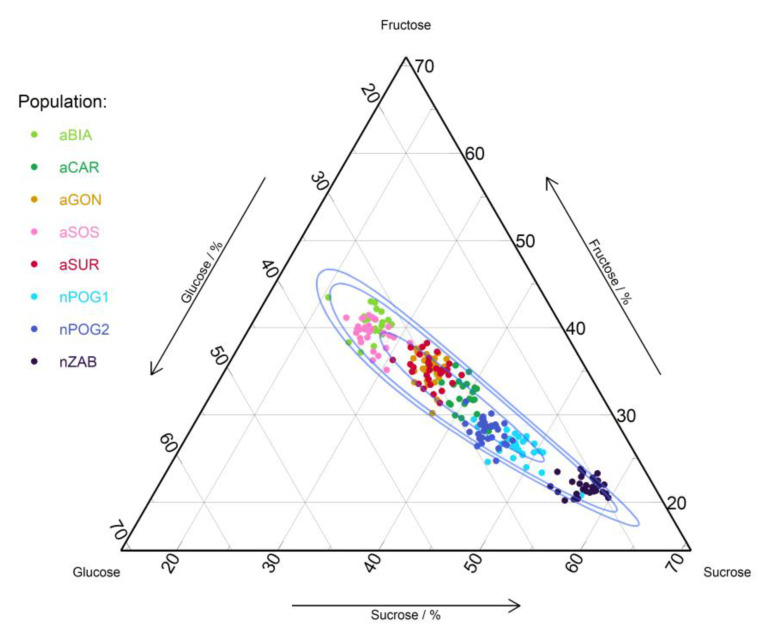
Ternary plot of sugars for anthropogenic (aBIA, aCAR, aGON, aSOS, and aSUR) and natural (nPOG1, nPOG2, and nZAB) populations of *Epipactis helleborine*. The blue lines show the 50%, 90%, and 95% confidence intervals using the Mahalanobis distance and the log-ratio transformation.

*E. helleborine* nectar is relatively diluted. The percentage share of glucose ranged between 19.33 and 36.52%; sucrose between 20.55 and 59.76%; and fructose between 20.24 and 43.48%. The anthropogenic populations differed from the natural populations mainly in sucrose share, that is, the anthropogenic ranged from 20.55 to 44.11% while the natural ranged from 40.13 to 59.76% (Figure 1).

**Figure 2:**
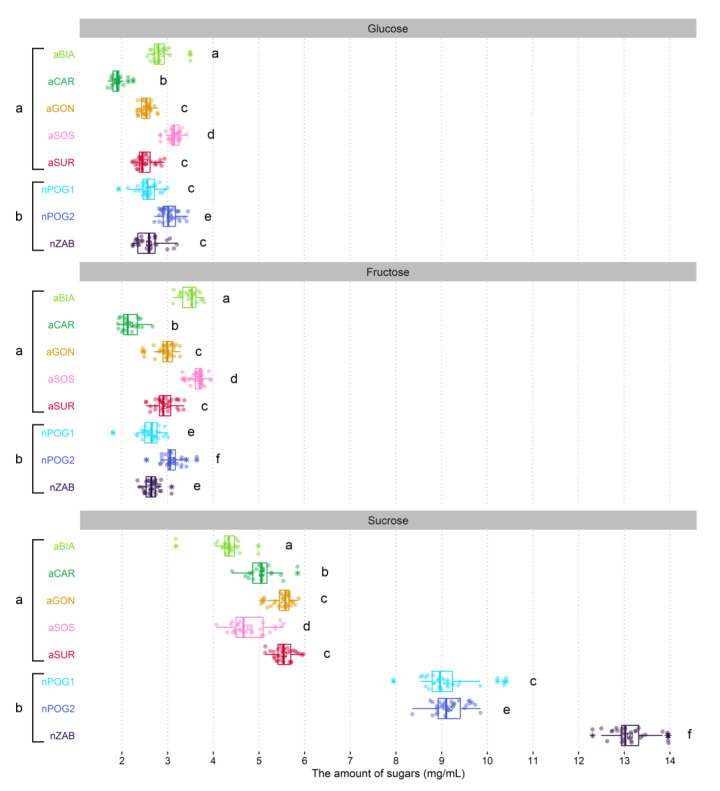
Boxplots of sugar amount for anthropogenic (aBIA, aCAR, aGON, aSOS, and aSUR) and natural (nPOG1, nPOG2, and nZAB) populations of *Epipactis helleborine*. The colored dots are individual samples. The square crossed shows the mean. The lower and upper hinges correspond to the lower (Q_1_) and upper (Q_3_) quartiles. Thus, the length of the box shows the interquartile range (IQR). The thicker line inside the boxes corresponds to the median. The lower whisker extends from the hinge to the smallest value at most Q_1_ − 1.5 × IQR of the hinge. The upper whisker extends from the hinge to the highest value no further than Q_3_ + 1.5 × IQR. Data beyond the end of the whiskers, indicated with an asterisk symbol, are outliers. Different lowercase letters indicate statistically significant differences according to the pairwise Wilcoxon rank sum test with the Benjamini–Hochberg adjustment (*p* < 0.05). Additional comparisons between natural and anthropogenic populations on the left/right sides are shown.

We noted statistically significant differences in the sugar concentration between all populations (F = 40.301, *p* = 0.0) and between groups of the natural and anthropogenic populations (F = 95.082, *p* = 0.0) (Table 2). We also found statistically significant differences between the populations in sugar quantity (F = 946.041; *p* = 0.0) with a twofold difference between the lowest and highest value of this parameter. The total amount of sugars was significantly lower in the anthropogenic (9.14 ± 0.48 − 11.64 ± 0.41 mg/mL) than in the natural populations (14.29 ± 0.69 − 18.34 ± 0.69 mg/mL; Figure 2). The amount of three sugars (that is, fructose, glucose, and sucrose) also differed significantly between the populations (F = 394.200–858.965, *p* = 0.000). In natural populations, sucrose domination was found over hexoses (S:H ratio equaled 1.5–2.5), while in two anthropogenic populations (aSOS and aBIA), hexoses were more abundant (S:H = 0.7), and in the remaining three anthropogenic populations (aCAR, aGON, and aSUR), the participation of these sugars was more balanced and equaled 1 and 1.2, respectively (Table 3). Statistically significant differences were also observed between natural and anthropogenic populations in the fructose:glucose ratio. In natural populations, this ratio was close to 1, while in anthropogenic populations, the participation of glucose was higher than that of fructose (Table 3).

### 2.3. Amino Acids in Nectar

The populations of *E. helleborine* differ according to the concentration of AAs (F = 4768.729, *p* = 0.0; Table 4). The total concentration of AAs in the nectar ranged from 370.0 ± 9.6 μM in nZAB to 768.3 ± 11.6 μM in aSUR. At the species level, we found 27 different AAs (20 proteogenic and 7 non-proteogenic) with different numbers and participation in particular populations (Figure S1, Table 4). All 27 AAs were found only in aSOS, while 26 AAs were present in the 6 populations, and the lowest number of AAs (25) was detected in nPOG1. In all natural populations, GABA was absent, and in nPOG1, the lack of β-Ala was reported. On the other hand, in the anthropogenic populations, Cit (aCAR, aGON, and aSUR) and Met (aBIA) were absent. In all populations, the dominance of Glu (26.7–37.7%) and the important participation of Asp (8.1–17.8%) were found. The most frequent were AAs with a low percentage (max. ca. 9% in some populations, as in the case of Asn, Gln, and Thr). The participation of the remaining nine AAs, i.e., Tyr, Arg, Met, Cit, Tau, AABA, BABA, GABA, and β-Ala, was generally below 1%. Some AAs, i.e., Asp, Gln, Gly, Pro, and β-Ala, had significantly higher participation in the anthropogenic than in the natural populations (Table 4).

**Figure 3:**
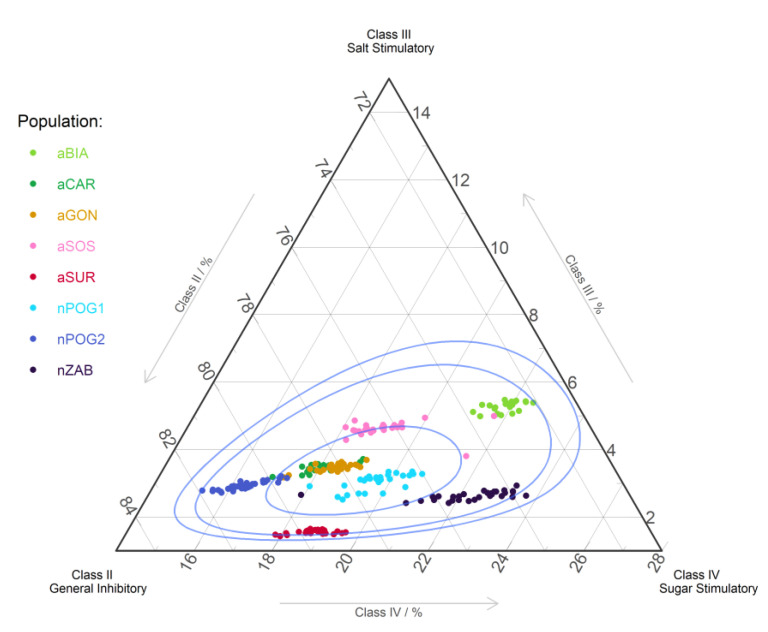
Ternary plot of amino acid classes for anthropogenic (aBIA, aCAR, aGON, aSOS, and aSUR) and natural (nPOG1, nPOG2, and nZAB) populations of *Epipactis helleborine*: II (Asp, Glu, His, Arg, and Lys), III (Pro), and IV (Val, Met, Trp, Phe, Ile, and Leu). The blue lines show the 50%, 90%, and 95% confidence intervals using the Mahalanobis distance and the log-ratio transformation. The first class of AAs (Asn, Gln, Ala, Cys, Gly, Ser, Thr, and Tyr) does not affect the chemoreceptors of the fly (data not shown).

The amino acids responsible for the taste of nectar were divided into four classes. We found notable differences between the natural and anthropogenic populations. The possible simulation of insect chemoreceptors by AAs in the nectar has AAs from classes II (chemoreceptor inhibitors), III (stimulation of salt cells), and IV (stimulation of sugar cells). The percentage share of class II ranged between 35.8 and 56.0%, while that of class III ranged between 1.9 and 21.3% and that of class IV ranged between 35.9 and 47.9% (Figure 3). Fifty percent of the samples had a percentage share in the approx. range of 74–81% for class II, 2–4.5% for class III, and 17–22% for class IV. The aGON and aCAR have similar percentage shares for all classes. The natural populations differ from anthropogenic populations mainly by the percentage share of class II AAs, that is, their percentage share ranged from approx. 2.5 to 3.5%, while the anthropogenic populations had a lower percentage share for aSUR and a higher percentage share for aBIA, aCAR, aGON, and aSOS.

**Figure 4:**
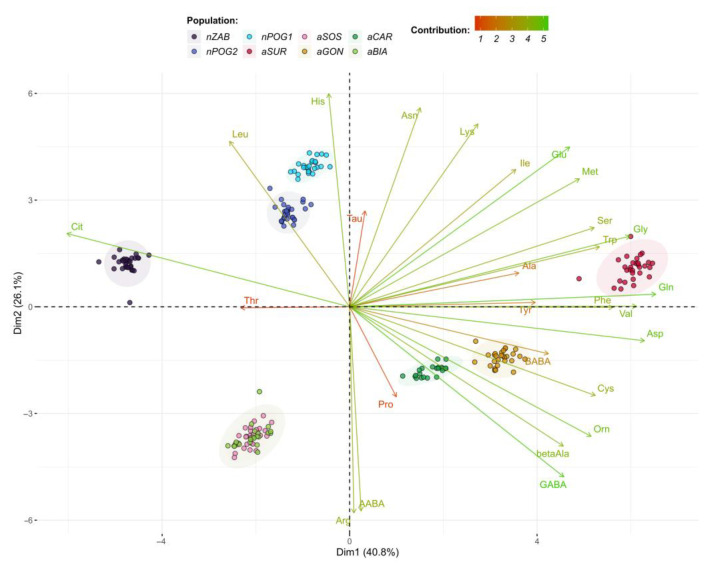
Biplot of amino acid profiles for the anthropogenic (aBIA, aCAR, aGON, aSOS, and aSUR) and natural (nPOG1, nPOG2, and nZAB) *Epipactis helleborine* populations showing the first two dimensions/factors (Dim1–2) of the PCA that together explain 66.9% of the variance. The biplot vectors indicate the strength and direction of factor loading for the first two factors. Individuals (populations) are color-coded by population. The ellipses around the individuals show an assumed 95% multivariate normal distribution.

**Figure 5:**
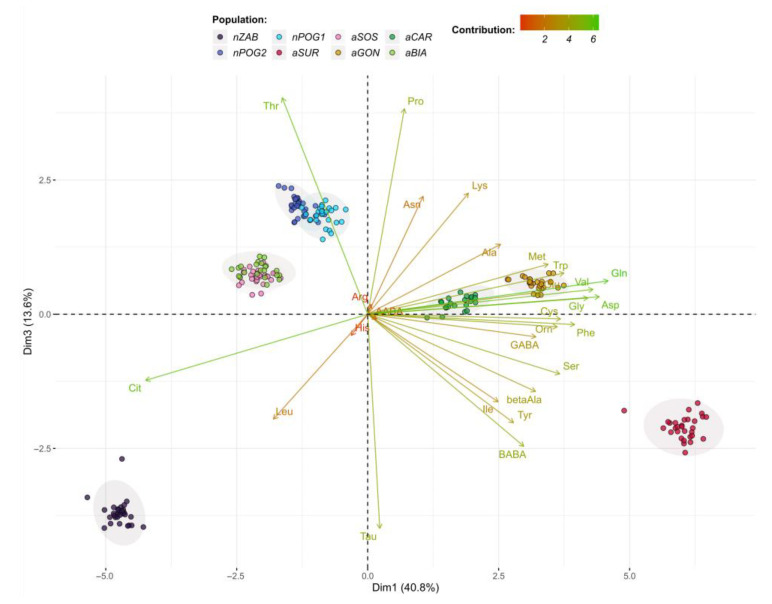
Biplot of amino acid profiles for the anthropogenic (aBIA, aCAR, aGON, aSOS, and aSUR) and natural (nPOG1, nPOG2, and nZAB) *Epipactis helleborine* populations showing the first two dimensions/factors (Dim1–3) of the PCA that together explain 54.4% of the variance. The biplot vectors indicate the strength and direction of factor loading for the first two factors. Individuals (populations) are color-coded by population. The ellipses around the individuals show an assumed 95% multivariate normal distribution.

**Figure 6:**
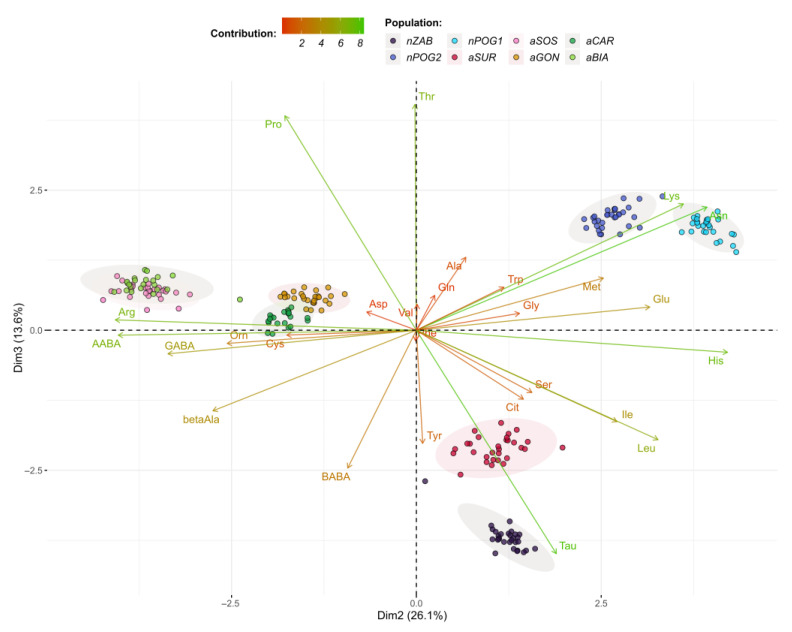
Biplot of amino acid profiles for the anthropogenic (aBIA, aCAR, aGON, aSOS, and aSUR) and natural (nPOG1, nPOG2, and nZAB) populations of *Epipactis helleborine*, showing the first two dimensions/factors (Dim2–3) of the PCA that together explain 39.7% of the variance. The biplot vectors indicate the strength and direction of factor loading for the first two factors. Individuals (populations) are color-coded by population. The ellipses around the individuals show an assumed 95% multivariate normal distribution.

The presented PCA model explains 80.5% of the variance using the first three principal components (Dim1–Dim3) (Figure 4, Figure 5 and Figure 6). According to the cos^2^ values obtained using the PCA model (Figure S3), Dim1 explains mainly the variations in Gln, Asp, Val, Cit, Gly, Phe, Trp, Cys, Ser, Orn, and Met variations; Dim2 explains the variations in His, Arg, AABA, Asn, and Lys; and Dim3 explains the variations in Thr, Tau, and Pro. The PCA grouped the six populations into pairs: nPOG1 and nPOG2, aSOS and aBIA, and aCAR and aGON. Positive scores for Dim1 indicate values of, for example, Asp, Val, Phe, Gln, Trp, Gly, and Ser (thus higher for aSUR, aGON, and aCAR) that are higher than the mean values for all studied populations, while negative scores correspond to higher values of Cit and Thr (thus higher for nZAB, nPOG1, nPOG2, aSOS, and aBIA). Positive scores for Dim2 indicate values of, for example, His, Asn, and Lys (thus higher for aSUR, nZAB, nPOG1, and nPOG2), while negative scores correspond to higher values of Arg and AABA (thus higher for aSOS, aBIA, aCAR and aGON). Positive scores for Dim3 indicate values of, e.g., Thr and Pro (which are higher for nPOG1, nPOG2, aSOS, and aBIA), while negative scores correspond to the higher values of Tau (which are higher for nZAB and aSUR). Other differences between the populations are also visible, e.g., nPOG1 and nPOG2 differ from aGON and aCAR mainly by the higher contents of Leu, His, and Cit as the well as lower contents of, e.g., AABA, β-Ala, Orn, Cys, Asp, Val, Phe, and Gln.

### 2.4. Reproductive Success

The reproductive success in populations of *E. helleborine* was shaped at different levels (Table 5). The PR ranged from 56.4 ± 42.5% in nPOG2 to 90.1 ± 26.1% in nZAB. Even greater differences were observed in fruiting—the lowest (23.8 ± 37.6%) in nPOG1 and the highest (above 90%) in aSOS and aBIA. The populations differed in pollination efficiency. The highest PR:FRS ratios were observed in the nPOG1 and nPOG2 populations (32.4 ± 42.3 and 37.4 ± 42.9, respectively), and the lowest in aSOS and aBIA (ca. 0.9 ± 0.2). In four populations, pollinators did not visit some inflorescences (in aCAR—14.3%, aGON—13.8%, nPOG1—7.4%, and nPOG2—27.6%, both pollinaria were not removed and fruits were not developed). Excluding the aSOS and aBIA populations, fruitless shoots were observed, with the highest participation in nPOG1 and nPOG2 (37% and 62.1%, respectively). From some shoots, pollinaria were removed but fruits did not develop (e.g., one-third of such shoots were observed in nPOG1 and nPOG2). However, in single shoots of aCAR and aGON, fruits were observed despite pollinaria not being removed. On average, FRS was almost two times higher in the anthropogenic than in the natural populations (F = 55.461, *p* = 0.0). The difference between two population groups in PR was smaller but still statistically significant (F = 4.073, *p* = 0.045). It should be emphasized that both PR and FRS in nZAB were similar to those observed in the anthropogenic populations.

### 2.5. Determinants of Reproductive Success

The floral display influenced RS only in three populations, aBIA, a SOS, and aSUR. In the first population, IL was positively correlated with PR (r_S_ = 0.46, *p* < 0.05), while in the aSUR population, FRS depended on all floral display traits (r_S_ = 0.38–0.43, *p* < 0.05). In the aSOS population, negative correlations between SH, IL, and FRS were reported (Figure S2, Table 6).

The flower structures had a weak influence on RS (Figure S2, Table 6). Only 10 statistically significant correlations, mainly negative, were found among the 192 cases analyzed. FRS was influenced by DS, LF, WI, WH, and WE in aBIA (r_S_ = −0.49 − −0.59; *p* < 0.05), and by DP in nPOG2 (r_S_ = −0.37, *p* < 0.05). On the other hand, PR depended on WS in aSUR (r_s_ = 0.43; *p* < 0.05), on LI and LH in nZAB (r_S_ = −0.41 and r_S_ = −0.48; *p* < 0.05, respectively), and on WS and WE in nPOG1 (r_S_ = 0.50 and r_S_ = 0.52; *p* < 0.05, respectively).

RS also depended on some chemical components of the nectar (Figure S2, Table 6). Fructose and the sum of sugars were positively correlated only with PR in the aBIA population (r_S_ = 0.48, *p* < 0.05 and, r_S_ = 0.68, *p* < 0.05; respectively). A negative correlation was only observed between FRS and sucrose in nZAB (rs = −0.37, *p* < 0.05). We also found relationships between some AAs and reproductive parameters, but distinct AAs shaped RS in different populations, and their impact was independent of their participation. The highest number of such relationships (five AAs influenced PR and one influenced FRS) was observed in aBIA, while in aSUR and nZAB, a lack of correlations between AAs and RS was observed. Generally, 15 AAs have an impact on the RS, i.e., 13 on PR (i.e., Asp, Gly, Gln, Trp, Cit, BABA, Pro, Phe, Orn, GABA, Arg, Met, and AABA) and 4 on FRS (i.e., BABA, Tyr, Tau, and Met). Ten AAs were positively correlated with PR and only one with FRS in all populations.

## 3. Discussion

The level of RS reflects the interactions between plants and pollinators, which play a crucial role in the evolution and diversity of both groups. These relations can vary spatially and temporarily, and human-mediated disturbances of natural habitats are serious threats to the maintenance of these relationships. Under new circumstances, new connections between particular environmental components may arise. We expected that *E. helleborine* as nectariferous species, pollinated by a wide range of insects [22,27,29,76] and with the ability of spontaneous autogamy, as well as high selfing caused by pollinator behavior [22,25], will be characterized by a high level of RS. In fact, we found a great variation in RS in *E. helleborine* populations, and the lowest in two natural populations (nPOG1 and nPOG2), where, especially FRS, was below average for nectariferous orchids [75,77]. The differentiation in RS between populations of *E. helleborine* was also documented [22,28], although the differences noted by the second authors were significantly smaller than in the remaining studies. Substantial variation in the level of RS in our study suggests different interactions between orchids and their pollinators in particular places and huge pollinator deficiency in the two natural populations (nPOG1 and nPOG2) with the lowest RS. The scarcity of pollinators in these populations could be due to their location among peat bogs with sedge domination and low participation of flowering plants in the time when *E. helleborine* flowers or a complete lack of blossom cover in broadleaved forests (nPOG2). In contrast to the nPOG1 and nPOG2 populations, the third natural population (nZAB) is characterized by high PR and FRS. It is located on a mineral island dominated by an open area with other flowering plants and neighbors with areas covered by multi-species communities. The diversity and abundance of co-flowering species determine the richness of pollinators because more plants offer a wider spectrum of resources, thus attracting more insects and increasing the frequency of their visits [68,71,72]. The diversity of flowering plants provides both nutritional diversity of floral rewards and morphological diversity of flowers, which are important determinants of pollinator richness [78]. The wider spectrum of resources in the surroundings seems especially important in the case of generalist plants, such as *E. helleborine*, which depend on many pollinators belonging to distinct morphological and functional groups. The supposition about the importance of co-flowering species for pollinator abundance in particular communities may confirm the high level of pollinaria removal and fruit set in the anthropogenic populations, localized at the road edges, where other plants bloom together with *E. helleborine*. The pollinator assemblages along roads may be more diverse and abundant, causing a higher RS of the orchid. Phillips, et al. [2] documented that species richness and density of flowers and pollinators on road verges are similar or even greater than in other habitats in the surrounding landscape. This paper also shows that these areas are often hotspots for flowering plants and pollinators. Rewicz, et al. [22] found a higher taxonomic diversity of pollinators in anthropogenic than in natural populations of *E. helleborine* (19 vs. 14 families). Since some insects are highly mobile, they can move to anthropogenic populations from adjacent areas, in the case of our populations from multispecies meadows. One of the populations of *E. helleborine*, aSOS, is close to the Sośnia village with apiaries and gardens where ornamental plants are grown. It may influence the abundance of insects in this area and increase the RS of orchids. On the other hand, one of the highest RS we observed was in the aBIA population, although within its area, only *Melampyrum pratense* flowered. The high cover of this species may ensure an abundance of orchid pollinators because it attracts insects (for example, bumblebees and ants [79]) that are important for the pollination of *E. helleborine.* Generally, RS was higher in the anthropogenic than in the natural populations, similarly to the results of Rewicz, et al. [22]. This contrasts with the results of some studies on other orchids where the opposite pattern was found [21,80,81]. On the other hand, we observed a similar level of RS in the natural and anthropogenic populations of *E. palustris*, but communities in both habitats and their surroundings were rich in species, which flowered together with this orchid [30].

An important factor on which the level of RS depends is floral display. Higher plants with larger inflorescences usually attract more pollinators that visit more flowers on larger inflorescences, and in effect, these plants produce more fruits [43,82,83,84,85,86,87]. Floral display traits influenced RS only in three populations, i.e., aBIA, aSOS, and aSUR. Rewicz, et al. [22] explained the higher reproductive success of *E. helleborine* in anthropogenic habitats using the larger size of the plants. In our studies, the shoots were higher in the natural populations, while the number of flowers showed an opposite pattern, which contrasts with the results from Rewicz, et al. [88]. The lack of correlations between the RS parameters and the traits of the floral display in the remaining populations may indicate that the flowering shoots are visible enough to pollinators. They are usually the highest plants in the community.

RS often reflects the adaptation of plants to local partners. Therefore, an important determinant of RS is the mutual match between flower and pollinator structures, which is one of the crucial evolutionary mechanisms in shaping plant–pollinator interactions and one of the essential prerequisites for successful pollination [35,89]. Pollinators act as selection agents on flower morphology and influence plant fitness [37,41,45,90,91]. Our results show the weak importance of flower traits in shaping pollinaria removal and fruit set in *E. helleborine* populations. Only in 10 cases among the 192 analyzed did we find statistically significant correlations between them. We also observed a low influence of flower structure on RS for the other generalistic orchids, *Neottia ovata* and *E. palustris* [30,31]. Jacquemyn and Brys [92] suggest that a lack of strong selection of flower traits maintains their variation. This variation meets the requirements of many different pollinators, and thus could be the advantage of generalistic plants. The rare correlations between flower morphology and RS may indicate that the structure of *E. helleborine* flowers is well adapted to the size of a wide range of different pollinators. Despite the high diversity of potential pollinators of *E. helleborine*, their number in two natural populations (nPOG1 and nPOG2) appears insufficient, since RS is shaped at a low level in these places. Pollinator deficiency is recognized as the main cause of low RS in orchids [75]. Pollinator deficiency in these two natural populations caused no fruits to be developed on about one-third of the shoots in these populations, despite pollinaria being removed. Apart from pollinator deficiency, the cause of very low FRS may be the mismatch between flowers and pollinators. Inappropriate pollinators restrict correct flower penetration and lead to insufficient pollen supply, which can reduce plant pollination success. In other words, some insects visiting flowers and sucking nectar are not able to collect pollinia and/or place them on the stigma and thus are not effective pollinators. In this way, pollinaria are lost, as observed in other orchids [30,93]. It also suggests that the pollination of *E. helleborine* is partially random and that some insects present in the environment occasionally contribute to their pollination.

In nectariferous species, nectar traits are an important aspect of their reproductive strategy, and the effectiveness of pollination depends on the quantity and quality of the nectar. In articles dedicated to plant–pollinator interactions, the role of nectar chemistry in shaping RS has rarely been studied, so knowledge in this area is very limited. However, the available data show that different pollinators prefer nectar that is characterized by different properties. These preferences are related to nectar concentration, sugar ratios, and particular AAs [52,53,54,55,57,58,94]. Pollinator preferences translate into effects on plant fitness [63]. The nectar of *E. helleborine* is diluted. The review by Brzosko and Mirski [48] showed that generalistic orchids produce less concentrated nectar than specialists. Diluted nectar is probably an adaptation to pollinators that feed on easily accessible nectar [54]. We noted substantial variation among populations of *E. helleborine* in total sugar amount and concentration and in proportions between particular sugars. Large differences were especially evident between the anthropogenic and natural populations. Nectar in natural populations was more concentrated, had a higher amount of sugars, and sucrose dominated over hexoses.

Taking into account differentiation in nectar traits, as well as correlations between these parameters and RS in some populations, and supposing preferences of insects for a given type of nectar, we may suggest that different pollinator assemblages operate in particular places. This assumption is in line with the results of studies documenting the differentiation of pollinator assemblages in a different part of the geographical range [71], which was also the case for *E. helleborine* [22,25,76]. Bees are one of the important pollinators of *E. helleborine* [27,29]. Rewicz, et al. [22] found that they are more important pollinators in anthropogenic than in natural populations (20% vs. 9% of all visits). The increase in PR with an increase in glucose amount in nPOG2 and aBIA, and the decrease in PR with a higher percentage of sucrose in nPOG2 suggest that bees were not important pollinators in these places. However, the sum of sugars positively influenced PR in the aBIA and FRS in the nZAB populations. Bees prefer sucrose-rich and more concentrated nectar compared to other pollinators, reflecting their mode of nectar intake and energetic needs [95], although some bumblebees prefer less concentrated nectar [96]. Highly concentrated nectar ensures the carbohydrate required for bee foraging activities [97]. The nectars of Mediterranean species in a number of families are dominated by sucrose [55]. Brzosko and Mirski [48] reported that bee-pollinated orchids produce the most concentrated nectar (~40%). Pamminger, et al. [60] also observed a similar nectar concentration in bee-pollinated species (~41%), regardless of the continent and the type of community. The previous authors stated that a nectar concentration below 20% is of low quality for bees. Some studies document different preferences of bees for sucrose content. Peter and Johnson [98] suggest that the preferences of bees may vary from low- to medium-sucrose content since they are a heterogeneous group. On the other hand, Baker and Baker [52] found that short-tongued bees prefer hexose-rich nectar.

Rewicz, et al. [22] found that the main pollinators of *E. helleborine* were Syrphidae, which have a higher frequency in natural than in anthropogenic populations (53% vs. 26%). Syrphidae prefer hexose-dominant or hexose-rich nectar [50,52,53,54,66]. Flies preferences for hexoses were observed for the whole systematic group, e.g., Gentianales [99] and Balsaminaceae [57]. Syrphidae may be important *E. helleborine* pollinators in two anthropogenic populations (aSOS and aBIA), where hexoses were more abundant than sucrose. The other important pollinators of *E. helleborine*—wasps—prefer sucrose dominated nectar, which was documented, for example, by Petanidou [55] for plants in Mediterranean phrygana and by Brzosko and Mirski [48] for orchids. Rewicz, et al. [22] found a high frequency of Vespidae visits (22% in anthropogenic and 21% in natural populations). The wasps could be suggested as important pollinators in the aGON population, where a negative relationship between PR and the percentage of fructose was observed. In aBIA, ants may be crucial *E. helleborine* pollinators. In this place, *Melampyrum pratense* was abundant as the only one co-flowering species. It is mainly pollinated by ants and bumblebees [79], which are also important pollinators of *E. helleborine*. Rewicz, et al. [100] found that the frequency of Formicidae visits equaled 6% in natural and 9% in anthropogenic populations. We often observed ants on inflorescences. They prefer hexoses or even sucrose-free nectar because they cannot assimilate this sugar due to a lack of invertase, and sucrose-rich nectar can be toxic for some generalist insects [101]. This information, together with the result that PR in this population depends on the amount of glucose, may highlight the suggestion that ants are important pollinators of *E. helleborine* in the aBIA population. An interesting case represents the nZAB population with one of the highest levels of RS. Simultaneously, the sucrose amount in the nectar was the highest among the populations studied, but we noted a negative correlation between sucrose and FRS in this population. On the one hand, these results may suggest the presence of pollinators that prefer sucrose, but, on the other hand, fruiting might be even higher if the nectar was adapted to pollinators preferring nectar with lower participation of sucrose.

Other important components of flower nectar are AAs, which, as a crucial source of building material, influence the growth, reproduction, and survival of feeding animals [50,67,102,103]. Some AAs act as manipulators of pollinator behavior [104,105]. *E. helleborine* nectar, similar to other generalistic orchids [30,31], is rich in AAs (20 proteogenic and 7 non-proteogenic AAs were found). The nectar of specialists is composed of a lower number of AAs, that is, 20 in *Gymnadenia conopsea* [32,51] and 23 in *Platantherans* [64]. The high number of AAs in nectar may be explained as a richer offer directed to the wide range of pollinators and could be an adaptation for generalists. We also found a wide range in the amount and percentage of AAs in *E. helleborine* populations with a significantly higher participation of some (Asp, Gln, Gly, Pro, and β-Ala) in the anthropogenic than in the natural populations, as in the case of *E. palustris* [30]. In the majority of cases, different AAs influenced the RS parameters in each population. Furthermore, the influence of AAs on RS in particular populations was independent of their participation in the nectar. For example, despite the clear domination of Glu in all populations (26.7–37.7%), it did not correlate with the RS in any population. Glu was also one of the most abundant AAs in the nectar of other generalist orchids, *N. ovata* and *E. palustris* [30,31]. This AA is necessary for energetically exhaustive flights, influences pollinator behavior, and plays a role in parasitoid rejection [32,50,51,54]. The second AA with an important participation in the nectar of *E. helleborine* was Asp (8.1–17.8%). It also belonged to the most abundant AA in *N. ovata* nectar [31]. This AA influences pollinator behavior and is known as a general repellent [66]. The amount of Asp was correlated with PR in two populations, positively in aBIA and negatively in nPOG2. Examples of opposite directions in these influences suggest that different pollinators operate in these places. The most frequent in *E. helleborine* nectar were AAs with little participation (max. ca. 9% in some populations in the case of Asn, Gln, and Thr). The increase in Gln increased PR in aBIA, and the increase in Tyr decreased FRS in aCAR. Gln, similar to Glu, is needed for energetically exhaustive flights, while Thr acts as a general repellent [66]. The participation of particular AAs in a given population, their correlation with RS, and knowledge of the preferences to them can help identify the main pollinators. For example, the higher amount of Phe and Trp in aSOS and aBIA, to which Syrphidae show strong preferences [66], could suggest that they are important pollinators in these places. This is also consistent with other results, namely, the domination of hexoses over sucrose (which prefer Syrphidae) in the aSOS and aBIA populations.

We would like to point out the important role of non-proteogenic AAs in shaping plant–pollinator interactions [104]. Their participation varied significantly between the populations, and the lowest was in two natural populations (nPOG1 and nPOG2) with the lowest RS and the highest (almost three times) in the third natural population (nZAB). One of the most common AAs in plants, Pro, had a greater participation in the anthropogenic populations (excluding aSUR) than in the natural populations. A similar pattern was observed in *E. palustris* [30]. This AA is important for many pollinators, especially Hymenoptera [50,54]. Pro plays many functions—it rewards pollinators, propels the lift phase of the flight [106,107], can be used in energy production [50], and stimulates insect salt receptors, which initiate feeding [58,65,67]. Moreover, the accumulation of Pro may help plants respond to stress factors [106]. This function could explain the increased participation of Pro in the anthropogenic populations, where changed habitats are more stressful for plants. Pro was present in all populations, but only in nPOG1 and aCAR was it positively correlated with PR. The other non-proteogenic AA with greater participation in the anthropogenic populations than in the natural populations was Gly. It was positively correlated with PR in aBIA and negatively correlated with FRS in aSOS. Gly activates a feeding response in honeybees [95] and can deter honeybees [72]. In light of this information, its negative influence on FRS in aSOS, where there are few apiaries in the village, is rather strange because plants that offer unsuitable nectar for bees eliminate important potential pollinators. Apparently, plants in this population produce nectar for other pollinators, since sucrose participation in aSOS is also one of the lowest, thus also not the best for honeybees. The statement that bees are not key pollinators, at least in some populations of *E. helleborine*, may confirm other results: a negative correlation between PR and sucrose and the preferences for glucose in nPOG2 and for glucose in aBIA. It is worth highlighting the absence of GABA in all natural populations of *E. helleborine.* This AA was absent in *Platanthera bifolia* and present in only one population of *P. chlorantha* [64]. In nPOG1, the lack of β-Ala was observed. However, in three anthropogenic populations (aCAR, aGON, and aSUR) Cit was not found and in aBIA, Met was not found. The participation of all these AAs in the remaining populations was less than 1%. GABA influences the insect nervous system and muscle activity [104,108] and improves honeybee specific retention of memory [109]. Its higher amount increased PR in the aCAR populations. The lack of GABA in natural populations can again suggest that bees are not important pollinators of *E. helleborine,* since bees (and bumblebees) are among pollinators of plants with a higher concentration of GABA in nectar [104]. Met plays a potential role in parasitoid rejection [54]. This AA negatively influenced PR and FRS in the nPOG2 population. The other non-proteogenic AA, BABA, contributes to protecting plants from pathogens [104,107,110]. It was present in all populations of *E. helleborine,* but only in aBIA was it correlated (positively) with FRS. The lack of significant correlations between particular AAs and reproductive success does not indicate that they do not influence RS. Nepi [104] reported that insects are forced to move from flower to flower due to the combined phagostimulatory activity of Pro, Phe, and GABA to maintain a high feeding rate.

The composition of AAs in nectar influences pollinator taste perception and behavior through specific neurological or phago-stimulating pathways [54]. Gardener and Gillman [50] suggest that the taste of nectar may be more important than its nutritional value. This nectar trait can attract and discourage pollinators, and taste perception differs between different pollinators [52,107]. Our results suggest that pollinating insects were sensitive to AAs from taste classes I and IV in the aBIA populations. However, in the aCAR and nPOG1 populations, pollinator preferences for AA from taste class III were observed (Pro positively influenced PR in both populations and Trp in nPOG1). This highlights the role of these AAs in the behavior of *E. helleborine* visitors in these places and in shaping reproductive success. Although class III positively influenced PR in nPOG1, fruiting in this population was approximately two times lower than in aCAR. This indicates that only part of nectar consumers are pollinators in nPOG1. It may also suggest pollinator deficiency in this population. This is in agreement with other results suggesting that distinct insect assemblages operate in different habitats of *E. helleborine.*

The chemistry of nectar can be strongly influenced by abiotic factors, including soil parameters [63]. Gardener and Gillman [50] and Gijbels, et al. [32] demonstrated that the addition of nutrients significantly increased the concentration of AAs in the nectar of *Agrostemma githago* and *Gymnadenia conopsea*. On the other hand, Brzosko, et al. [31] found that the carbon:nitrogen ratio influenced the composition of sugars and AA in the nectar of *N. ovata*. The addition of nutrients to a natural population of *Ipomopsis aggregata* increased nectar volume by 38% and pollen receipt, a proxy of pollinator visitation, by 26% compared to control plants [111]. The answer to the question of how distinct soil characters in natural and anthropogenic habitats shape the nectar chemistry of *E. helleborine* and whether it influences RS requires detailed studies.

## 4. Materials and Methods

### 4.1. Study Area

This study was carried out in July and August 2021 using eight *E. helleborine* populations in northern Poland. Three of them, that is, nPOG1, nPOG2, and nZAB, were localized in natural habitats in the Biebrza National Park on a mineral island among one of the largest areas of peatlands in Europe. The five other populations, that is, aBIA, aCAR, aGON, aSOS, and aSUR, which represent the anthropogenic populations, existed on the verges of roads with differentiated floristic composition of plant communities (Table 7). The first four anthropogenic populations were located along roads in the near vicinity of Biebrza National Park, while the aSUR population was more distant from the others (more than 100 km) and was located closer to the Narew River.

### 4.2. Fieldwork and Floral Trait Measurements

In each population, 30 to 32 flowering individuals were chosen and marked. Some plants were damaged; therefore, the final sample size was lower in some populations. In total, 221 plants and 1909 flowers were included in the study. In the field, the traits of the flower display (the height of the inflorescence length, and the number of flowers) were quantified. The few unpollinated flowers from each inflorescence were collected, depending on the size of the inflorescence and the number of open flowers. All of them were used for the analyses of nectar composition, but the values of nectar parameters are given per flower. The two lowest flowers were used for the measurement of the morphological variables using an optodigital microscope DSX110 (Olympus Life Science, Waltham, MA, USA). The full names of the abbreviations of measured flower parameters are presented in Table 1. The isthmus area, considered as a measure of the nectar quantity, was calculated as the product of LI and WI. Flower traits of an individual are given as an average of two measurements. The flowers of all populations were collected in sunny and hot weather (the temperature was about 30 °C).

To assess the level of reproductive success (RS), on marked shoots, the number of flowers per inflorescence was counted. During capsule maturation, male reproductive success (pollinaria removal—PR) and female reproductive success (FRS) were quantified. FRS was evaluated as the number of developed fruits to the number of flowers in the inflorescence. PR was determined as the number of pollinaria removed to the total number of pollinaria for each inflorescence. Both FRS and PR were given as percentages. We also calculated the efficiency of pollination in particular populations as the ratio of PR to FRS: the higher the index value, the lower the pollination efficiency.

### 4.3. Nectar Analysis

#### 4.3.1. Nectar Isolation

The flower nectar isolation was performed using a water-washing method. Five flowers per sample were placed in a 2 mL Eppendorf tube containing 1 mL of distilled water and shaken in a laboratory thermomixer (120 rpm, 21 °C, 45 min; Eppendorf Corporate, Hamburg, Germany) for nectar efflux. The flowers were removed from the tubes, and the mixture of water and nectar was evaporated to dryness using a centrifugal vacuum concentrator (45 °C, Labconco CentriVap Micro IR, Kansas City, MO, USA). The obtained pellet was dissolved in 20 µL of distilled water and then transferred to the centrifuge tube with a filter and centrifuged to remove impurities (9000× *g*, 5 min; MPW-55, MPW Med. Instruments, Gliwice, Poland). The purged extract was collected in a glass vial with a 250 µL insert with polymer feet.

#### 4.3.2. Sugar and Amino Acid Determination

The determination and quantification of sugars and AAs were performed using the high-performance liquid chromatography (HPLC) method. An Agilent 1260 Infinity Series HPLC apparatus (Agilent Technologies, Inc., Santa Clara, CA, USA) with quaternary pump with in-line vacuum degasser, thermostatic column, and refrigerated autosampler with autoinjector sample loop was used.

The standards of sugars, AAs, NaOH, NaH_2_PO_4_, OPA, and FMOC were purchased from Sigma-Aldrich (St. Louis, MO, USA). Water, ACN, EtOH, and MeOH were purchased from Merck KGaA (Darmstadt, Germany).

For the sugar analysis, a ZORBAX carbohydrate analysis column (4.6 mm × 250 mm, 5 µm) (Agilent Technologies, Inc., Santa Clara, CA, USA) was applied at a temperature of 30 °C and a refractive index detector was applied. The mobile phase was a solution of acetonitrile/water (70:30, *v*/*v*) at a flow rate of 1.4 mL/min. The injection volume was 10 µL. The total analysis time was 15 min.

Meanwhile, for AA detection, an automatic derivatization program was set. Therefore, *o*-phthalaldehyde (OPA), and 9-fluorenylmethyl chloroformate (FMOC) reagents were used for the derivatization of primary and secondary AAs. The Agilent Zorbax Eclipse Plus C_18_ column (4.6 × 150 mm, 5 µm) column (Agilent Technologies, Inc., Santa Clara, CA, USA) at a temperature of 40 °C was used to separate individual AAs. Detection of primary AAs was performed using a photodiode array detector at 388 nm, while detection of secondary AAs was performed using a fluorescence detector with an excitation wavelength of 266 nm and an emission wavelength of 305 nm. The injection volume was 5 µL; the flow rate was 1 mL/min. Eluent A of the mobile phase was 40 mM NaH_2_PO_4_ (pH 7.8, adjusted with a solution of 10 M NaOH), while eluent B was a mixture including acetonitrile/methanol/water (45:45:10, *v*/*v*/*v*). The gradient was the following: 0–5 min, 100–90% A; 5–25 min, 90–59.5% A; 25–30 min, 59.5–37% A; 30–35 min, 37–18% A; 35–37 min, 18–0% A; 37–40 min, 0% A; and 40–43 min, 100% A.

The analytical data were integrated using Agilent OpenLab CDS ChemStation software (Agilent Technologies, Inc., Santa Clara, CA, USA) for liquid chromatography systems. The identification of sugars and AAs was performed by comparing the retention times of individual sugars and AAs in the reference solution versus the test solution. The concentration of these compounds was assayed on comparisons of peak areas obtained for the samples investigated with those of the reference solutions.

### 4.4. Statistical Analysis

The R programming language/statistical environment was used to perform all statistical calculations and analyses, as well as to prepare graphics and transform data for tabular representation [112]. The floral display, flower structure, sugars, and AAs datasets were analyzed with the Kruskal–Wallis test followed by a pairwise Wilcoxon rank sum test with the Benjamini–Hochberg adjustment. The Shapiro–Wilk and Bartlett tests were also used primarily to check for a possible ANOVA application. Furthermore, a set of descriptive statistics (mean, standard error, quartiles, and interquartile range) was calculated for the floral display, flower structure, sugars, and AAs. For all tests, the significance level was α = 0.05. To check if a monotonic relationship exists between floral display, flower structure parameters, and nectar composition, Spearman’s rank correlations were calculated using the ‘rcorr’ function from the ‘Hmisc’ package. Correlations were considered significant for *p* < 0.05 [113].

To analyze the effect of AAs on insect chemoreceptors, all identified and determined AAs were grouped into four classes (the full names of the abbreviations are presented in Table 4): I. Asn, Gln, Ala, Cys, Gly, Ser, Thr, and Tyr (without an effect on the chemoreceptors of fly); II. Arg, Asp, Glu, His, and Lys (inhibition of fly chemoreceptors); III. Pro (stimulation of the salt cell); and IV. Ile, Leu, Met, Phe, Trp, and Val (ability to stimulate the sugar cell) and presented as a ternary plot [114]. Principal component analysis (PCA) was used to simplify the exploration of AAs. To build the PCA model, the ‘FactoMineR’ package was used [115]. Two tests were performed that indicate the suitability of the AA dataset for structure detection and reduction. Bartlett’s sphericity test and the Kaiser–Mayer–Olkin test of factorial adequacy (‘psych’ package) [116]. Unit variance scaling of the data was applied; therefore, PCA was performed on a correlation matrix rather than on a covariance matrix. The number of principal components to retain was selected with the help of Cattell’s and cumulative percentage of explained variance rules (Figures S3 and S4). All biplots were created using the ‘factoextra’ package [117].

## 5. Conclusions

Our study fits into one of the most important problems of recent ecology and evolutionary biology, which enable us to understand the mechanisms and processes shaping plant–pollinator interactions. The results obtained document how the variation in flower traits is distributed in natural and anthropogenic populations of *E. helleborine* and how they shape the reproductive success of orchids. We found that nectar properties had a greater impact on FRS and PR than flower morphology, which influenced RS only in 10 of the 192 cases analyzed. The results of our study increase the knowledge on nectar chemistry, especially in orchids, for which nectar properties were rarely studied. They are useful for explaining the importance of nectar composition for RS. Only a few studies have touched on this problem, despite the fact that the role of nectar for RS is unquestionable. Moreover, to our knowledge, this is the second article (the first was Brzosko, et al. [30]) that reports a comparison of nectar chemistry between natural and anthropogenic orchid populations. *E. helleborine* nectar is diluted similar to other generalists, e.g., *N. ovata* and *E. palustris* [30,31]. It seems that a low nectar concentration increases its availability for differentiated pollinator groups. Furthermore, the relatively weak influence of nectar chemistry on PR and FRS suggests that *E. helleborine*, as a generalist, dedicates nectar to a wide range, mainly unspecialized pollinators, so it should produce nectar suitable for their differentiated mouth apparatus and equally differentiated dietary needs. Therefore, at the species level, the plants offer rich nectar that includes a wide range of components with high variability among populations (both in terms of their number and participation), which exist in different communities and thus relates to different insect assemblages. This indicates that this species did not evolve nectar traits that filter flower visitors, thus, they are not dedicated to a certain group of pollinators. However, some nectar components were correlated with RS parameters; therefore, our results at least partially show that nectar traits can explain the variation in fruiting success between populations. Another important cause of RS differentiation could be the composition of plant communities in which *E. helleborine* grows. The diversity of flowering plants provides both nutritional diversity of floral rewards and morphological diversity of flowers, which are important determinants of pollinator richness. The lack of co-flowering species during the flowering period of orchids is suggested to be a factor that decreases the level of RS in some populations.

We found differences in nectar composition between anthropogenic and natural populations. In the first group, the total amount of sugars and the participation of sucrose were significantly lower than in the second group. Additionally, in natural populations, the domination of sucrose over hexoses was observed. We also noted differences between populations from two habitat types in the composition of AAs in the nectar. These differences suggest that in anthropogenic habitats, orchids may respond to different pollinator assemblages than in natural populations through modification of nectar properties. Variation in flower traits could be an adaptation to the most effective pollinators, being an answer to the requirements of their specific assemblages present in a given environment.

## Figures and Tables

**Table 1:** Variation in floral display and flower structure in natural and anthropogenic populations of *Epipactis helleborine*. Data represent the mean (x) ± standard error (SE), lower quartile (Q_1_), median (Q_2_), upper quartile (Q_3_), interquartile range (IQR), and biological replicate (n). Different letters indicate statistically significant differences according to the pairwise Wilcoxon rank sum test with the Benjamini–Hochberg adjustment (*p* < 0.05).

Parameter	Statistic	Population							
		aBIA	aCAR	aGON	aSOS	aSUR	nPOG1	nPOG2	nZAB
**Floral display**									
Shoot height (SH) (cm)	$\overset{-}{x}$ ± SE	50.84 ± 2.73	71.19 ± 3.34	64.83 ± 3.44	58.78 ± 2.39	58.79 ± 3.06	69.19 ± 2.28	62.55 ± 2.07	75.07 ± 1.96
	Q_1_	42.50	67	52.62	48.50	47.88	61	59	68
	Q_2_ (IQR)	48 (16) ^a^	69 (12) ^bc^	59.5 (26) ^bd^	62 (19.50) ^ad^	59 (18.62) ^ad^	68 (16.50) ^bc^	61 (11) ^bd^	73 (14.50) ^c^
	Q_3_	58.5	79.00	78.62	68	66.50	77.50	70.00	82.50
	n	19	21	26	27	28	27	29	29
Inflorescence length (IL) (cm)	$\overset{-}{x}$ ± SE	15.68 ± 1.39	17.05 ± 1.31	17.94 ± 1.73	16.59 ± 0.92	18.02 ± 1.18	20.37 ± 1.32	15.79 ± 0.84	20.43 ± 0.70
	Q_1_	11	12	10.88	13.50	12	16	13	18
	Q_2_ (IQR)	15 (6) ^a^	17 (9) ^abc^	16 (13.62) ^abc^	16 (6.50) ^ab^	18 (12) ^abc^	19 (8) ^bc^	15 (6) ^a^	21 (5) ^c^
	Q_3_	17	21	24.5	20	24	24	19	23
	n	19	21	26	27	28	27	29	29
Number of flowers (NF)	$\overset{-}{x}$ ± SE	8.50 ± 0.25	8.41 ± 0.34	8.90 ± 0.31	8.93 ± 0.33	9.33 ± 0.19	8.93 ± 0.28	7.03 ± 0.30	9.13 ± 0.29
	Q_1_	7.25	7	8	8	8.25	8	6	8
	Q_2_ (IQR)	8.50 (2.50) ^a^	8.5 (3) ^ab^	10 (2)^ab^	9 (2) ^ab^	10 (1.75) ^b^	10 (2) ^ab^	6.5 (2) ^c^	10 (2) ^ab^
	Q_3_	9.75	10	10	10	10	10	8	10
	n	22	22	29	28	30	28	32	30
**Flower structure**									
Length of lateral sepal (LS) (mm)	$\overset{-}{x}$ ± SE	11.53 ± 0.29	12.54 ± 0.24	11.29 ± 0.27	12.53 ± 0.23	10.72 ± 0.17	12.54 ± 0.22	11.36 ± 0.25	11.98 ± 0.19
	Q1	10.52	11.75	11.07	11.55	10.13	11.83	10.34	11.23
	Q_2_ (IQR)	11.18 (1.86) ^ab^	12.09 (1.91) ^c^	11.61 (0.8) ^a^	12.64 (1.91) ^c^	10.54 (1.27) ^b^	12.59 (1.56) ^c^	11.14 (1.84) ^ab^	11.96 (1.32) ^ac^
	Q_3_	12.38	13.66	11.87	13.46	11.40	13.39	12.18	12.55
	n	22	22	29	28	30	28	32	30
Width of lateral sepal (WS) (mm)	$\overset{-}{x}$ ± SE	5.22 ± 0.19	5.61 ± 0.16	5.51 ± 0.13	5.74 ± 0.12	5.08 ± 0.11	5.61 ± 0.15	4.82 ± 0.10	5.80 ± 0.10
	Q_1_	4.49	5.21	5.26	5.32	4.71	5.08	4.51	5.48
	Q_2_ (IQR)	4.98 (1.37) ^ab^	5.68 (0.86) ^ac^	5.63 (0.66) ^ac^	5.76 (0.91) ^c^	5.12 (0.86) ^b^	5.50 (0.83) ^ac^	4.78 (0.54) ^b^	5.70 (0.84) ^c^
	Q_3_	5.86	6.07	5.92	6.23	5.57	5.91	5.05	6.32
	n	22	22	29	28	30	28	32	30
Distance between petals (DP) (mm)	$\overset{-}{x}$ ± SE	14.69 ± 0.36	15.96 ± 0.38	14.75 ± 0.28	15.5 ± 0.42	13.86 ± 0.41	15.40 ± 0.36	14.64 ± 0.24	15.41 ± 0.38
	Q_1_	13.49	14.86	13.72	13.69	12.27	14.25	103.85	14.35
	Q_2_ (IQR)	14.62 (2.23) ^ab^	15.69 (2.35) ^a^	14.65 (2.15) ^ab^	15.79 (2.98) ^ab^	13.17 (3.47) ^b^	15.38 (2.07) ^ab^	14.78 (1.80) ^ab^	14.89 (2.21) ^ab^
	Q_3_	15.72	17.21	15.87	16.67	15.74	16.32	15.65	16.57
	n	22	22	29	28	30	28	32	30
Distance between sepals (DS) (mm)	$\overset{-}{x}$ ± SE	19.65 ± 0.57	20.6 ± 0.44	18.83 ± 0.39	20.52 ± 0.42	17.42 ± 0.32	20.49 ± 0.50	18.35 ± 0.40	19.93 ± 0.35
	Q_1_	17.30	19.24	17.92	18.56	16.34	18.66	16.65	19.27
	Q_2_ (IQR)	19.09 (4.04) ^abc^	20.24 (2.16) ^a^	18.91 (2.12) ^bc^	20.48 (3.84) ^a^	17.42 (1.77) ^d^	20.36 (2.94) ^a^	17.90 (3.20) ^bd^	19.94 (1.56) ^ac^
	Q_3_	21.34	21.39	^a^ 20.04	22.40	18.11	21.61	19.85	20.83
	n	22	22	29	28	30	28	32	30
Length of flower (LF) (mm)	$\overset{-}{x}$ ± SE	12.25 ± 0.35	12.35 ± 0.31	12.87 ± 0.22	12.70 ± 0.28	11.9 ± 0.22	13.21 ± 0.35	12.32 ± 0.22	13.01 ± 0.20
	Q_1_	11.16	11.54	12.15	11.66	10.91	12.63	11.67	12.40
	Q_2_ (IQR)	11.93 (2.33) ^abc^	12.00 (1.53) ^abc^	12.71 (1.53) ^ab^	12.21 (1.93) ^abc^	12.04 (1.82) ^c^	13.37 (1.48) ^a^	12.21 (1.35) ^bc^	12.97 (1.05) ^ab^
	Q_3_	13.49	13.07	13.68	13.59	12.73	14.12	13.02	13.45
	n	22	22	29	28	30	28	32	30
Length of isthmus (LI) (mm)	$\overset{-}{x}$ ± SE	1.97 ± 0.06	2.15 ± 0.06	2.07 ± 0.04	2.22 ± 0.05	1.89 ± 0.04	2.18 ± 0.06	2.16 ± 0.06	2.00 ± 0.05
	Q_1_	1.81	2.10	1.93	2.11	1.73	2.01	1.89	1.88
	Q_2_ (IQR)	1.94 (0.32) ^ab^	2.13 (0.10) ^ac^	2.05 (0.31) ^ac^	2.24 (0.25) ^c^	1.90 (0.30) ^b^	2.15 (0.37) ^ac^	2.19 (0.50) ^ac^	2.04 (0.27) ^ab^
	Q_3_	2.13	2.20	2.24	2.36	2.03	2.38	2.40	2.15
	n	22	22	29	28	30	27	31	30
Width of isthmus (WI) (mm)	$\overset{-}{x}$ ± SE	2.54 ± 0.10	2.57 ± 0.08	2.77 ± 0.09	2.86 ± 0.07	2.75 ± 0.09	2.65 ± 0.07	2.49 ± 0.06	2.84 ± 0.06
	Q_1_	2.20	2.37	2.65	2.65	2.40	2.36	2.30	2.65
	Q_2_ (IQR)	2.36 (0.61) ^ab^	2.67 (0.49) ^abc^	2.79 (0.48) ^ac^	2.88 (0.40) ^c^	2.65 (0.72) ^abc^	2.60 (0.49) ^abc^	2.47 (0.34) ^b^	2.82 (0.38) ^c^
	Q_3_	2.81	2.86	3.13	3.04	3.12	2.85	2.64	3.03
	n	22	22	29	28	30	27	31	30
Length of hypochile (LH) (mm)	$\overset{-}{x}$ ± SE	3.96 ± 0.07	4.17 ± 0.08	4.06 ± 0.06	4.27 ± 0.07	3.88 ± 0.06	4.67 ± 0.09	4.16 ± 0.10	4.42 ± 0.06
	Q_1_	3.66	3.95	3.87	4.06	3.61	4.42	3.77	4.17
	Q_2_ (IQR)	3.88 (0.47) ^ab^	4.15 (0.40) ^ac^	4.12 (0.44) ^abc^	4.18 (0.48) ^cd^	3.80 (0.55) ^b^	4.53 (0.43) ^e^	4.10 (0.76) ^ac^	4.40 (0.54) ^de^
	Q_3_	4.14	4.35	4.31	4.54	4.16	4.85	4.53	4.70
	n	22	22	29	28	30	27	31	30
Width of hypochile (WH) (mm)	$\overset{-}{x}$ ± SE	8.18 ± 0.16	8.58 ± 0.12	8.17 ± 0.11	8.53 ± 0.15	8.01 ± 0.11	8.72 ± 0.16	8.21 ± 0.18	8.57 ± 0.15
	Q_1_	7.64	8.02	7.79	7.87	7.56	8.15	7.48	7.99
	Q_2_ (IQR)	8.32 (1.05) ^ab^	8.53 (0.98) ^a^	8.09 (0.81) ^ab^	8.55 (1.15) ^ab^	7.96 (0.89) ^b^	8.70 (1.05) ^a^	7.98 (1.31) ^ab^	8.40 (1.28) ^a^
	Q_3_	8.69	9.01	8.60	9.02	8.44	9.20	8.79	9.27
	n	22	22	29	28	30	27	31	30
Length of epichile (LE) (mm)	$\overset{-}{x}$ ± SE	4.22 ± 0.12	4.41 ± 0.09	4.11 ± 0.08	4.27 ± 0.13	4.13 ± 0.07	4.04 ± 0.1	4.05 ± 0.11	4.15 ± 0.11
	Q_1_	3.88	4.11	3.76	3.84	3.83	3.66	3.67	3.70
	Q_2_ (IQR)	4.24 (0.90) ^a^	4.40 (0.65) ^a^	4.00 (0.57) ^a^	4.27 (0.83) ^a^	4.10 (0.62) ^a^	4.17 (0.81) ^a^	3.87 (0.62) ^a^	4.12 (0.90) ^a^
	Q_3_	4.78	4.76	4.33	4.67	4.45	4.47	4.30	4.59
	n	22	22	29	28	30	27	31	30
Width of epichile (WE) (mm)	$\overset{-}{x}$ ± SE	3.55 ± 0.13	3.89 ± 0.10	4.09 ± 0.10	4.27 ± 0.12	3.98 ± 0.11	4.27 ± 0.15	3.6 ± 0.09	4.33 ± 0.09
	Q_1_	3.14	3.84	3.83	3.86	3.52	3.79	3.26	4.08
	Q_2_ (IQR)	3.30 (0.82) ^ab^	4.00 (0.27) ^ac^	4.20 (0.56) ^cd^	4.21 (0.77) ^cd^	4.06 (0.84) ^c^	4.19 (0.89) ^cd^	3.74 (0.62) ^b^	4.36 (0.55) ^d^
	Q_3_	3.96	4.11	4.39	4.63	4.36	4.68	3.88	4.63
	n	22	22	29	28	30	27	31	30
Length of labellum (LL) (mm)	$\overset{-}{x}$ ± SE	4.66 ± 0.15	5.01 ± 0.10	5.00 ± 0.08	4.85 ± 0.11	4.58 ± 0.10	4.97 ± 0.10	4.65 ± 0.09	5.12 ± 0.09
	Q_1_	4.11	4.74	4.72	4.52	4.15	4.54	4.36	4.80
	Q_2_ (IQR)	4.47 (0.97) ^abc^	4.98 (0.52) ^ad^	4.94 (0.54) ^d^	4.82 (0.74) ^abcd^	4.56 (0.64) ^b^	4.98 (0.89) ^acd^	4.77 (0.58) ^bc^	5.06 (0.66) ^d^
	Q_3_	5.08	5.25	5.26	5.26	4.79	5.43	4.94	5.46
	n	22	22	29	28	30	27	31	30

**Table 2:** Sugar concentration (µM) in *Epipactis helleborine* nectar. Data represent the mean (x) ± standard error (SE), lower quartile (Q_1_), median (Q_2_), upper quartile (Q_3_), interquartile range (IQR), and biological replicate (n). Different letters indicate statistically significant differences according to the pairwise Wilcoxon rank sum test with the Benjamini–Hochberg adjustment (*p* < 0.05).

Sugar	Statistic	Population							
		aBIA	aCAR	aGON	aSOS	aSUR	nPOG1	nPOG2	nZAB
		n = 22	n = 22	n = 29	n = 28	n = 30	n = 28	n = 32	n = 29
Glucose	$\overset{-}{x}$ ± SE	15.89 ± 0.29	10.53 ± 0.15	14.02 ± 0.13	17.55 ± 0.13	13.96 ± 0.2	14.28 ± 0.25	16.89 ± 0.19	14.4 ± 0.29
	Q_1_	15.07	10.04	13.52	17.21	13.26	13.69	16.15	13.06
	Q_2_ (IQR)	15.57 (1.2) ^a^	10.55 (0.72) ^b^	14.09 (1.01) ^c^	17.45 (0.85) ^d^	13.61 (1.30) ^c^	14.24 (1.39) ^c^	16.76 (1.51) ^e^	14.41 (2.10) ^c^
	Q_3_	16.27	10.76	14.53	18.06	14.56	15.08	17.66	15.16
Fructose	$\overset{-}{x}$ ± SE	19.41 ± 0.23	12.11 ± 0.24	16.5 ± 0.2	20.4 ± 0.16	16.36 ± 0.23	14.59 ± 0.25	17.17 ± 0.2	14.74 ± 0.17
	Q_1_	18.52	11.33	16.11	20.04	15.68	13.91	16.75	14.07
	Q_2_ (IQR)	19.63 (1.60) ^a^	11.82 (1.68) ^b^	16.63 (1.15) ^c^	20.58 (0.79) ^d^	16.16 (1.39) ^c^	14.72 (1.48) ^e^	16.98 (0.87) ^f^	14.7 (1.15) ^e^
	Q_3_	20.12	13.01	17.26	20.83	17.07	15.38	17.62	15.22
Sucrose	$\overset{-}{x}$ ± SE	12.61 ± 0.2	14.77 ± 0.19	16.17 ± 0.11	14.02 ± 0.22	16.21 ± 0.12	26.55 ± 0.31	26.79 ± 0.18	38.34 ± 0.23
	Q_1_	12.43	14.21	15.91	13.13	15.82	25.69	26.08	37.81
	Q_2_ (IQR)	12.68 (0.6) ^a^	14.75 (0.91) ^b^	16.29 (0.61) ^c^	13.61 (1.74) ^d^	16.18 (0.82) ^c^	26.18 (1.31) ^e^	26.58 (1.41) ^e^	38.03 (1.09) ^f^
	Q_3_	13.03	15.12	16.53	14.87	16.63	26.99	27.49	38.9
Sum of sugars	$\overset{-}{x}$ ± SE	47.92 ± 0.54	37.41 ± 0.44	46.70 ± 0.28	51.97 ± 0.26	46.52 ± 0.38	55.42 ± 0.54	60.85 ± 0.39	67.48 ± 0.53
	Q_1_	45.66	35.83	45.86	50.87	45.32	54.02	59.45	65.42
	Q_2_ (IQR)	47.88 (3.34) ^a^	36.68 (2.85) ^b^	46.58 (2.09) ^a^	51.75 (1.73) ^c^	46.05 (2.28) ^a^	55.14 (3.51) ^d^	60.62 (2.85) ^e^	66.97 (3.87) ^f^
	Q_3_	49	38.68	47.95	52.61	47.60	57.53	62.30	69.29

**Table 3:** The percentage content of sugars and their ratios in *Epipactis helleborine* nectar. Data represent the mean (x) ± standard error (SE), lower quartile (Q_1_), median (Q_2_), upper quartile (Q_3_), interquartile range (IQR), and biological replicate (n). Different letters indicate statistically significant differences according to the pairwise Wilcoxon rank sum test with the Benjamini–Hochberg adjustment (*p* < 0.05).

Sugars	Statistic	Population							
		aBIA	aCAR	aGON	aSOS	aSUR	nPOG1	nPOG2	nZAB
		n = 22	n = 22	n = 29	n = 28	n = 30	n = 28	n = 32	n = 29
Glucose (GLU) content in nectar (*w*/*v*) (%)	$\overset{-}{x}$ ± SE	0.33 ± 0	0.24 ± 0	0.27 ± 0	0.32 ± 0	0.27 ± 0	0.18 ± 0	0.20 ± 0	0.14 ± 0
	Q_1_	0.32	0.23	0.26	0.31	0.26	0.18	0.20	0.14
	Q_2_ (IQR)	0.33 (0.02) ^a^	0.24 (0.02) ^b^	0.27 (0.02) ^c^	0.32 (0.02) ^d^	0.27 (0.02) ^c^	0.19 (0.01) ^e^	0.20 (0.01) ^f^	0.14 (0.01) ^g^
	Q_3_	0.34	0.25	0.28	0.33	0.28	0.19	0.21	0.15
	$\overset{-}{x}$ ± SE	0.28 ± 0				0.18 ± 0			
	Q_1_	0.26				0.15			
	Q_2_ (IQR)	0.28 (0.06) ^a^				0.19 (0.05) ^b^			
	Q_3_	0.32				0.20			
Fructose (FRU) content in nectar (*w*/*v*) (%)	$\overset{-}{x}$ ± SE	0.27 ± 0	0.21 ± 0	0.23 ± 0	0.27 ± 0	0.23 ± 0	0.18 ± 0	0.2 ± 0	0.14 ± 0
	Q_1_	0.26	0.20	0.22	0.26	0.22	0.17	0.19	0.13
	Q_2_ (IQR)	0.26 (0.02) ^a^	0.20 (0.02) ^b^	0.23 (0.01) ^c^	0.27 (0.02) ^a^	0.23 (0.01) ^c^	0.18 (0.01) ^d^	0.20 (0.01) ^e^	0.14 (0.02) ^f^
	Q_3_	0.27	0.22	0.23	0.28	0.23	0.18	0.20	0.15
	$\overset{-}{x}$ ± SE	0.24 ± 0				0.17 ± 0			
	Q_1_	0.22				0.15			
	Q_2_ (IQR)	0.23 (0.04) ^a^				0.18 (0.05) ^b^			
	Q_3_	0.26				0.20			
Sucrose (SUC) content in nectar (*w*/*v*) (%)	$\overset{-}{x}$ ± SE	0.40 ± 0	0.55 ± 0	0.50 ± 0	0.41 ± 0	0.50 ± 0	0.64 ± 0	0.60 ± 0	0.71 ± 0
	Q_1_	0.39	0.54	0.49	0.39	0.49	0.62	0.59	0.71
	Q_2_ (IQR)	0.41 (0.02) ^a^	0.55 (0.02) ^b^	0.50 (0.02) ^c^	0.41 (0.03) ^a^	0.51 (0.03) ^c^	0.64 (0.02) ^d^	0.60 (0.02) ^e^	0.72 (0.02) ^f^
	Q_3_	0.42	0.56	0.51	0.43	0.52	0.65	0.61	0.72
	$\overset{-}{x}$ ± SE	0.48 ± 0.01				0.65 ± 0.01			
	Q_1_	0.42				0.6			
	Q_2_ (IQR)	0.49 (0.10) ^a^				0.63 (0.10) ^b^			
	Q_3_	0.52				0.71			
FRU/GLU	$\overset{-}{x}$ ± SE	0.82 ± 0.01	0.87 ± 0.02	0.85 ± 0.01	0.86 ± 0.01	0.86 ± 0.01	0.98 ± 0.01	0.99 ± 0.01	0.98 ± 0.02
	Q_1_	0.78	0.82	0.81	0.82	0.81	0.92	0.94	0.91
	Q_2_ (IQR)	0.82 (0.06) ^a^	0.86 (0.10) ^b^	0.84 (0.06) ^ab^	0.86 (0.06) ^b^	0.84 (0.10) ^ab^	0.97 (0.09) ^c^	0.97 (0.09) ^c^	0.96 (0.10) ^c^
	Q_3_	0.84	0.92	0.87	0.89	0.91	1.01	1.03	1.01
	$\overset{-}{x}$ ± SE	0.85 ± 0.01				0.98 ± 0.01			
	Q_1_	0.81				0.92			
	Q_2_ (IQR)	0.84 (0.09) ^a^				0.97 (0.09) ^b^			
	Q_3_	0.90				1.02			
SUC/(GLU + FRU)	$\overset{-}{x}$ ± SE	0.68 ± 0.01	1.24 ± 0.02	1.01 ± 0.01	0.7 ± 0.01	1.02 ± 0.02	1.76 ± 0.04	1.50 ± 0.02	2.51 ± 0.03
	Q_1_	0.65	1.20	0.96	0.65	0.95	1.66	1.45	2.41
	Q_2_ (IQR)	0.69 (0.07) ^a^	1.25 (0.10) ^b^	1.01 (0.09) ^c^	0.68 (0.10) ^a^	1.02 (0.13) ^c^	1.75 (0.17) ^d^	1.49 (0.10) ^e^	2.52 (0.19) ^f^
	Q_3_	0.72	1.29	1.06	0.75	1.08	1.82	1.54	2.60
	$\overset{-}{x}$ ± SE	0.93 ± 0.02				1.91 ± 0.05			
	Q_1_	0.71				1.52			
	Q_2_ (IQR)	0.96 (0.36) ^a^				1.73 (0.89) ^b^			
	Q_3_	1.07				2.41			

**Table 4:** Amino acid concentration (µM) in *Epipactis helleborine* nectar. The number of classes represents the effect of amino acids on insect chemoreceptors: I, no effect; II, inhibition of chemoreceptors; III, stimulation of the salt cell; IV, the ability to stimulate the sugar cell. Data represent the mean (x) ± standard error (SE), lower quartile (Q_1_), median (Q_2_), upper quartile (Q_3_), interquartile range (IQR), and biological replicate (n). Different letters indicate statistically significant differences according to the pairwise Wilcoxon rank sum test with the Benjamini–Hochberg adjustment (*p* < 0.05).

Amino Acid	Class	Statistic	Population							
			aBIA	aCAR	aGON	aSOS	aSUR	nPOG1	nPOG2	nZAB
			n = 22	n = 22	n = 29	n = 28	n = 30	n = 28	n = 32	n = 30
**Proteogenic Amino Acids**										
Asp	I	$\overset{-}{x}$ ± SE	50.75 ± 0.24	101.14 ± 1.03	102.69 ± 0.92	51.51 ± 0.42	100.30 ± 0.42	59.45 ± 0.57	53.47 ± 0.32	31.23 ± 0.29
		Q_1_	49.86	98.19	98.28	50.1	99.03	57.61	52.13	29.95
		Q_2_ (IQR)	50.51 (1.81) ^a^	100.89 (5.59) ^b^	102.10 (8.32) ^b^	51.48 (3.05) ^a^	100.26 (2.84) ^b^	59.88 (3.74) ^c^	53.15 (2.82) ^d^	31.17 (2.22) ^e^
		Q_3_	51.66	103.78	106.6	53.14	101.86	61.35	54.95	32.17
Glu	I	$\overset{-}{x}$ ± SE	107.35 ± 1.03	181.36 ± 1.72	195.43 ± 1.37	127.14 ± 10	268.45 ± 1.79	218.83 ± 1.80	228.37 ± 2.52	139.61 ± 1.47
		Q_1_	103.96	177.31	192.18	124.31	260.67	212.44	219.23	132.60
		Q_2_ (IQR)	107.82 (6.02) ^a^	181.90 (8.05) ^b^	193.79 (6.04) ^c^	127.50 (5.65) ^d^	266.90 (9.65) ^e^	218.88 (13.40) ^f^	229.42 (16.43) ^g^	138.05 (13.45) ^h^
		Q_3_	109.98	185.36	198.22	129.96	270.31	225.84	235.66	146.05
Ala	I	$\overset{-}{x}$ ± SE	8.43 ± 0.06	8.49 ± 0.07	10.20 ± 0.05	10.73 ± 0.10	11.07 ± 0.07	10.01 ± 0.11	10.21 ± 0.06	8.21 ± 0.09
		Q_1_	8.25	8.26	10.03	10.34	10.85	9.72	9.99	7.87
		Q_2_ (IQR)	8.39 (0.31) ^a^	8.57 (0.45) ^a^	10.18 (0.28) ^b^	10.77 (0.70) ^c^	11.02 (0.55) ^d^	10.07 (0.57) ^b^	10.24 (0.40) ^b^	8.16 (0.61) ^e^
		Q_3_	8.57	8.71	10.31	11.04	11.40	10.29	10.39	8.48
Cys	I	$\overset{-}{x}$ ± SE	18.83 ± 0.14	27.25 ± 0.14	27.91 ± 0.19	15.94 ± 0.12	23.03 ± 0.09	15.50 ± 0.17	15.11 ± 0.07	14.28 ± 0.12
		Q_1_	18.45	26.83	27.71	15.56	22.73	14.93	14.99	13.93
		Q_2_ (IQR)	18.87 (0.93) ^a^	27.36 (0.89) ^b^	28.11 (0.80) ^c^	15.90 (0.70) ^d^	23.03 (0.70) ^e^	15.37 (0.93) ^f^	15.13 (0.35) ^f^	14.31 (0.76) ^g^
		Q_3_	19.38	27.72	28.50	16.26	23.43	15.86	15.33	14.70
Gly	I	$\overset{-}{x}$ ± SE	6.09 ± 0.14	6.88 ± 0.11	7.79 ± 0.08	4.73 ± 0.05	11.15 ± 0.10	6.83 ± 0.05	7.83 ± 0.05	4.10 ± 0.06
		Q_1_	5.54	6.48	7.49	4.60	10.56	6.64	7.65	3.86
		Q_2_ (IQR)	6.28 (1.10) ^a^	6.97 (0.81) ^b^	7.71 (0.65) ^c^	4.79 (0.32) ^d^	11.30 (0.94) ^e^	6.87 (0.38) ^b^	7.81 (0.37) ^c^	4.17 (0.53) ^f^
		Q_3_	6.64	7.29	8.14	4.92	11.5	7.02	8.02	4.39
Ser	I	$\overset{-}{x}$ ± SE	17.39 ± 0.17	16.35 ± 0.19	22.71 ± 0.20	17.86 ± 0.26	36.48 ± 0.74	22.71 ± 0.15	20.43 ± 0.11	17.29 ± 0.16
		Q_1_	16.87	15.74	22.54	17.01	36.73	22.20	19.99	16.87
		Q_2_ (IQR)	17.07 (0.83) ^a^	16.13 (1.10) ^b^	22.99 (0.86) ^c^	17.86 (1.39) ^a^	37.17 (1.10) ^d^	22.60 (0.75) ^c^	20.17 (0.64) ^e^	17.28 (1.13) ^a^
		Q_3_	17.71	16.83	23.40	18.39	37.83	22.95	20.63	18.01
Thr	I	$\overset{-}{x}$ ± SE	35.58 ± 0.19	14.96 ± 0.11	20.13 ± 0.23	28.91 ± 0.17	11.93 ± 0.17	33.87 ± 0.20	31.77 ± 0.32	10.91 ± 0.09
		Q_1_	34.96	14.6	19.51	28.31	11.29	33.21	30.30	10.43
		Q_2_ (IQR)	35.71 (1.21) ^a^	14.95 (0.65) ^b^	20.01 (1.38) ^c^	28.90 (1.35) ^d^	12.17 (1.42) ^e^	33.80 (1.41) ^f^	31.72 (3.17) ^g^	10.79 (0.77) ^h^
		Q_3_	36.17	15.24	20.89	29.67	12.71	34.62	33.47	11.21
Tyr	I	$\overset{-}{x}$ ± SE	1.68 ± 0.03	1.07 ± 0.01	1.87 ± 0.02	1.70 ± 0.02	2.80 ± 0.06	1.77 ± 0.03	1.07 ± 0.01	1.60 ± 0.03
		Q_1_	1.60	1.04	1.80	1.65	2.59	1.69	1.02	1.50
		Q_2_ (IQR)	1.66 (0.15) ^abc^	1.06 (0.05) ^d^	1.89 (0.14) ^e^	1.69 (0.09) ^a^	2.81 (0.41) ^f^	1.79 (0.16) ^b^	1.06 (0.09) ^d^	1.60 (0.21) ^c^
		Q_3_	1.75	1.08	1.94	1.74	3.01	1.85	1.11	1.71
Arg	II	$\overset{-}{x}$ ± SE	4.04 ± 0.03	2.05 ± 0.02	3.65 ± 0.05	4.67 ± 0.05	2.71 ± 0.04	1.43 ± 0.02	2.06 ± 0.05	2.25 ± 0.03
		Q_1_	3.94	2.04	3.48	4.50	2.54	1.33	1.88	2.16
		Q_2_ (IQR)	4.01 (0.18) ^a^	2.06 (0.06) ^b^	3.63 (0.35) ^c^	4.68 (0.36) ^d^	2.68 (0.26) ^e^	1.44 (0.16) ^f^	2.01 (0.30) ^b^	2.26 (0.15) ^g^
		Q_3_	4.12	2.09	3.83	4.85	2.80	1.49	2.18	2.31
Asn	II	$\overset{-}{x}$ ± SE	30.06 ± 0.19	52.25 ± 0.5	53.71 ± 0.58	25.96 ± 0.28	101.72 ± 0.82	158.63 ± 1.87	146.85 ± 1.79	29.5 ± 0.38
		Q_1_	29.34	50.43	51.81	24.68	98.90	152.03	137.70	28.42
		Q_2_ (IQR)	30.21 (1.17) ^a^	51.61 (3.20) ^b^	54.12 (3.98) ^b^	26.26 (2.60) ^c^	102.21 (4.93) ^d^	156.23 (11.7) ^e^	148.15 (15.84) ^f^	29.19 (1.83) ^g^
		Q_3_	30.51	53.63	55.79	27.28	103.83	163.73	153.54	30.25
Gln	II	$\overset{-}{x}$ ± SE	29.38 ± 0.32	43.87 ± 0.37	56.01 ± 0.43	25.97 ± 0.22	57.35 ± 0.39	37.46 ± 0.18	34.09 ± 0.13	17.09 ± 0.15
		Q_1_	28.35	42.24	55.76	25.23	56.91	36.72	33.97	16.51
		Q_2_ (IQR)	29.96 (2.19) ^a^	44.20 (3.03) ^b^	56.81 (1.55) ^c^	25.99 (1.41) ^d^	57.53 (1.28) ^e^	37.58 (1.54) ^f^	34.34 (0.62) ^g^	17.24 (1.18) ^h^
		Q_3_	30.54	45.27	57.31	26.63	58.19	38.26	34.59	17.69
His	II	$\overset{-}{x}$ ± SE	4.42 ± 0.06	4.07 ± 0.05	5.31 ± 0.09	5.22 ± 0.08	6.77 ± 0.06	7.86 ± 0.08	6.93 ± 0.03	6.76 ± 0.07
		Q_1_	4.27	3.91	5.19	4.98	6.42	7.66	6.82	6.50
		Q_2_ (IQR)	4.42 (0.28) ^a^	4.01 (0.37) ^b^	5.38 (0.40) ^c^	5.27 (0.62) ^c^	6.76 (0.64) ^de^	7.88 (0.44) ^f^	6.92 (0.23) ^d^	6.70 (0.48) ^e^
		Q_3_	4.55	4.28	5.59	5.6	7.07	8.10	7.05	6.98
Lys	II	$\overset{-}{x}$ ± SE	5.38 ± 0.07	9.45 ± 0.13	10.36 ± 0.06	4.23 ± 0.17	11.99 ± 0.10	15.55 ± 0.17	15.39 ± 0.07	4.99 ± 0.05
		Q_1_	5.11	9.08	10.18	4.13	11.77	14.88	15.15	4.77
		Q_2_ (IQR)	5.40 (0.49) ^a^	9.43 (0.61) ^b^	10.35 (0.42) ^c^	4.31 (0.47) ^d^	12.02 (0.54) ^e^	15.30 (1.20) ^f^	15.35 (0.34) ^f^	5.01 (0.44) ^g^
		Q_3_	5.60	9.69	10.60	4.60	12.31	16.08	15.49	5.21
Pro	III	$\overset{-}{x}$ ± SE	12.45 ± 0.05	13.11 ± 0.10	14.09 ± 0.06	11.68 ± 0.11	7.65 ± 0.04	11.93 ± 0.16	11.11 ± 0.05	6.47 ± 0.04
		Q_1_	12.22	12.62	13.85	11.64	7.44	11.65	10.92	6.33
		Q_2_ (IQR)	12.50 (0.43) ^a^	13.25 (0.87) ^b^	14.15 (0.53) ^c^	11.79 (0.28) ^d^	7.63 (0.40) ^e^	12.14 (0.83) ^f^	11.04 (0.37) ^g^	6.45 (0.29) ^h^
		Q_3_	12.65	13.49	14.38	11.93	7.84	12.48	11.30	6.62
Ile	IV	$\overset{-}{x}$ ± SE	6.10 ± 0.09	15.01 ± 0.07	16.03 ± 0.08	5.45 ± 0.07	16.60 ± 0.11	15.71 ± 0.13	10.67 ± 0.07	14.42 ± 0.11
		Q_1_	5.76	14.84	15.81	5.25	16.20	15.18	10.35	14.03
		Q_2_ (IQR)	6.01 (0.67) ^a^	14.96 (0.41) ^b^	16.02 (0.59) ^c^	5.55 (0.47) ^d^	16.68 (0.80) ^e^	15.75 (0.99) ^c^	10.51 (0.69) ^f^	14.30 (0.67) ^g^
		Q_3_	6.44	15.25	16.40	5.72	17.01	16.16	11.04	14.70
Leu	IV	$\overset{-}{x}$ ± SE	5.37 ± 0.06	5.17 ± 0.04	7.39 ± 0.05	4.51 ± 0.04	6.91 ± 0.07	10.43 ± 0.08	8.07 ± 0.05	12.88 ± 0.45
		Q_1_	5.18	5.01	7.21	4.38	6.65	10.13	7.86	13.01
		Q_2_ (IQR)	5.37 (0.37) ^a^	5.13 (0.28) ^b^	7.31 (0.34) ^c^	4.50 (0.25) ^d^	6.95 (0.53) ^e^	10.39 (0.50) ^f^	8.03 (0.43) ^g^	13.30 (0.56) ^h^
		Q_3_	5.55	5.29	7.55	4.63	7.18	10.63	8.28	13.57
Met	IV	$\overset{-}{x}$ ± SE		4.53 ± 0.06	5.54 ± 0.05	1.36 ± 0.03	5.38 ± 0.08	4.89 ± 0.05	4.58 ± 0.04	1.63 ± 0.02
		Q_1_		4.29	5.38	1.23	5.04	4.72	4.41	1.56
		Q_2_ (IQR)		4.54 (0.46) ^a^	5.60 (0.33) ^b^	1.34 (0.25) ^c^	5.48 (0.66) ^b^	4.89 (0.30) ^d^	4.54 (0.33) ^a^	1.63 (0.13) ^e^
		Q_3_		4.75	5.71	1.48	5.71	5.02	4.74	1.69
Phe	IV	$\overset{-}{x}$ ± SE	14.12 ± 0.09	13.16 ± 0.09	13.27 ± 0.06	13.40 ± 0.09	22.42 ± 0.08	13.74 ± 0.14	12.06 ± 0.07	8.33 ± 0.04
		Q_1_	13.93	12.86	13.02	13.01	22.20	13.41	11.76	8.18
		Q_2_ (IQR)	14.18 (0.43) ^a^	13.16 (0.54) ^b^	13.31 (0.49) ^b^	13.41 (0.69) ^b^	22.48 (0.49) ^c^	13.89 (0.80) ^d^	12.03 (0.56) ^e^	8.32 (0.35) ^f^
		Q_3_	14.36	13.40	13.51	13.70	22.68	14.21	12.32	8.52
Trp	IV	$\overset{-}{x}$ ± SE	17.80 ± 0.13	17.87 ± 0.11	18.57 ± 0.09	15.12 ± 0.13	26.93 ± 0.12	23.71 ± 0.11	15.41 ± 0.10	10.56 ± 0.04
		Q_1_	17.32	17.52	18.27	14.71	26.54	23.52	15.01	10.44
		Q_2_ (IQR)	17.76 (0.99) ^a^	17.87 (0.68) ^a^	18.59 (0.54) ^b^	15.23 (0.92) ^c^	26.83 (0.75) ^d^	23.81 (0.52) ^e^	15.31 (0.71) ^c^	10.54 (0.30) ^f^
		Q_3_	18.31	18.2	18.81	15.62	27.29	24.04	15.72	10.74
Val	IV	$\overset{-}{x}$ ± SE	8.16 ± 0.08	12.64 ± 0.05	13.66 ± 0.09	8.37 ± 0.08	13.68 ± 0.15	8.53 ± 0.08	11.07 ± 0.06	6.97 ± 0.05
		Q_1_	7.89	12.51	13.31	8.01	13.17	8.17	10.89	6.82
		Q_2_ (IQR)	8.12 (0.43) ^a^	12.64 (0.28) ^b^	13.61 (0.77) ^c^	8.34 (0.60) ^ad^	13.78 (1.03) ^c^	8.60 (0.70) ^d^	11.05 (0.30) ^e^	6.92 (0.29) ^f^
		Q_3_	8.32	12.79	14.08	8.61	14.20	8.87	11.18	7.11
Sum of proteogenic AAs		$\overset{-}{x}$ ± SE	383.37 ± 2.00	550.7 ± 2.30	606.33 ± 1.79	384.46 ± 1.25	745.34 ± 2.11	678.83 ± 2.58	646.55 ± 3.06	349.07 ± 1.71
		Q_1_	376.03	546.30	600.62	380.76	736.59	674.65	634.03	343.99
		Q_2_ (IQR)	383.02 (14.80) ^a^	550.78 (9.60) ^b^	605.78 (13.21) ^c^	383.9 (8.95) ^a^	743.07 (16.38) ^d^	680.97 (12.11) ^e^	643.10 (23.07) ^f^	348.38 (10.15) ^g^
		Q_3_	390.83	555.9	613.83	389.71	752.97	686.76	657.09	354.14
**Non-proteogenic amino acids**										
Orn		$\overset{-}{x}$ ± SE	7.46 ± 0.08	7.59 ± 0.06	7.38 ± 0.06	6.52 ± 0.06	8.24 ± 0.09	5.47 ± 0.05	4.77 ± 0.03	4.61 ± 0.04
		Q_1_	7.30	7.32	7.17	6.40	7.89	5.30	4.64	4.44
		Q_2_ (IQR)	7.56 (0.42) ^ab^	7.65 (0.54) ^a^	7.41 (0.53) ^b^	6.59 (0.28) ^c^	8.23 (0.63) ^d^	5.43 (0.38) ^e^	4.72 (0.24) ^f^	4.62 (0.36) ^g^
		Q_3_	7.72	7.85	7.69	6.69	8.52	5.69	4.88	4.80
Cit		$\overset{-}{x}$ ± SE	2.62 ± 0.03			2.10 ± 0.03		3.11 ± 0.03	2.45 ± 0.04	5.46 ± 0.04
		Q_1_	2.53			2.01		3.01	2.31	5.30
		Q_2_ (IQR)	2.61 (0.19) ^a^			2.13 (0.18) ^b^		3.07 (0.22) ^c^	2.39 (0.30) ^d^	5.44 (0.21) ^e^
		Q_3_	2.72			2.19		3.22	2.61	5.51
Tau		$\overset{-}{x}$ ± SE	2.88 ± 0.04	3.45 ± 0.04	2.67 ± 0.03	3.24 ± 0.06	6.46 ± 0.04	3.42 ± 0.07	3.93 ± 0.03	6.53 ± 0.07
		Q_1_	2.76	3.32	2.56	3.01	6.27	3.16	3.82	6.32
		Q_2_ (IQR)	2.86 (0.22) ^a^	3.40 (0.26) ^b^	2.65 (0.17) ^c^	3.24 (0.35) ^d^	6.42 (0.35) ^e^	3.40 (0.54) ^b^	3.95 (0.20) ^f^	6.52 (0.45) ^e^
		Q_3_	2.98	3.59	2.73	3.35	6.62	3.70	4.02	6.77
AABA		$\overset{-}{x}$ ± SE	2.27 ± 0.11	1.74 ± 0.03	1.62 ± 0.03	2.89 ± 0.04	1.78 ± 0.07	0.85 ± 0.01	1.13 ± 0.01	1.27 ± 0.02
		Q_1_	2.28	1.63	1.57	2.82	1.71	0.82	1.08	1.19
		Q_2_ (IQR)	2.37 (0.15) ^a^	1.73 (0.21) ^b^	1.63 (0.17) ^c^	2.89 (0.20) ^d^	1.89 (0.23) ^e^	0.85 (0.07) ^f^	1.15 (0.10) ^g^	1.29 (0.16) ^h^
		Q_3_	2.43	1.85	1.74	3.02	1.94	0.89	1.18	1.35
BABA		$\overset{-}{x}$ ± SE	1.51 ± 0.07	3.67 ± 0.03	3.36 ± 0.03	1.04 ± 0.01	3.23 ± 0.02	1.06 ± 0.01	0.95 ± 0.05	2.42 ± 0.03
		Q_1_	1.52	3.56	3.29	1.01	3.14	1.03	0.99	2.30
		Q_2_ (IQR)	1.57 (0.13) ^a^	3.67 (0.22) ^b^	3.39 (0.21) ^c^	1.03 (0.08) ^de^	3.25 (0.17) ^f^	1.04 (0.08) ^d^	1.03 (0.06) ^e^	2.42 (0.25) ^g^
		Q_3_	1.65	3.78	3.50	1.08	3.30	1.11	1.06	2.56
GABA		$\overset{-}{x}$ ± SE	1.13 ± 0.02	1.03 ± 0.01	1.27 ± 0.02	1.08 ± 0.01	1.42 ± 0.02			
		Q_1_	1.04	1.01	1.21	1.04	1.33			
		Q_2_ (IQR)	1.12 (0.17) ^a^	1.03 (0.03) ^b^	1.26 (0.12) ^c^	1.09 (0.08) ^a^	1.41 (0.16) ^d^			
		Q_3_	1.22	1.04	1.33	1.12	1.49			
β-Ala		$\overset{-}{x}$ ± SE	1.05 ± 0.01	1.16 ± 0.02	1.50 ± 0.02	1.22 ± 0.02	1.78 ± 0.01		0.56 ± 0.04	0.60 ± 0.03
		Q_1_	1.02	1.09	1.46	1.13	1.74		0.55	0.60
		Q_2_ (IQR)	1.05 (0.07) ^a^	1.19 (0.12) ^b^	1.52 (0.13) ^c^	1.24 (0.15) ^b^	1.78 (0.08) ^d^		0.64 (0.12) ^e^	0.62 (0.10) ^e^
		Q_3_	1.09	1.21	1.59	1.29	1.82		0.67	0.70
Sum of non-proteogenic AAs		$\overset{-}{x}$ ± SE	18.92 ± 0.14	18.64 ± 0.09	17.79 ± 0.11	18.08 ± 0.11	22.91 ± 0.12	13.92 ± 0.10	13.80 ± 0.10	20.89 ± 0.13
		Q1	18.65	18.37	17.72	17.62	22.63	13.54	13.58	20.68
		Q2 (IQR)	19.03 (0.68) ^a^	18.75 (0.56) ^b^	17.82 (0.48) ^c^	18.16 (0.80) ^c^	22.86 (0.66) ^d^	13.99 (0.75) ^e^	13.86 (0.61) ^e^	20.85 (0.72) ^f^
		Q3	19.32	18.93	18.19	18.41	23.28	14.29	14.18	21.40

AABA, α-aminobutyric acid; Ala, L-alanine; Arg, L-arginine; Asn, L-asparagine; Asp, L-aspartic acid; β-Ala, β-alanine; BABA, β-aminobutyric acid; Cit, L-citrulline; Cys, L-cystine; GABA, γ-aminobutyric acid; Gln, L-glutamine; Glu, L-glutamic acid; Gly, glycine; His, L-histidine; Ile, L-isoleucine; Leu, L-leucine; Lys, L-lysine; Met, L-methionine; Orn, L-ornithine; Phe, L-phenylalanine; Pro, L-proline; Ser, L-serine; Tau, taurine; Thr, L-threonine; Trp, L-tryptophan; Tyr, L-tyrosine; Val, L-valine.

**Table 5:** Spatial variation in pollinaria removal (PR) and female reproductive success (FRS) in the natural and anthropogenic populations of *Epipactis helleborine.* Data represent the mean (x) ± standard error (SE), lower quartile (Q_1_), median (Q_2_), upper quartile (Q_3_), interquartile range (IQR), and biological replicate (n). Different letters indicate statistically significant differences according to the pairwise Wilcoxon rank sum test with the Benjamini–Hochberg adjustment (*p* < 0.05).

Parameter	Statistic	Population							
		aBIA	aCAR	aGON	aSOS	aSUR	nPOG1	nPOG2	nZAB
PR (%)	$\overset{-}{x}$ ± SE	88.40 ± 3.40	92.33 ± 2.48	92.58 ± 2.65	81.95 ± 4.06	83.60 ± 3.34	79.9 ± 4.01	74.33 ± 6.77	96.74 ± 1.50
	Q_1_	81.66	83.33	92	75.31	78.89	70	50	100
	Q_2_ (IQR)	94.23 (18.34) ^ab^	100 (16.67) ^ab^	100 (8) ^ab^	88.46 (24.69) ^a^	90 (17.48) ^a^	80 (30) ^a^	87.50 (50) ^a^	100 (0) ^b^
	Q_3_	100	100	100	100	96.37	100	100	100
	n	19	21	26	27	28	27	29	29
FRS (%)	$\overset{-}{x}$ ± SE	98.10 ± 1.24	83.95 ± 6.63	82.71 ± 5.98	94.11 ± 2.29	85.36 ± 4.17	62.48 ± 7.52	62.86 ± 10.72	87.48 ± 3.62
	Q_1_	100	80.83	80	91.81	84	42.14	27.50	81.66
	Q_2_ (IQR)	100 (0) ^a^	100 (19.17) ^b^	100 (20) ^ab^	100 (8.19) ^ab^	92.86 (16) ^b^	65.15 (42.08) ^c^	66.66 (72.5) ^c^	100 (18.34) ^b^
	Q_3_	100	100	100	100	100	84.22	100	100
	n	19	21	26	27	28	26	29	29
PR/FRS	$\overset{-}{x}$ ± SE	0.91 ± 0.04	1.05 ± 0.11	1.40 ± 0.34	0.88 ± 0.04	1.05 ± 0.08	1.81 ± 0.39	1.73 ± 0.43	1.19 ± 0.09
	Q_1_	0.81	0.93	0.96	0.78	0.90	0.89	1	1
	Q_2_ (IQR)	0.96 (0.19) ^a^	1 (0.07) ^ab^	1 (0.15) ^ab^	1 (0.22) ^a^	0.96 (0.18) ^ab^	1.15 (1.27) ^ab^	1 (1.25) ^ab^	1 (0.16) ^b^
	Q_3_	1	1	1.11	1	1.07	2.17	2.25	1.16
	n	19	17	24	27	27	16	11	27

**Table 6:** Impact of floral display, flower structures, and nectar composition on the reproductive success of *Epipactis helleborine* populations.

Population	Floral Display	Flower Structure	Sugars	Amino Acids
aBIA	IL → PR (r_S_ = 0.46)	DS → FRS (r_S_ = −0.50)	Fructose → PR (r_S_ = 0.48)	Asp → PR (r_S_ = 0.54)
		LF → FRS (r_S_ = −0.53)	Sugars → PR (r_S_ = 0.68)	Gly → PR (r_S_ = 0.56)
		WI → FRS (r_S_ = −0.49)		Gln → PR (r_S_ = 0.46)
		WH → FRS (r_S_ = −0.59)		Trp → PR (r_S_ = 0.55)
		WE → FRS (r_S_ = −0.48)		Cit → PR (r_S_ = −0.49)
				BABA → FRS (r_S_ = 0.51)
aCAR				Pro → PR (r_S_ = 0.48)
				Phe → PR (r_S_ = 0.48)
				Orn → PR (r_S_ = 0.61)
				GABA → PR (r_S_ = 0.49)
				Tyr → FRS (r_S_ = −0.46)
aGON				Tau → PR (r_S_ = −0.43)
aSOS	SH → FRS (r_S_ = −0.46)			Cit → PR (r_S_ = −0.39)
	IL → FRS (r_S_ = −0.61)			Tau → PR (r_S_ = −0.42)
				Tau → FRS (r_S_ = −0.39)
aSUR	SH → FRS (r_S_ = 0.43)	WS → PR (r_S_ = 0.43)		
	IL → FRS (r_S_ = 0.38)			
nPOG1		WS → PR (r_S_ = 0.50)		Pro → PR (r_S_ = 0.38)
		WE → PR (r_S_ = 0.52)		Trp → PR (r_S_ = 0.45)
nPOG2		DP → FRS (r_S_ = −0.37)		Asp → PR (r_S_ = −0.42)
				Arg → PR (r_S_ = 0.38)
				Met → PR (r_S_ = −0.38)
				AABA → PR (r_S_ = 0.42)
				Met → FRS (r_S_ = −0.41)
nZAB		LI → PR (r_S_ = −0.41)	Sucrose → FRS (r_S_ = −0.37)	
		LH → PR (r_S_ = −0.48)		

**Table 7:** Characteristics of habitats with *Epipactis helleborine* populations.

Population	Geographical Coordinate	Habitat Characteristics
aBIA	53.50192 N 22.52210 E	On the verge of a gravel road across *Pinus sylvestris* forest. Only *Melampyrum pratense* (high abundance) flowers together with *Epipactis helleborine*.
aCAR	53.46111 N 22.65337 E	On the verge of an asphalt road in the vicinity of a multi-species meadow.
aGON	53.46719 N 22.67077 E	In a mixed forest on the verge of asphalt road. In close proximity to the railroad and a large patch of *Tanacetum vulgare*.
aSOS	53.489287 N 22.583053 E	In the ditch on the verge of gravel road to the village of Sośnia with few apiaries, across the *Pinus sylvestris* forest. In the ground layer: *Thymus* sp., *Ranunculus acris*, *Achillea millefolium*, *Centaurea jacea*, *Jasione montana*, and *Anthemis tinctoria*.
aSUR	52.97390 N 22.98206 E	At the verge of an asphalt road and the *Cucurbita pepo* field. Under single trees along the road. In the undergrowth, mainly grasses. In the vicinity, there are agriculture fields with balks between them.
nPOG1	53.3040 N 22.5404 E	The border of alder forests and peat bogs with the domination of grasses and sedges, and partly under shrubs and trees.
nPOG2	53.3025385 N 22.5407106 E	In *Tilio carpinetum typicum* surrounded by peat bogs. Lack of co-flowering plants.
nZAB	53.29861 N 22.58101 E	Mineral island among peat bogs. Mainly open area with a small fragment of *Tilio Carpinetum typicum*. Neighboring mineral island with multispecies communities.

## Data Availability

Data are contained within the current article and Supplementary Material.

## Supplementary Materials

The following are available online at https://www.mdpi.com/article/10.3390/ijms24054276/s1.

## References

[1] Kull T., Hutchings M.J. (2006). A comparative analysis of decline in the distribution ranges of orchid species in Estonia and the United Kingdom. Biol. Conserv..

[2] Phillips R.D., Reiter N., Peakall R. (2020). Orchid conservation: From theory to practice. Ann. Bot..

[3] Bergman E., Ackerman J.D., Thompson J., Zimmerman J.K. (2006). Land-use history affects the distribution of the saprophytic orchid *Wullschlaegelia calcarata* in Puerto Rico’s Tabonuco Forest. Biotropica.

[4] Besi E.E., Nikong D., Mustafa M., Go R. (2019). Orchid diversity in anthropogenic-induced degraded tropical rainforest, an extrapolation towards conservation. Lankesteriana.

[5] Hundera K., Aerts R., Beenhouwer M.D., Overtveld K.V., Helsen K., Muys B., Honnay O. (2013). Both forest fragmentation and coffee cultivation negatively affect epiphytic orchid diversity in Ethiopian moist evergreen Afromontane forests. Biol. Conserv..

[6] Köster N., Friedrich K., Nieder J., Barthlott W. (2009). Conservation of epiphyte diversity in an Andean landscape transformed by human land use. Conserv. Biol..

[7] Fekete R., Löki V., Urgyán R., Süveges K., Lovas-Kiss Á., Vincze O., Molnár V.A. (2019). Roadside verges and cemeteries: Comparative analysis of anthropogenic orchid habitats in the Eastern Mediterranean. Ecol. Evol..

[8] Ackerman J. (2007). Invasive orchids: Weeds we hate to love?. Lankesteriana.

[9] Thomas C.D., Cameron A., Green R.E., Bakkenes M., Beaumont L.J., Collingham Y.C., Erasmus B.F.N., de Siqueira M.F., Grainger A., Hannah L. (2004). Extinction risk from climate change. Nature.

[10] Swarts N.D., Dixon K.W. (2009). Terrestrial orchid conservation in the age of extinction. Ann. Bot..

[11] Reiter N., Whitfield J., Pollard G., Bedggood W., Argall M., Dixon K., Davis B., Swarts N. (2016). Orchid re-introductions: An evaluation of success and ecological considerations using key comparative studies from Australia. Plant Ecol..

[12] Hinsley A., de Boer H.J., Fay M.F., Gale S.W., Gardiner L.M., Gunasekara R.S., Kumar P., Masters S., Metusala D., Roberts D.L. (2017). A review of the trade in orchids and its implications for conservation. Bot. J. Linn. Soc..

[13] Cariveau D.P., Winfree R. (2015). Causes of variation in wild bee responses to anthropogenic drivers. Curr. Opin. Insect. Sci..

[14] Goulson D., Lye G.C., Darvill B. (2008). Decline and conservation of bumble bees. Annu. Rev. Entomol..

[15] Goulson D., Nicholls E., Botías C., Rotheray E.L. (2015). Bee declines driven by combined stress from parasites, pesticides, and lack of flowers. Science.

[16] Potts S.G., Biesmeijer J.C., Kremen C., Neumann P., Schweiger O., Kunin W.E. (2010). Global pollinator declines: Trends, impacts and drivers. Trends Ecol. Evol..

[17] Adamowski W., Faliński J.B. (2002). Expansion of native orchids. Białowieża Geobotanical Station. Long Term Studies. Data Basis on the Vegetation and Environment 1952–2002.

[18] Adamowski W. (2006). Expansion of native orchids in anthropogenous habitats. Pol. Bot. Stud..

[19] Coates F., Lunt I.D., Tremblay R.L. (2006). Effects of disturbance on population dynamics of the threatened orchid *Prasophyllum correctum* D.L. Jones and implications for grassland management in south-eastern Australia. Biol. Conserv..

[20] Jacquemyn H., Brys R., Hutchings M.J. (2014). Biological flora of the British Isles: *Epipactis palustris*. J. Ecol..

[21] Jermakowicz E., Brzosko E. (2016). Demographic responses of boreal-montane orchid *Malaxis monophyllos* (L.) Sw. populations to contrasting environmental conditions. Acta Soc. Bot. Pol..

[22] Rewicz A., Jaskuła R., Rewicz T., Tończyk G. (2017). Pollinator diversity and reproductive success of *Epipactis helleborine* (L.) Crantz (Orchidaceae) in anthropogenic and natural habitats. PeerJ.

[23] Vakhrameeva M.G., Tatarenko I.V., Varlygina T.I., Torosyan G.K., Zagulski M.N. (2008). Orchids of Russia and Adjacent Countries (within the Borders of the Former USSR).

[24] Kolanowska M. (2013). Niche conservatism and the future potential range of *Epipactis helleborine* (Orchidaceae). PLoS ONE.

[25] Tałałaj I., Brzosko E. (2008). Selfing potential in *Epipactis palustris*, *E. helleborine* and *E. atrorubens* (Orchidaceae). Plant Syst. Evol..

[26] Kowalkowska A.K., Pawłowicz M., Guzanek P., Krawczyńska A.T. (2018). Floral nectary and osmophore of *Epipactis helleborine* (L.) Crantz (Orchidaceae). Protoplasma.

[27] Jakubska-Busse A., Kadej M., Prządo D., Steininger M. (2005). Pollination ecology of *Epipactis helleborine* (L.) Crantz (Orchidaceae, Neottieae) in the south-western Poland. Acta Bot. Sil..

[28] Ehlers B.K., Olesen J.M., Ågren J. (2002). Floral morphology and reproductive success in the orchid *Epipactis helleborine*: Regional and local across-habitat variation. Plant Syst. Evol..

[29] Jakubska-Busse A., Kadej M. (2011). The pollination of *Epipactis* Zinn, 1757 (Orchidaceae) species in Central Europe—The significance of chemical attractants, floral morphology and concomitant insects. Acta Soc. Bot. Pol..

[30] Brzosko E., Bajguz A., Burzyńska J., Chmur M. (2021). Nectar chemistry or flower morphology—What is more important for the reproductive success of generalist orchid *Epipactis palustris* in natural and anthropogenic populations?. Int. J. Mol. Sci..

[31] Brzosko E., Bajguz A., Chmur M., Burzyńska J., Jermakowicz E., Mirski P., Zieliński P. (2021). How are the flower structure and nectar composition of the generalistic orchid *Neottia ovata* adapted to a wide range of pollinators?. Int. J. Mol. Sci..

[32] Gijbels P., Ceulemans T., Van den Ende W., Honnay O. (2015). Experimental fertilization increases amino acid content in floral nectar, fruit set and degree of selfing in the orchid *Gymnadenia conopsea*. Oecologia.

[33] Li T., Wu S., Yang W., Selosse M.-A., Gao J. (2021). How mycorrhizal associations influence orchid distribution and population dynamics. Front. Plant Sci..

[34] Selosse M.-A. (2014). The latest news from biological interactions in orchids: In love, head to toe. New Phytol..

[35] Moré M., Amorim F.W., Benitez-Vieyra S., Medina A.M., Sazima M., Cocucci A.A. (2012). Armament imbalances: Match and mismatch in plant-pollinator traits of highly specialized long-spurred orchids. PLoS ONE.

[36] Trunschke J., Sletvold N., Ågren J. (2019). The independent and combined effects of floral traits distinguishing two pollination ecotypes of a moth-pollinated orchid. Ecol. Evol..

[37] Trunschke J., Sletvold N., Ågren J. (2020). Manipulation of trait expression and pollination regime reveals the adaptive significance of spur length. Evolution.

[38] Boberg E., Ågren J. (2009). Despite their apparent integration, spur length but not perianth size affects reproductive success in the moth-pollinated orchid *Platanthera bifolia*. Funct. Ecol..

[39] Boberg E., Alexandersson R., Jonsson M., Maad J., Ågren J., Nilsson L.A. (2014). Pollinator shifts and the evolution of spur length in the moth-pollinated orchid *Platanthera bifolia*. Ann. Bot..

[40] Little K.J., Dieringer G., Romano M. (2005). Pollination ecology, genetic diversity and selection on nectar spur length in *Platanthera lacera* (Orchidaceae). Plant Spec. Biol..

[41] Maad J., Alexandersson R. (2004). Variable selection in *Platanthera bifolia* (Orchidaceae): Phenotypic selection differed between sex functions in a drought year. J. Evol. Biol..

[42] Scopece G., Juillet N., Lexer C., Cozzolino S. (2017). Fluctuating selection across years and phenotypic variation in food-deceptive orchids. PeerJ.

[43] Sletvold N., Ågren J. (2011). Nonadditive effects of floral display and spur length on reproductive success in a deceptive orchid. Ecology.

[44] Sletvold N., Grindeland J.M., Ågren J. (2013). Vegetation context influences the strength and targets of pollinator-mediated selection in a deceptive orchid. Ecology.

[45] Sletvold N., Trunschke J., Smit M., Verbeek J., Ågren J. (2016). Strong pollinator-mediated selection for increased flower brightness and contrast in a deceptive orchid. Evolution.

[46] de Jager M.L., Peakall R. (2019). Experimental examination of pollinator-mediated selection in a sexually deceptive orchid. Ann. Bot..

[47] Caruso C.M., Eisen K.E., Martin R.A., Sletvold N. (2018). A meta-analysis of the agents of selection on floral traits. Evolution.

[48] Brzosko E., Mirski P. (2021). Floral nectar chemistry in orchids: A short review and meta-analysis. Plants.

[49] David T.I., Storkey J., Stevens C.J. (2019). Understanding how changing soil nitrogen affects plant–pollinator interactions. Arthropod-Plant Interact..

[50] Gardener M.C., Gillman M.P. (2002). The taste of nectar—A neglected area of pollination ecology. Oikos.

[51] Gijbels P., Van den Ende W., Honnay O. (2015). Phenotypic selection on nectar amino acid composition in the Lepidoptera pollinated orchid species *Gymnadenia conopsea*. Oikos.

[52] Baker H.G., Baker I. (1990). The predictive value of nectar chemistry to the recognition of pollinator types. Israel J. Bot..

[53] Abrahamczyk S., Kessler M., Hanley D., Karger D.N., Müller M.P.J., Knauer A.C., Keller F., Schwerdtfeger M., Humphreys A.M. (2017). Pollinator adaptation and the evolution of floral nectar sugar composition. J. Evol. Biol..

[54] Nicolson S.W., Thornburg R.W., Nicolson S.W., Nepi M., Pacini E. (2007). Nectar chemistry. Nectaries and Nectar.

[55] Petanidou T. (2005). Sugars in Mediterranean floral nectars: An ecological and evolutionary approach. J. Chem. Ecol..

[56] Roguz K., Hill L., Koethe S., Lunau K., Roguz A., Zych M. (2021). Visibility and attractiveness of *Fritillaria* (Liliaceae) flowers to potential pollinators. Sci. Rep..

[57] Vandelook F., Janssens S.B., Gijbels P., Fischer E., Van den Ende W., Honnay O., Abrahamczyk S. (2019). Nectar traits differ between pollination syndromes in Balsaminaceae. Ann. Bot..

[58] Willmer P., Willmer P. (2011). Pollination by butterflies and moths. Pollination and Floral Ecology.

[59] Nicolson S.W. (2022). Sweet solutions: Nectar chemistry and quality. Philos. Trans. R. Soc. Lond. B Biol. Sci..

[60] Pamminger T., Becker R., Himmelreich S., Schneider C.W., Bergtold M. (2019). The nectar report: Quantitative review of nectar sugar concentrations offered by bee visited flowers in agricultural and non-agricultural landscapes. PeerJ.

[61] Pyke G.H., Waser N.M. (1981). The production of dilute nectars by hummingbird and honeyeater flowers. Biotropica.

[62] Fowler R.E., Rotheray E.L., Goulson D. (2016). Floral abundance and resource quality influence pollinator choice. Insect Conserv. Divers..

[63] Parachnowitsch A.L., Manson J.S., Sletvold N. (2019). Evolutionary ecology of nectar. Ann. Bot..

[64] Brzosko E., Bajguz A. (2019). Nectar composition in moth-pollinated *Platanthera bifolia* and *P. chlorantha* and its importance for reproductive success. Planta.

[65] Nocentini D., Pacini E., Guarnieri M., Martelli D., Nepi M. (2013). Intrapopulation heterogeneity in floral nectar attributes and foraging insects of an ecotonal Mediterranean species. Plant Ecol..

[66] Petanidou T., Van Laere A., Ellis W.N., Smets E. (2006). What shapes amino acid and sugar composition in Mediterranean floral nectars?. Oikos.

[67] Heil M. (2011). Nectar: Generation, regulation and ecological functions. Trends Plant Sci..

[68] Ebeling A., Klein A.-M., Schumacher J., Weisser W.W., Tscharntke T. (2008). How does plant richness affect pollinator richness and temporal stability of flower visits?. Oikos.

[69] Ghazoul J. (2006). Floral diversity and the facilitation of pollination. J. Ecol..

[70] Juillet N., Gonzalez M.A., Page P.A., Gigord L.D.B. (2007). Pollination of the European food-deceptive *Traunsteinera globosa* (Orchidaceae): The importance of nectar-producing neighbouring plants. Plant Syst. Evol..

[71] Olesen J.M., Jordano P. (2002). Geographic patterns in plant-pollinator mutualistic networks. Ecology.

[72] Venjakob C., Leonhardt S., Klein A.-M. (2020). Inter-individual nectar chemistry changes of field Scabious, *Knautia arvensis*. Insects.

[73] Duffy K.J., Stout J.C. (2008). The effects of plant density and nectar reward on bee visitation to the endangered orchid *Spiranthes romanzoffiana*. Acta Oecol.-Int. J. Ecol..

[74] Lachmuth S., Henrichmann C., Horn J., Pagel J., Schurr F.M. (2017). Neighbourhood effects on plant reproduction: An experimental-analytical framework and its application to the invasive *Senecio inaequidens*. J. Ecol..

[75] Tremblay R.L., Ackerman J.D., Zimmerman J.K., Calvo R.N. (2005). Variation in sexual reproduction in orchids and its evolutionary consequences: A spasmodic journey to diversification. Biol. J. Linn. Soc..

[76] Jakubska-Busse A., Dorota P., Mieczysław S., Jadwiga A.-K., Kadej M. (2005). Why do pollinators become "sluggish"? Nectar chemical constituents from *Epipactis helleborine* (L.) Crantz (Orchidaceae). Appl. Ecol. Environ. Res..

[77] Neiland M.R.M., Wilcock C.C. (1998). Fruit set, nectar reward, and rarity in the Orchidaceae. Am. J. Bot..

[78] Tew N.E., Memmott J., Vaughan I.P., Bird S., Stone G.N., Potts S.G., Baldock K.C.R. (2021). Quantifying nectar production by flowering plants in urban and rural landscapes. J. Ecol..

[79] Kwak M.M., Jennersten O. (1991). Bumblebee visitation and seedset in *Melampyrum pratense* and *Viscaria vulgaris*: Heterospecific pollen and pollen limitation. Oecologia.

[80] Parra-Tabla V., Vargas C.F., Magaña-Rueda S., Navarro J. (2000). Female and male pollination success of *Oncidium ascendens* Lindey (Orchidaceae) in two contrasting habitat patches. Biol. Conserv..

[81] Pellegrino G., Bellusci F. (2014). Effects of human disturbance on reproductive success and population viability of *Serapias cordigera* (Orchidaceae). Bot. J. Linn. Soc..

[82] Grindeland J.M., Sletvold N., Ims R.A. (2005). Effects of floral display size and plant density on pollinator visitation rate in a natural population of *Digitalis purpurea*. Funct. Ecol..

[83] Kindlmann P., Jersáková J. (2006). Effect of floral display on reproductive success in terrestrial orchids. Folia Geobot..

[84] Maad J. (2000). Phenotypic selection in hawkmoth-pollinated *Platanthera bifolia*: Targets and fitness surfaces. Evolution.

[85] Sletvold N., Grindeland J.M., Ågren J. (2010). Pollinator-mediated selection on floral display, spur length and flowering phenology in the deceptive orchid *Dactylorhiza lapponica*. New Phytol..

[86] Vallius E., Arminen S., Salonen V. (2006). Are There Fitness Advantages Associated with a Large Inflorescence in *Gymnadenia conopsea* ssp. *conopsea*?. http://www.r-b-o.eu/rbo_public/Vallius_et_al_2006.html.

[87] Vafaee Y., Mohammadi G., Nazari F., Fatahi M., Kaki A., Gholami S., Ghorbani A., Khadivi A. (2021). Phenotypic characterization and seed-micromorphology diversity of the threatened terrestrial orchids: Implications for conservation. S. Afr. J. Bot..

[88] Rewicz A., Rewers M., Jędrzejczyk I., Rewicz T., Kołodziejek J., Jakubska-Busse A. (2018). Morphology and genome size of *Epipactis helleborine* (L.) Crantz (Orchidaceae) growing in anthropogenic and natural habitats. PeerJ.

[89] Armbruster W.S. (2017). The specialization continuum in pollination systems: Diversity of concepts and implications for ecology, evolution and conservation. Funct. Ecol..

[90] Alexandersson R., Johnson S.D. (2002). Pollinator-mediated selection on flower-tube length in a hawkmoth-pollinated *Gladiolus* (Iridaceae). Proc. Biol. Sci..

[91] Trunschke J., Sletvold N., Ågren J. (2017). Interaction intensity and pollinator-mediated selection. New Phytol..

[92] Jacquemyn H., Brys R. (2020). Lack of strong selection pressures maintains wide variation in floral traits in a food-deceptive orchid. Ann. Bot..

[93] Brys R., Jacquemyn H., Hermy M. (2008). Pollination efficiency and reproductive patterns in relation to local plant density, population size, and floral display in the rewarding *Listera ovata* (Orchidaceae). Bot. J. Linn. Soc..

[94] Roguz K., Bajguz A., Chmur M., Gołębiewska A., Roguz A., Zych M. (2019). Diversity of nectar amino acids in the *Fritillaria* (Liliaceae) genus: Ecological and evolutionary implications. Sci. Rep..

[95] Kim Y.S., Smith B.H. (2000). Effect of an amino acid on feeding preferences and learning behavior in the honey bee, *Apis mellifera*. J. Insect Physiol..

[96] Tie S., He Y.-D., Lázaro A., Inouye D.W., Guo Y.-H., Yang C.-F. (2022). Floral trait variation across individual plants within a population enhances defense capability to nectar robbing. Plant Divers..

[97] Zambon V., Agostini K., Nepi M., Rossi M.L., Martinelli A.P., Sazima M. (2020). Nectar as manipulator: How nectar traits influence changes in pollinator groups of *Aechmea vanhoutteana*, a bromeliad from the Brazilian Atlantic Forest. Bot. J. Linn. Soc..

[98] Peter C.I., Johnson S.D. (2009). Reproductive biology of *Acrolophia cochlearis* (Orchidaceae): Estimating rates of cross-pollination in epidendroid orchids. Ann. Bot..

[99] Wolff D. (2006). Nectar sugar composition and volumes of 47 species of gentianales from a Southern Ecuadorian Montane Forest. Ann. Bot..

[100] Rewicz A., Bomanowska A., Shevera M., Kurowski J., Krasoń K., Zielińska K. (2017). Cities and disturbed areas as man-made shelters for orchid communities. Not. Bot. Horti. Agrobot. Cluj-Napoca.

[101] Heil M. (2005). Postsecretory hydrolysis of nectar sucrose and specialization in ant/plant mutualism. Science.

[102] Levin E., McCue M.D., Davidowitz G. (2017). More than just sugar: Allocation of nectar amino acids and fatty acids in a Lepidopteran. Proc. Biol. Sci..

[103] Mevi-Schütz J., Erhardt A. (2005). Amino acids in nectar enhance butterfly fecundity: A long-awaited link. Am. Nat..

[104] Nepi M. (2014). Beyond nectar sweetness: The hidden ecological role of non-protein amino acids in nectar. J. Ecol..

[105] Pyke G.H. (2016). Plant-pollinator co-evolution: It’s time to reconnect with Optimal Foraging Theory and Evolutionarily Stable Strategies. Perspect. Plant Ecol. Evol. Syst..

[106] Carter C., Shafir S., Yehonatan L., Palmer R.G., Thornburg R. (2006). A novel role for proline in plant floral nectars. Naturwissenschaften.

[107] Nepi M., Soligo C., Nocentini D., Abate M., Guarnieri M., Cai G., Bini L., Puglia M., Bianchi L., Pacini E. (2012). Amino acids and protein profile in floral nectar: Much more than a simple reward. Flora.

[108] Felicioli A., Sagona S., Galloni M., Bortolotti L., Bogo G., Guarnieri M., Nepi M. (2018). Effects of nonprotein amino acids on survival and locomotion of *Osmia bicornis*. Insect Mol. Biol..

[109] Carlesso D., Smargiassi S., Pasquini E., Bertelli G., Baracchi D. (2021). Nectar non-protein amino acids (NPAAs) do not change nectar palatability but enhance learning and memory in honey bees. Sci. Rep..

[110] Park S., Thornburg R.W. (2009). Biochemistry of nectar proteins. J. Plant Biol..

[111] Burkle L.A., Irwin R.E. (2009). The effects of nutrient addition on floral characters and pollination in two subalpine plants, *Ipomopsis aggregata* and *Linum lewisii*. Plant Ecol..

[112] R Core Team R: A Language and Environment for Statistical Computing (R Version 4.2.2, Innocent and Trusting). R Foundation for Statistical Computing 2022. https://www.R-project.org/.

[113] Fox J., Weisberg S. (2011). An R Companion to Applied Regression.

[114] Hamilton N.E., Ferry M. (2018). ggtern: Ternary diagrams using ggplot2. J. Stat. Softw..

[115] Lê S., Josse J., Husson F. (2008). FactoMineR: An R package for multivariate analysis. J. Stat. Softw..

[116] Revelle W. (2022). Psych: Procedures for Personality and Psychological Research.

[117] Kassambara A., Mundt F. (2019). Factoextra: Extract and Visualize the Results of Multivariate data analyses (R Package Version 1.0.6). https://CRAN.R-project.org/package=factoextra.

