# Supplementary material for: Does Reproductive Success in Natural and Anthropogenic Populations of Generalist Epipactis helleborine Depend on Flower Morphology and Nectar Composition?
Source: Int J Mol Sci. 2023 Feb 21;24(5):4276. doi: 10.3390/ijms24054276 (PMC10001846; doi:10.3390/ijms24054276)
Supplement: Supplementary file 1 [file ijms-24-04276-s001.zip › ijms-2201102-supplementary.pdf]

International Journal of *Molecular Sciences*

Supplementary Materials

Does Reproductive Success in Natural and Anthropogenic Populations of Generalist *Epipactis helleborine* Depend on Flower Morphology and Nectar Composition?

Emilia Brzosko *, Andrzej Bajguz, Justyna Burzyńska, and Magdalena Chmur

[1] Department of Biology and Plant Ecology, Faculty of Biology, University of Bialystok, Ciolkowskiego 1J, 15-245 Bialystok, Poland

* Correspondence: emilka@uwb.edu.pl

Citation: To be added by editorial staff during production.

Academic Editor: First name Lastname

Received: date
Accepted: date
Published: date

Publisher's Note: MDPI stays neutral with regard to jurisdictional claims in published maps and institutional affiliations.

Copyright: © 2022 by the authors. Submitted for possible open access publication under the terms and conditions of the Creative Commons Attribution (CC BY) license (https://creativecommons.org/licenses/by/4.0/).

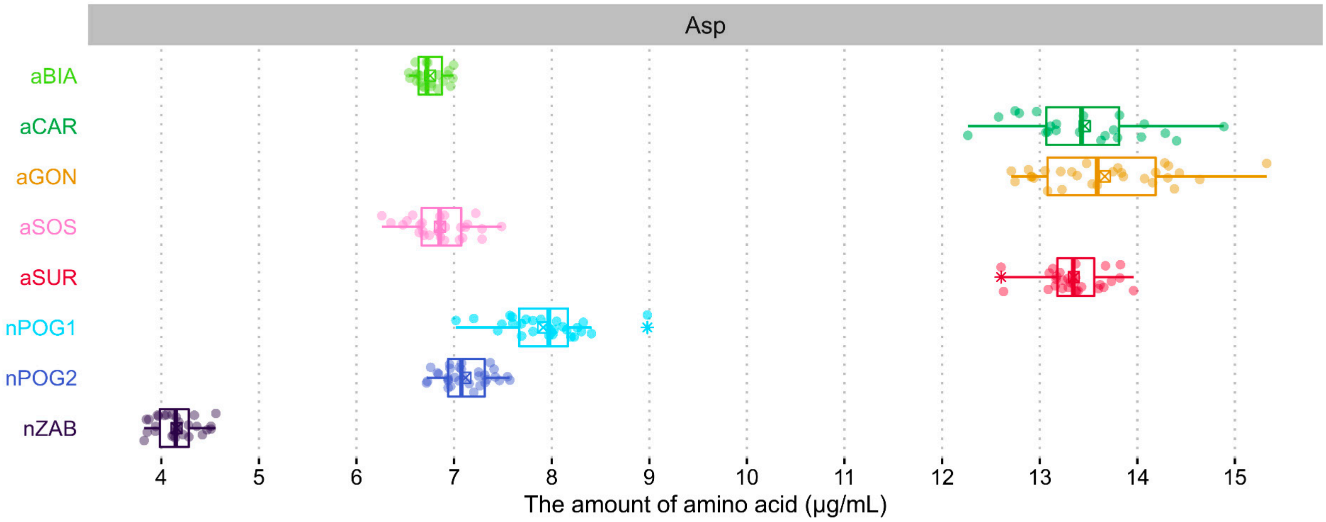

Figure S1. Boxplots of amino acids amounts for *Epipactis helleborine* natural (nPOG1, nPOG2, and nZAB) and anthropogenic (aBIA, aCAR, aGON, aSOS, and aSUR) populations. Colored dots are individual samples. The crossed square shows the mean. The lower and upper hinges correspond to the lower (Q_1) and upper (Q_3) quartiles. Thus box length shows the interquartile range (IQR). The thicker line inside boxes corresponds to the median. The lower whisker extends from the hinge to the smallest value at most Q_1 - 1.5 × IQR of the hinge. The upper whisker extends from the hinge to the largest value no further than Q_3 + 1.5 × IQR. Data beyond the end of the whiskers, indicated with an asterisk symbol, are outliers.

Continued on the next pages

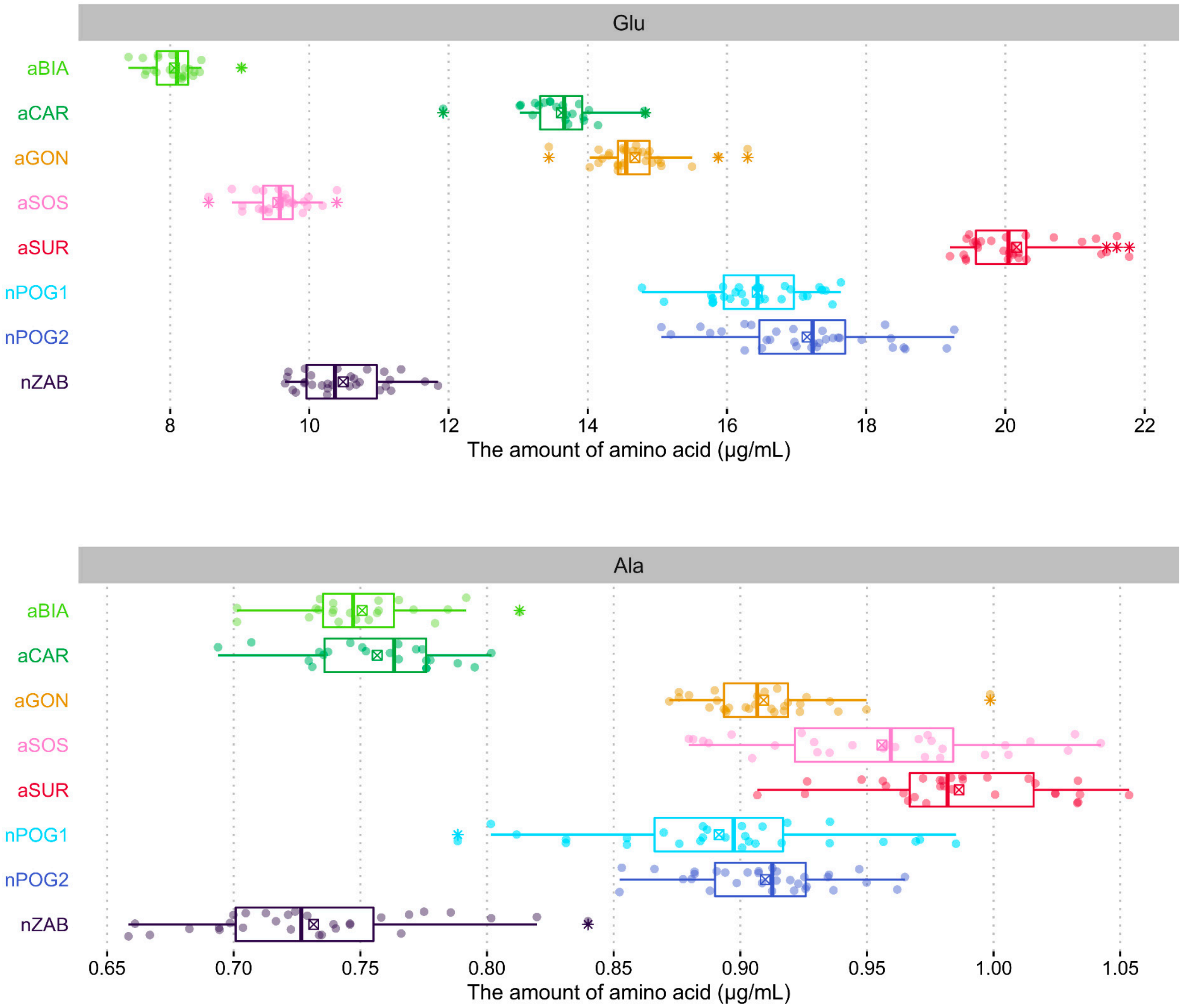
Glu
aBIA
aCAR
aGON
aSOS
aSUR
nPOG1
nPOG2
nZAB
8
10
12
14
16
18
20
22
The amount of amino acid (µg/mL)
Ala
aBIA
aCAR
aGON
aSOS
aSUR
nPOG1
nPOG2
nZAB
0.65
0.70
0.75
0.80
0.85
0.90
0.95
1.00
1.05
The amount of amino acid (µg/mL)

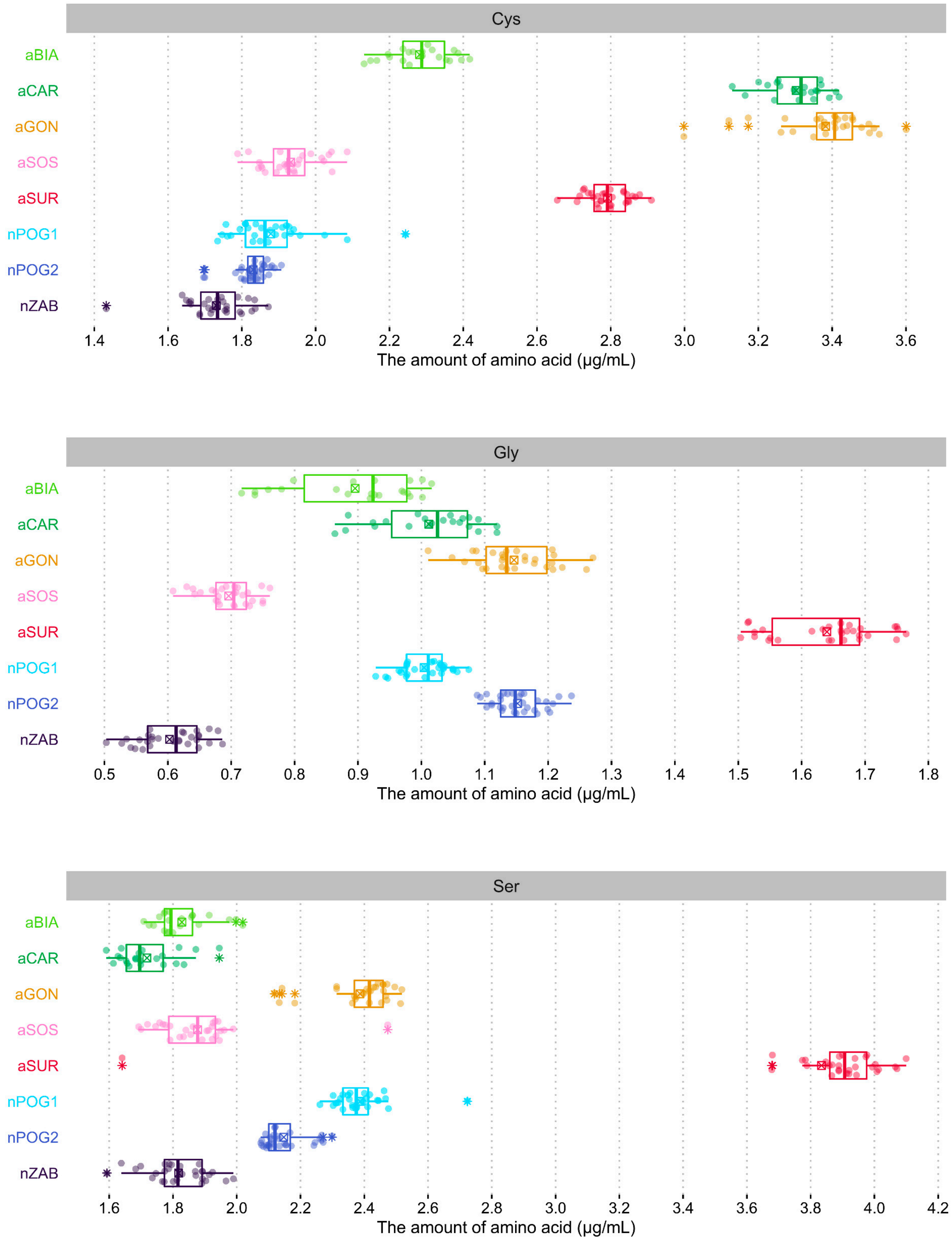
Cys
aBIA
aCAR
aGON
aSOS
aSUR
nPOG1
nPOG2
nZAB
1.4
1.6
1.8
2.0
2.2
2.4
2.6
2.8
3.0
3.2
3.4
3.6
The amount of amino acid (μg/mL)
Gly
0.5
0.6
0.7
0.8
0.9
1.0
1.1
1.2
1.3
1.4
1.5
1.6
1.7
1.8
The amount of amino acid (μg/mL)
Ser
1.6
1.8
2.0
2.2
2.4
2.6
2.8
3.0
3.2
3.4
3.6
3.8
4.0
4.2
The amount of amino acid (μg/mL)

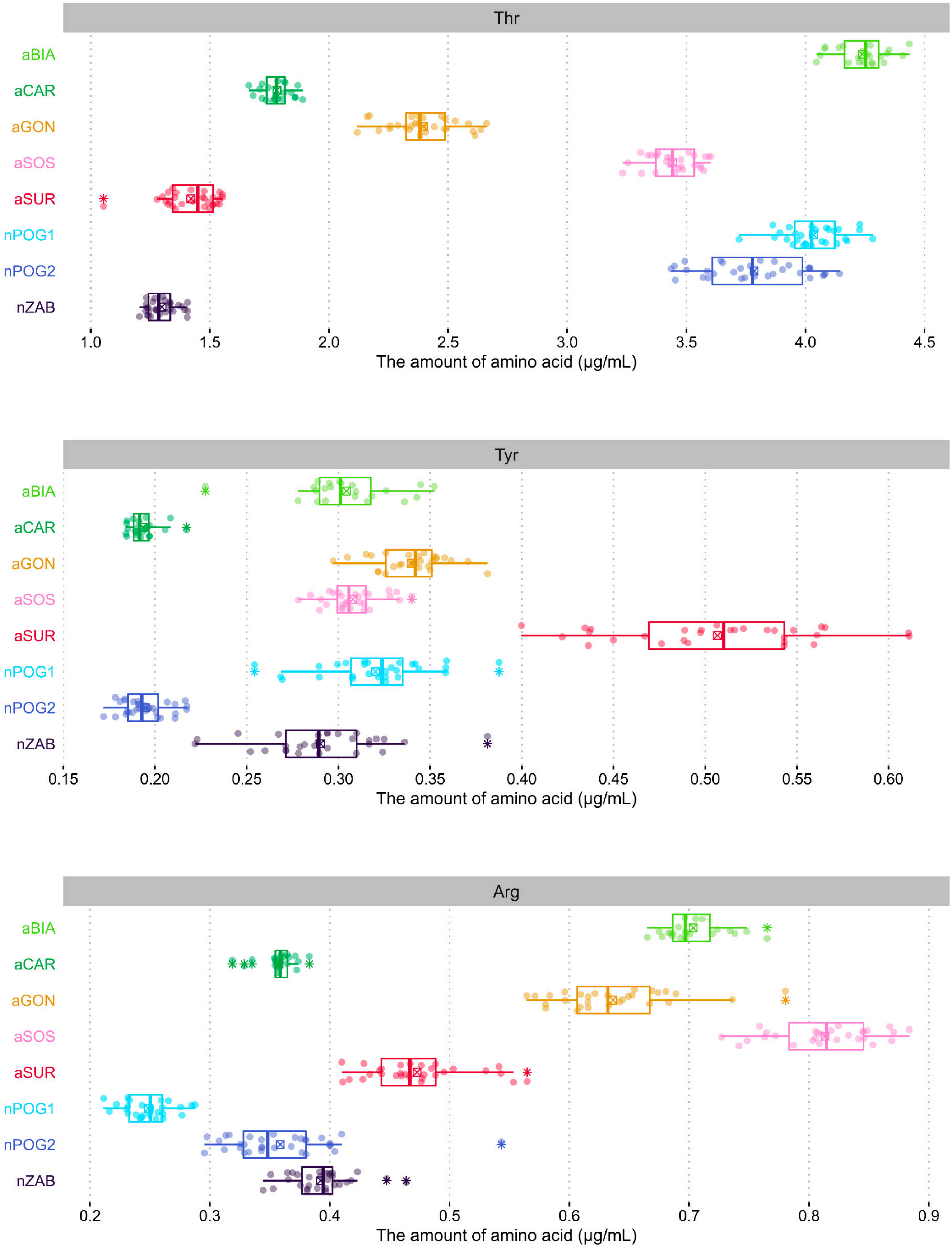
Thr
aBIA
aCAR
aGON
aSOS
aSUR
nPOG1
nPOG2
nZAB
1.0
1.5
2.0
2.5
3.0
3.5
4.0
4.5
The amount of amino acid (μg/mL)
Tyr
aBIA
aCAR
aGON
aSOS
aSUR
nPOG1
nPOG2
nZAB
0.15
0.20
0.25
0.30
0.35
0.40
0.45
0.50
0.55
0.60
The amount of amino acid (μg/mL)
Arg
aBIA
aCAR
aGON
aSOS
aSUR
nPOG1
nPOG2
nZAB
0.2
0.3
0.4
0.5
0.6
0.7
0.8
0.9
The amount of amino acid (μg/mL)

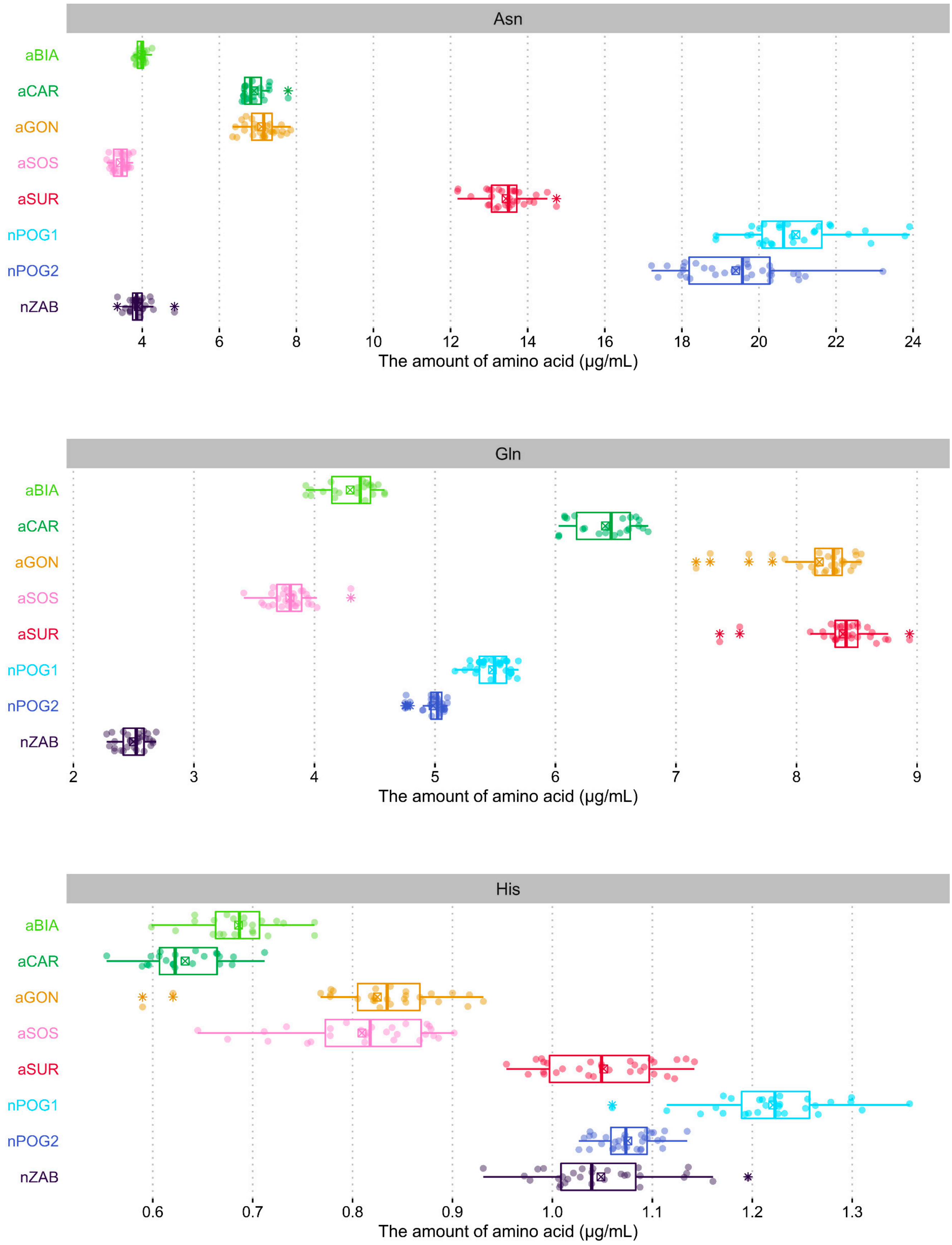
Asn
aBIA
aCAR
aGON
aSOS
aSUR
nPOG1
nPOG2
nZAB
4 6 8 10 12 14 16 18 20 22 24
The amount of amino acid (μg/mL)
Gln
aBIA
aCAR
aGON
aSOS
aSUR
nPOG1
nPOG2
nZAB
2 3 4 5 6 7 8 9
The amount of amino acid (μg/mL)
His
aBIA
aCAR
aGON
aSOS
aSUR
nPOG1
nPOG2
nZAB
0.6 0.7 0.8 0.9 1.0 1.1 1.2 1.3
The amount of amino acid (μg/mL)

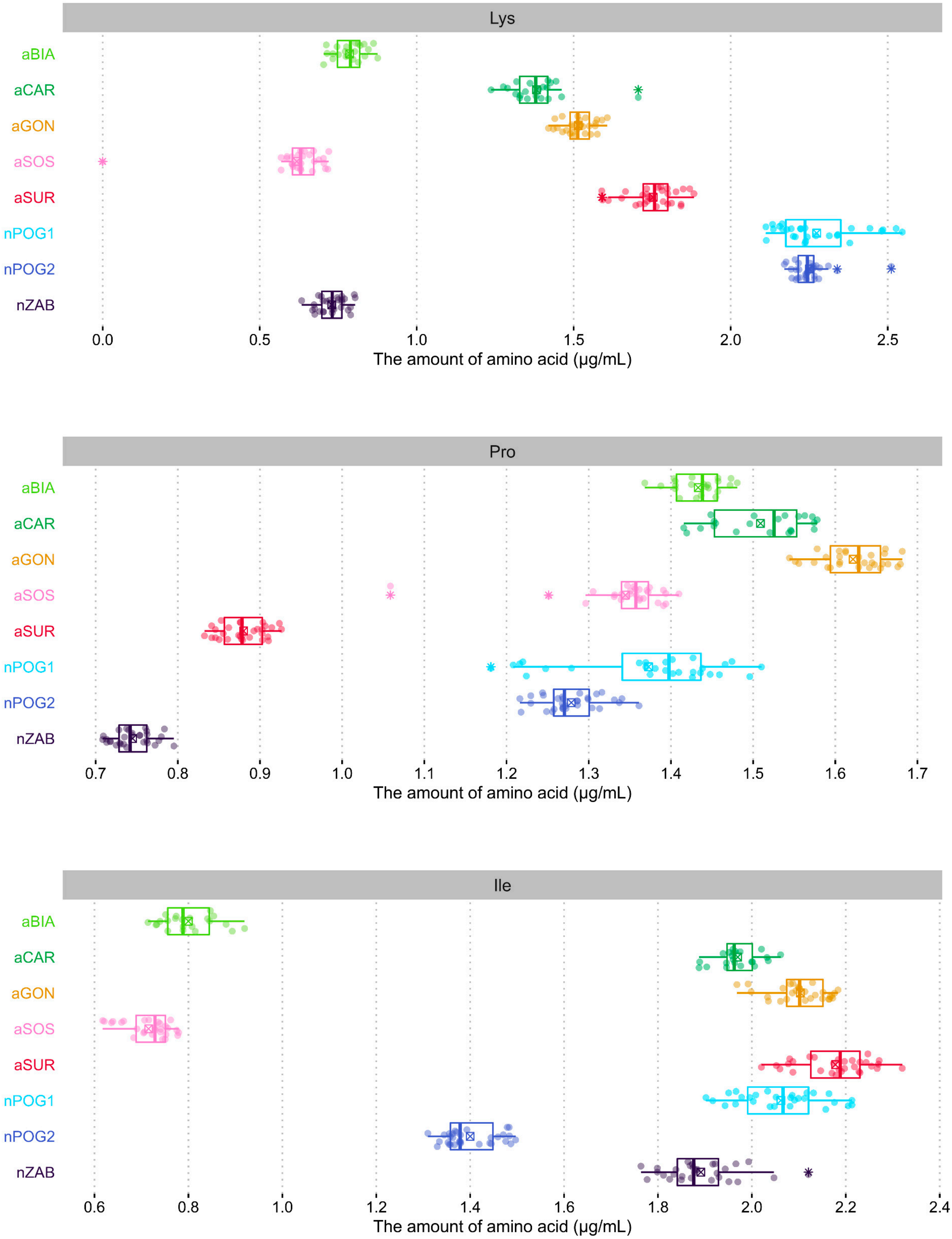
Lys
aBIA
aCAR
aGON
aSOS
aSUR
nPOG1
nPOG2
nZAB
0.0
0.5
1.0
1.5
2.0
2.5
The amount of amino acid (μg/mL)
Pro
aBIA
aCAR
aGON
aSOS
aSUR
nPOG1
nPOG2
nZAB
0.7
0.8
0.9
1.0
1.1
1.2
1.3
1.4
1.5
1.6
1.7
The amount of amino acid (μg/mL)
Ile
aBIA
aCAR
aGON
aSOS
aSUR
nPOG1
nPOG2
nZAB
0.6
0.8
1.0
1.2
1.4
1.6
1.8
2.0
2.2
2.4
The amount of amino acid (μg/mL)

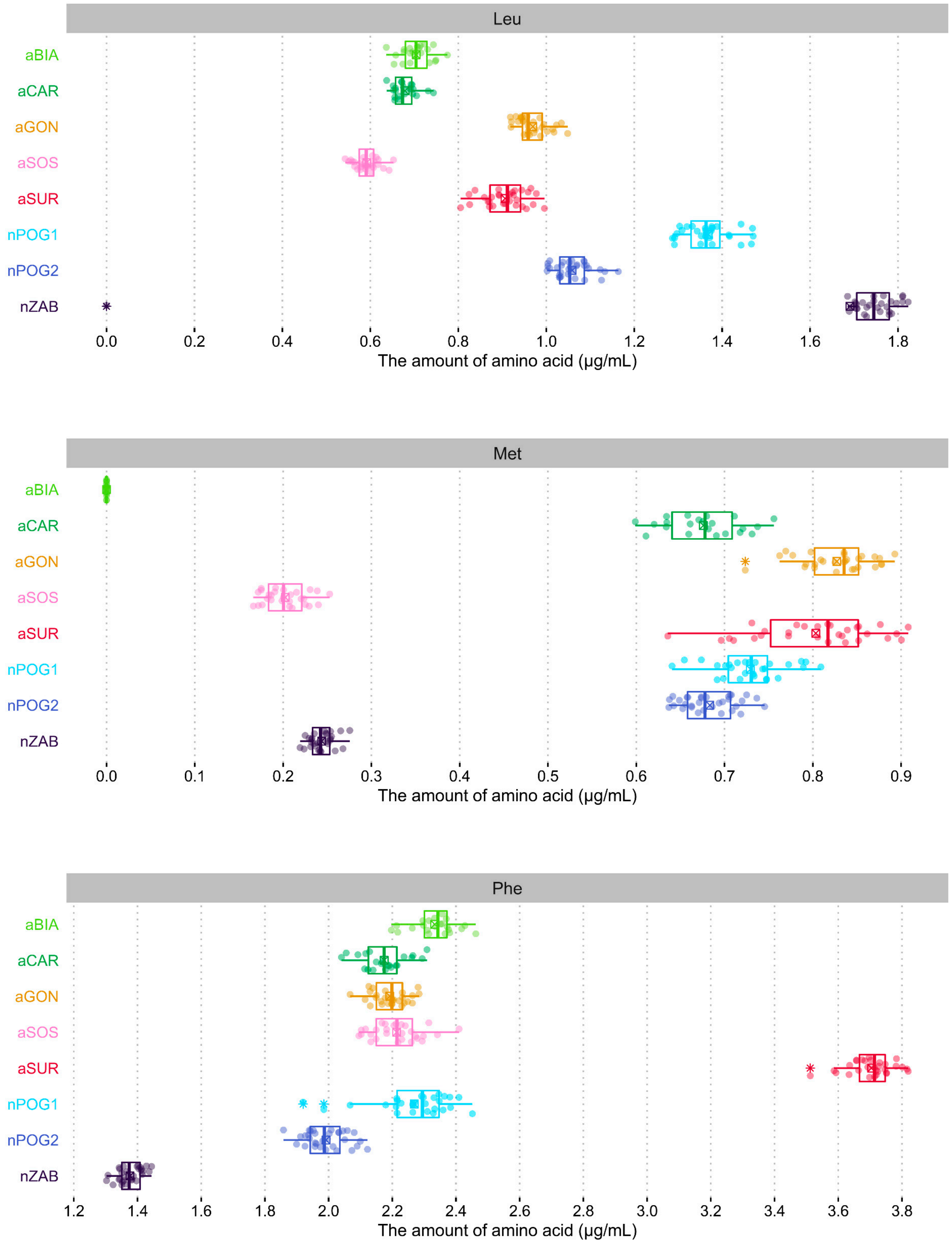
Leu
aBIA
aCAR
aGON
aSOS
aSUR
nPOG1
nPOG2
nZAB
0.0
0.2
0.4
0.6
0.8
1.0
1.2
1.4
1.6
1.8
The amount of amino acid (µg/mL)
Met
0.0
0.1
0.2
0.3
0.4
0.5
0.6
0.7
0.8
0.9
The amount of amino acid (µg/mL)
Phe
1.2
1.4
1.6
1.8
2.0
2.2
2.4
2.6
2.8
3.0
3.2
3.4
3.6
3.8
The amount of amino acid (µg/mL)

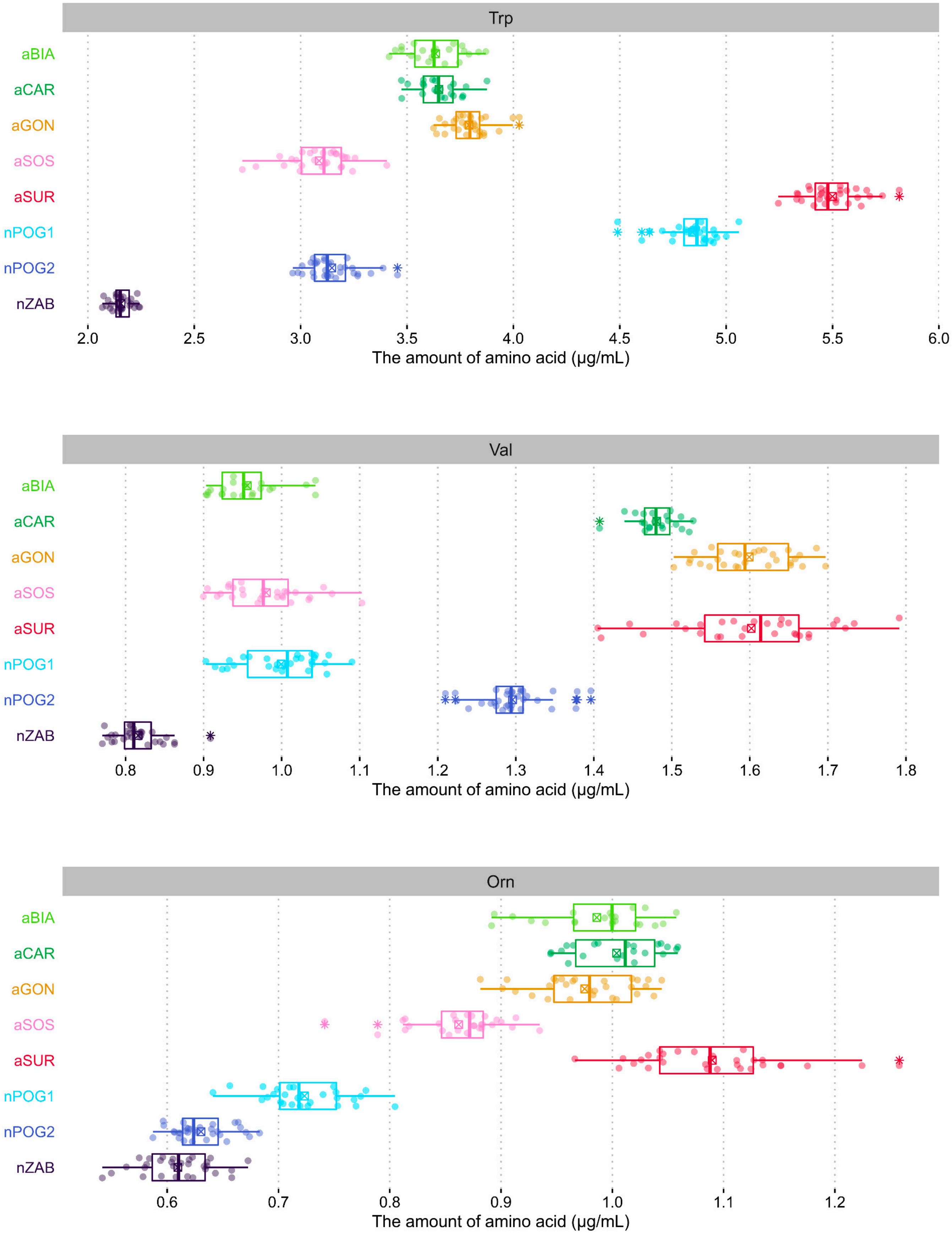
Trp
aBIA
aCAR
aGON
aSOS
aSUR
nPOG1
nPOG2
nZAB
2.0 2.5 3.0 3.5 4.0 4.5 5.0 5.5 6.0
The amount of amino acid (μg/mL)
Val
aBIA
aCAR
aGON
aSOS
aSUR
nPOG1
nPOG2
nZAB
0.8 0.9 1.0 1.1 1.2 1.3 1.4 1.5 1.6 1.7 1.8
The amount of amino acid (μg/mL)
Orn
aBIA
aCAR
aGON
aSOS
aSUR
nPOG1
nPOG2
nZAB
0.6 0.7 0.8 0.9 1.0 1.1 1.2
The amount of amino acid (μg/mL)

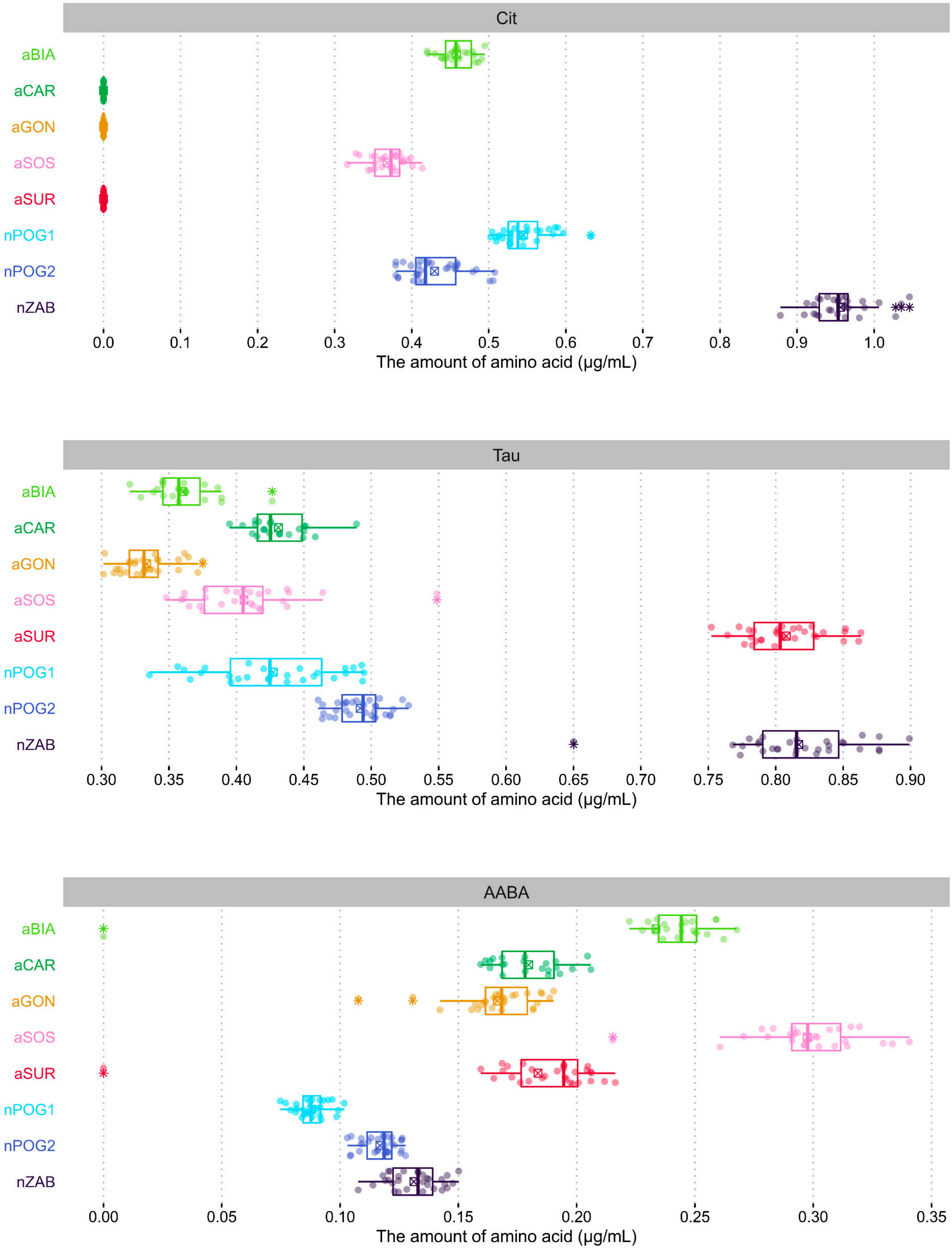
Cit
aBIA
aCAR
aGON
aSOS
aSUR
nPOG1
nPOG2
nZAB
0.0
0.1
0.2
0.3
0.4
0.5
0.6
0.7
0.8
0.9
1.0
The amount of amino acid (μg/mL)
Tau
aBIA
aCAR
aGON
aSOS
aSUR
nPOG1
nPOG2
nZAB
0.30
0.35
0.40
0.45
0.50
0.55
0.60
0.65
0.70
0.75
0.80
0.85
0.90
The amount of amino acid (μg/mL)
AABA
aBIA
aCAR
aGON
aSOS
aSUR
nPOG1
nPOG2
nZAB
0.00
0.05
0.10
0.15
0.20
0.25
0.30
0.35
The amount of amino acid (μg/mL)

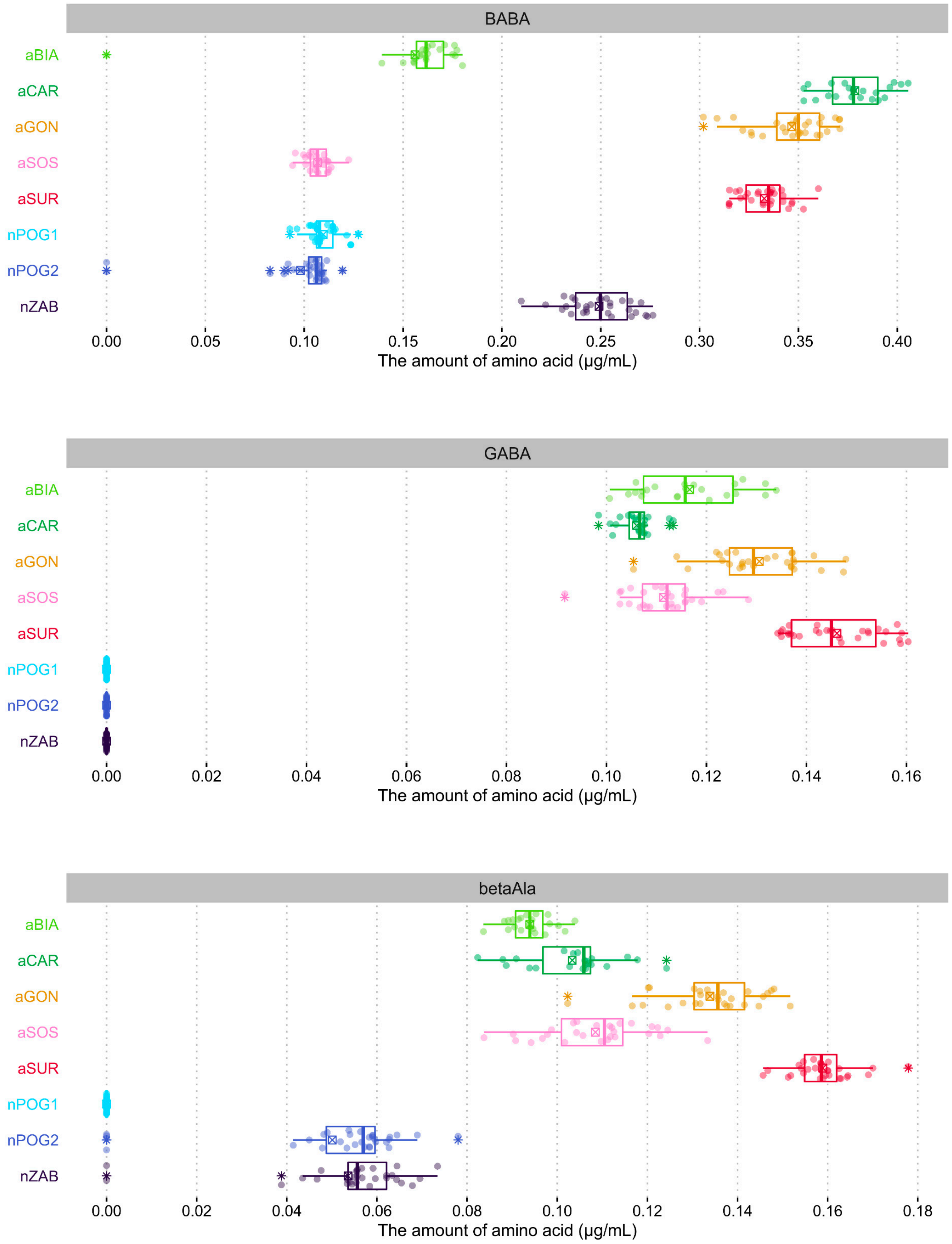
BABA
aBIA
aCAR
aGON
aSOS
aSUR
nPOG1
nPOG2
nZAB
0.00
0.05
0.10
0.15
0.20
0.25
0.30
0.35
0.40
The amount of amino acid (µg/mL)
GABA
aBIA
aCAR
aGON
aSOS
aSUR
nPOG1
nPOG2
nZAB
0.00
0.02
0.04
0.06
0.08
0.10
0.12
0.14
0.16
The amount of amino acid (µg/mL)
betaAla
aBIA
aCAR
aGON
aSOS
aSUR
nPOG1
nPOG2
nZAB
0.00
0.02
0.04
0.06
0.08
0.10
0.12
0.14
0.16
0.18
The amount of amino acid (µg/mL)

aBIA

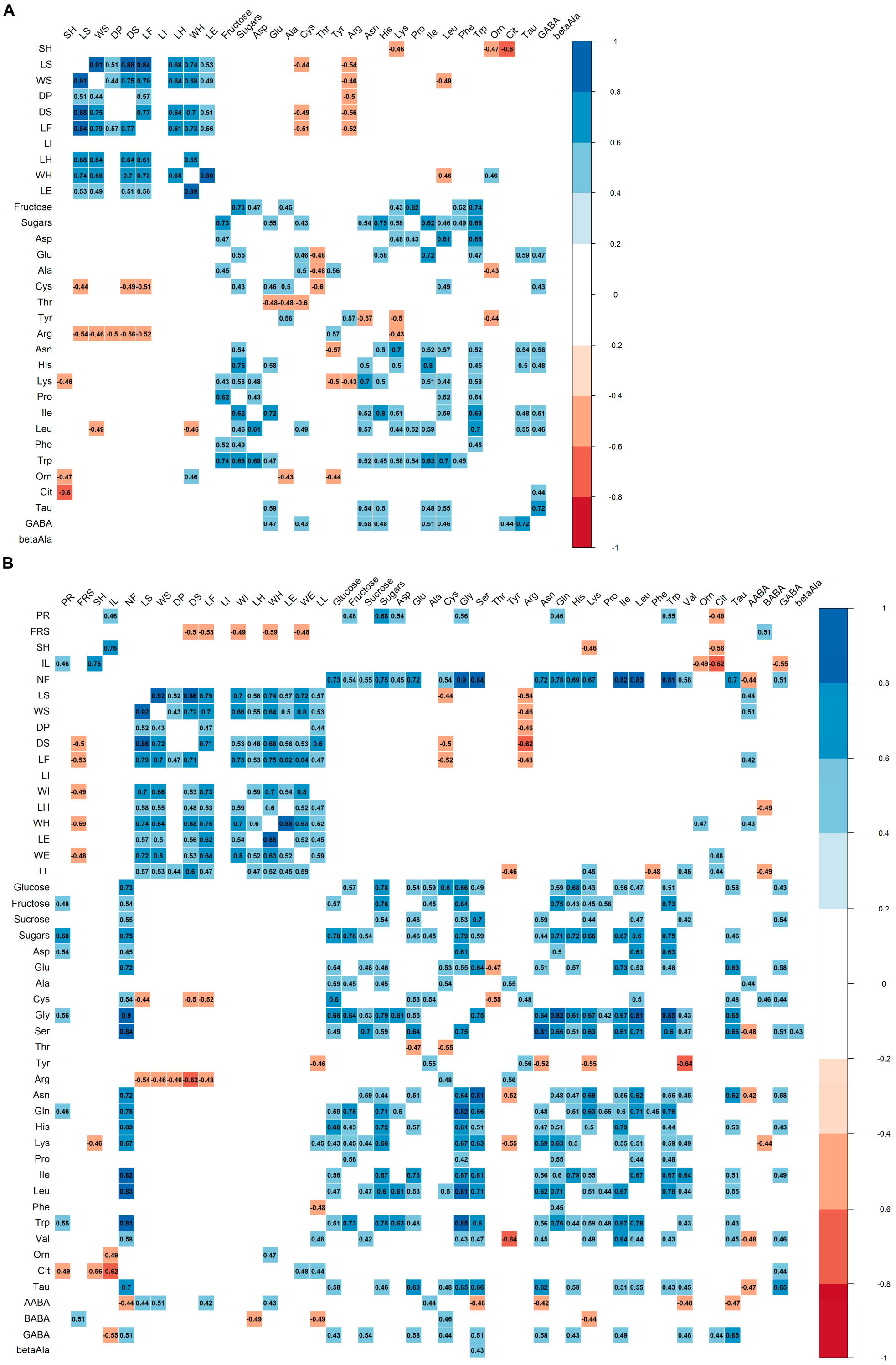

Figure S2. Correlation heatmap for reproductive success (that is, pollinaria removal, PR, and female reproductive success ,FRS), floral display (that is, SH, IL, and NF), flower structures (that is, LS, WS, DP, DS, LF, LI, WI, LH, WH, LE, WE, and LL), and nectar composition (that is, sugars and amino acids) of *Epipactis helleborine* populations. Significant positive correlations are colored blue, while negative correlations are colored red (p <0.05). Insignificant correlations are omitted. **A.** The colored and labeled scale codes show the value of (**A**) the Pearson rank correlation coefficient (r) and (**B**) the Spearman rank correlation coefficient (rs). The abbreviations are explained in Table 1.

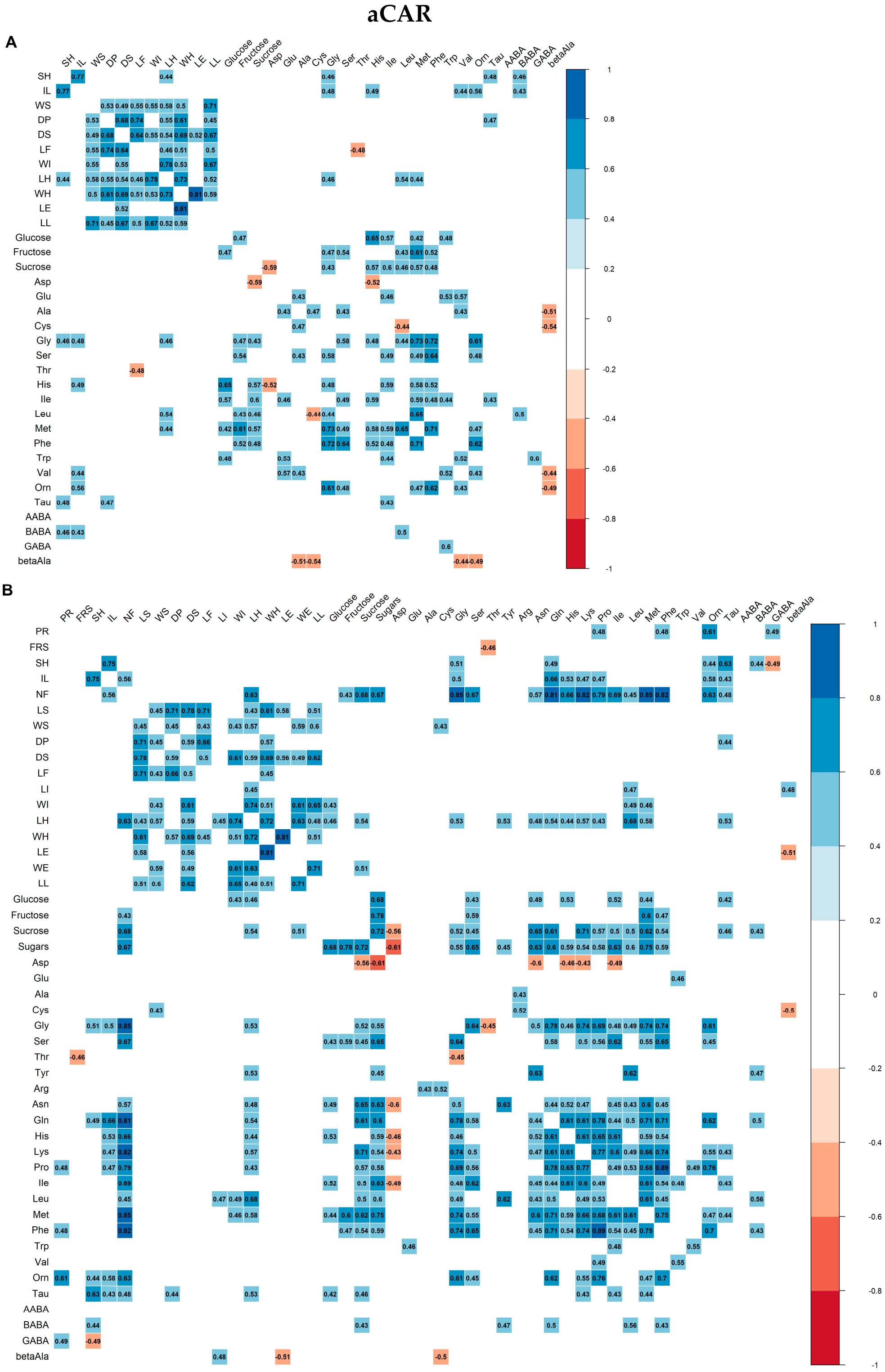

Figure S2. *Continued.*

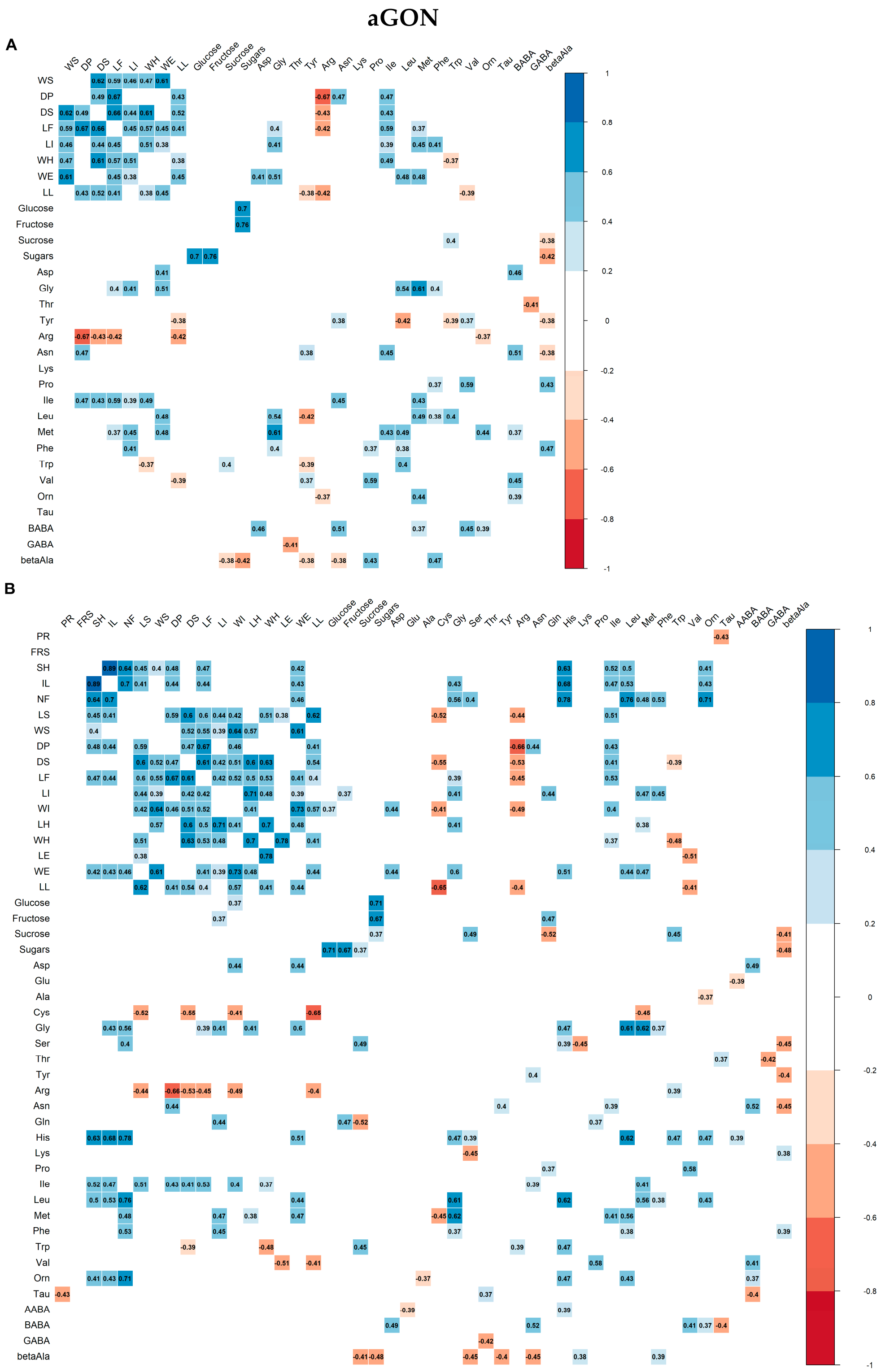

Figure S2. *Continued.*

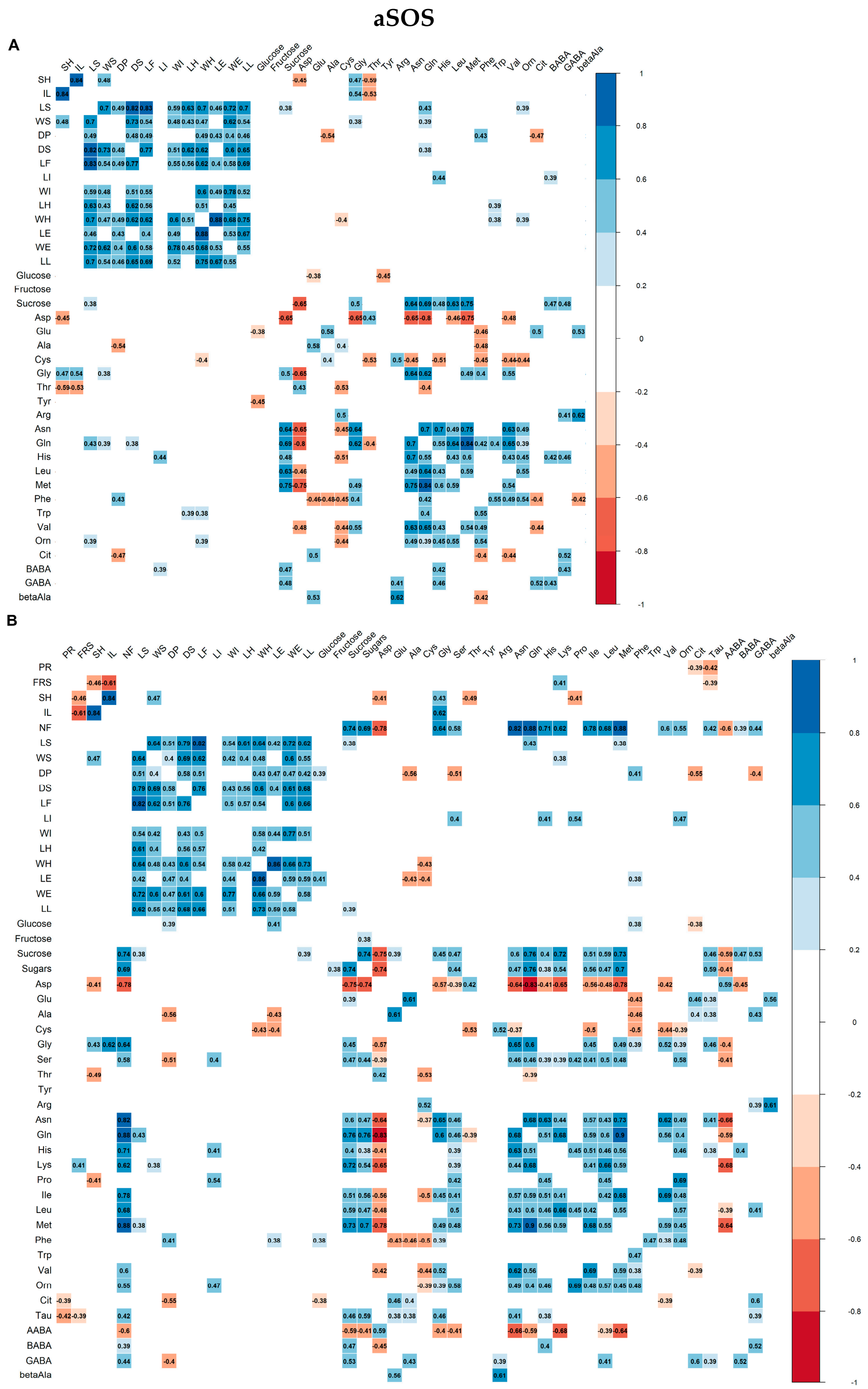

Figure S2. *Continued.*

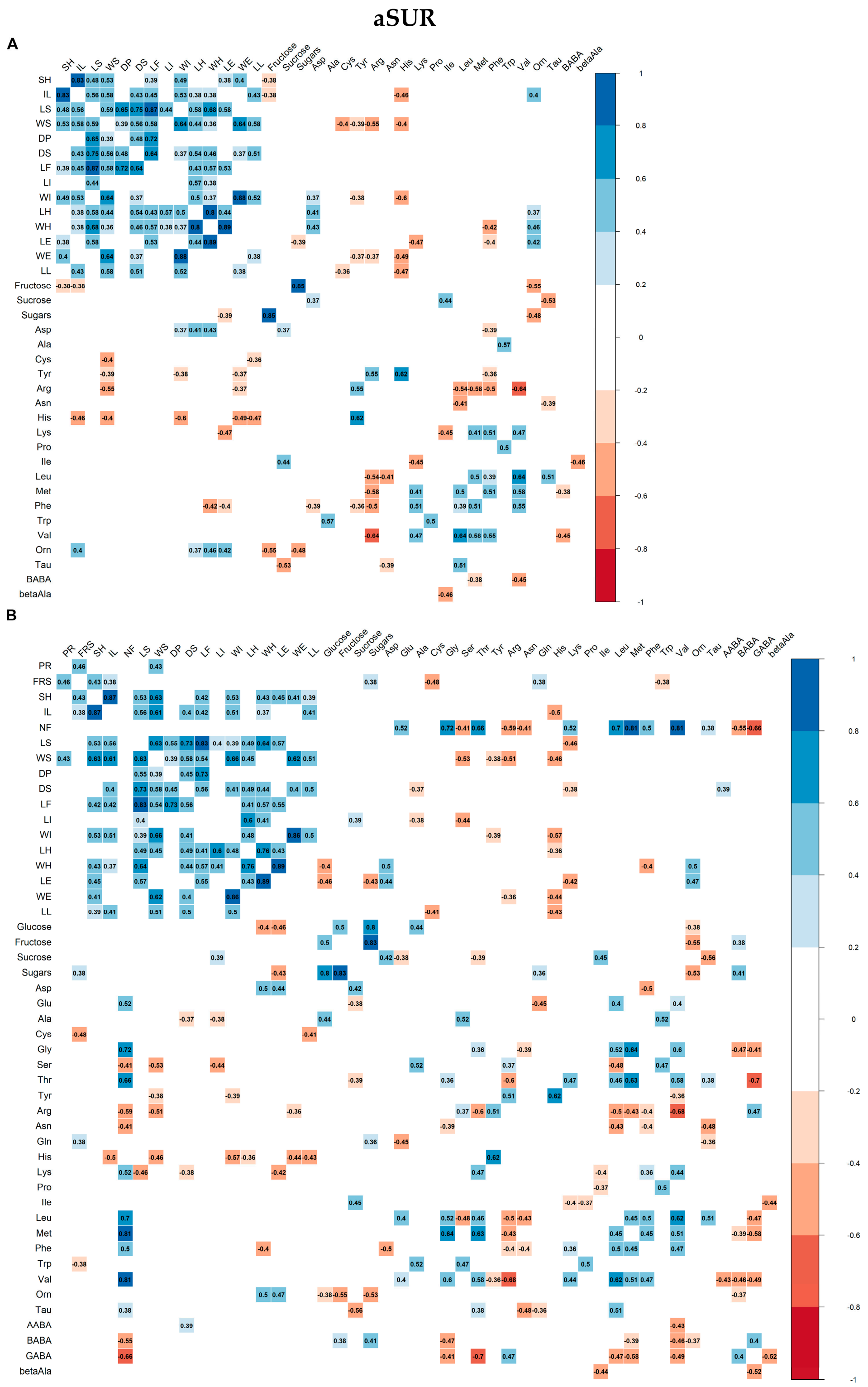

Figure S2. *Continued.*

nPOG1

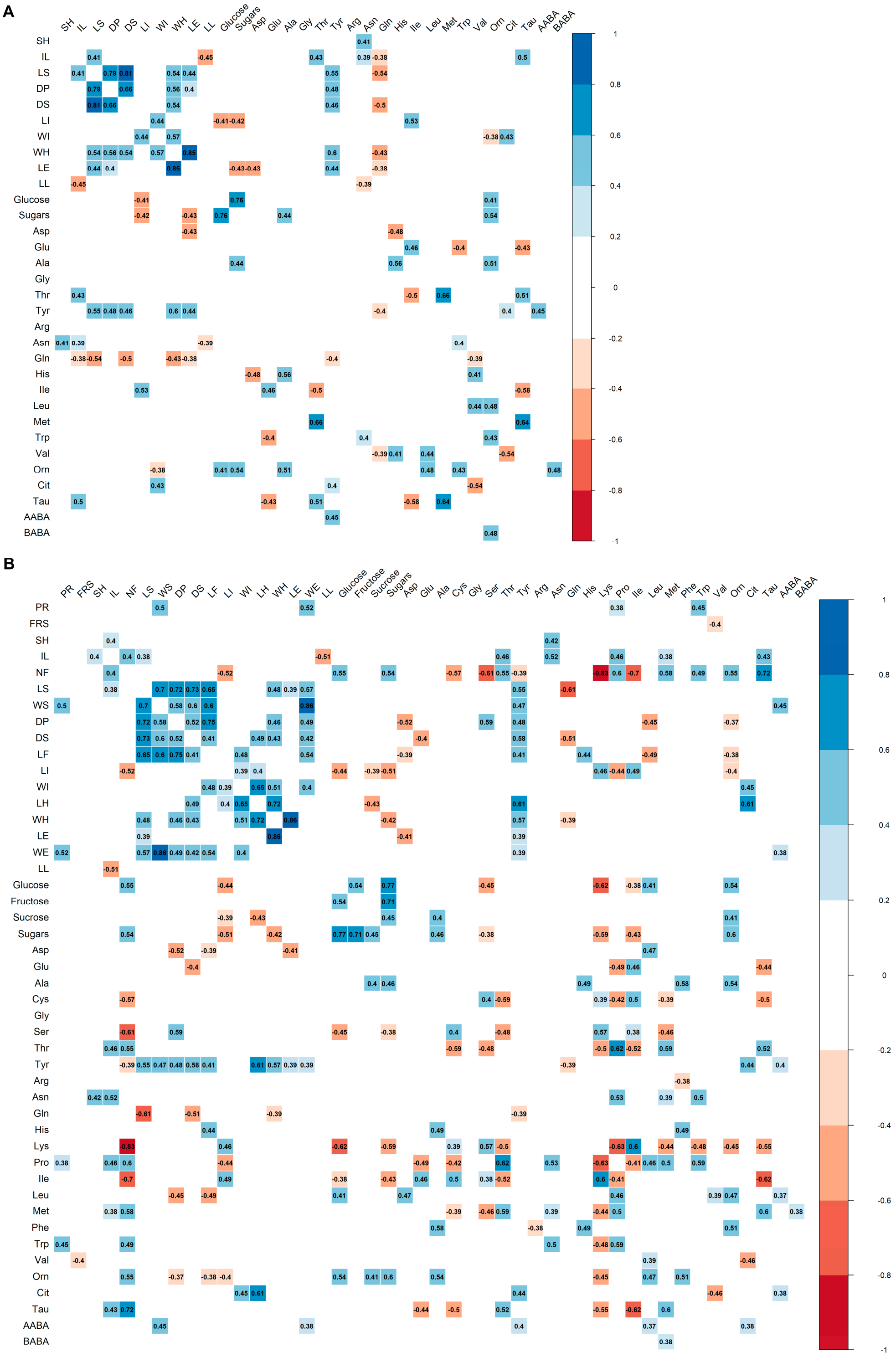

Figure S2. *Continued.*

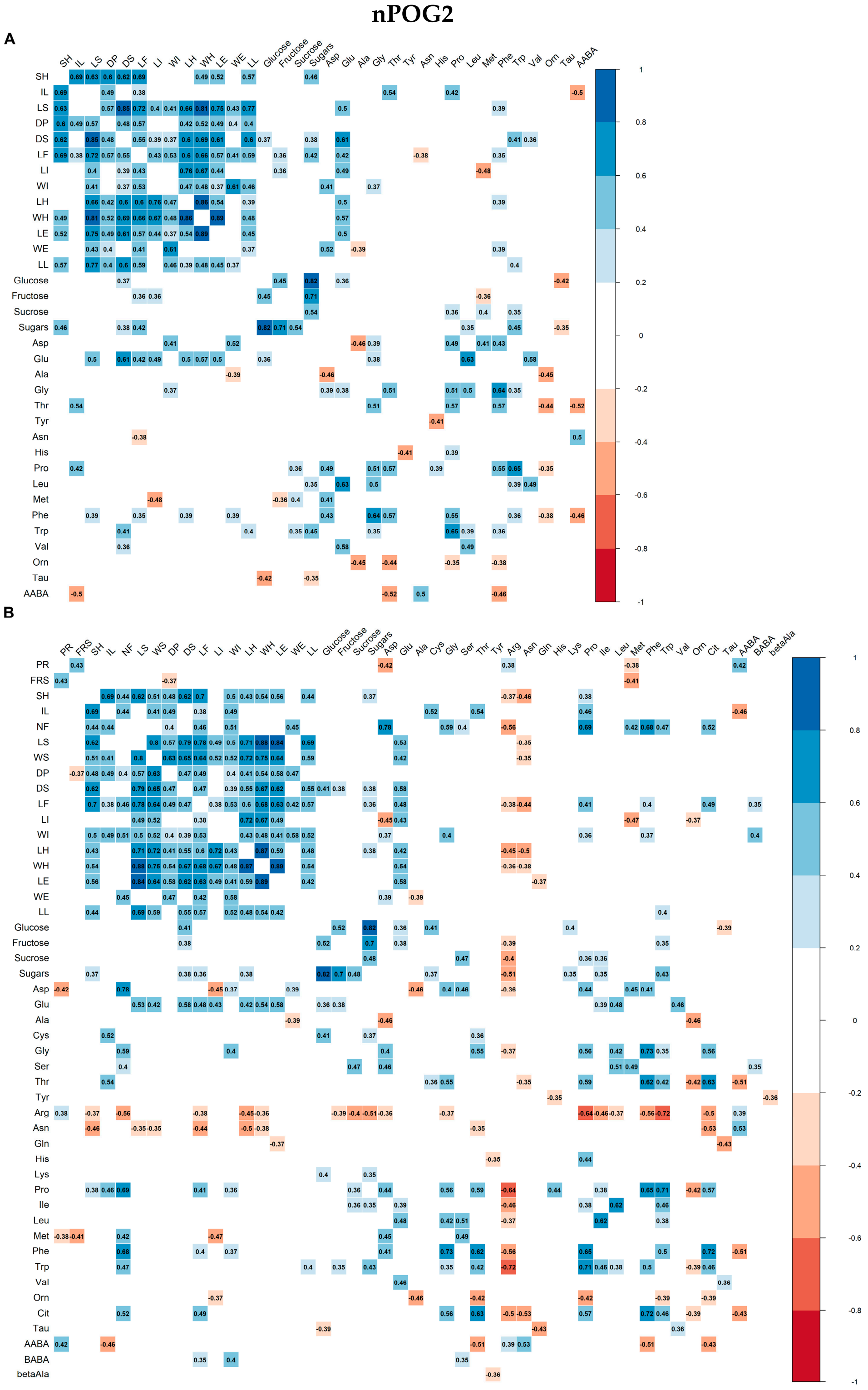

Figure S2. *Continued.*

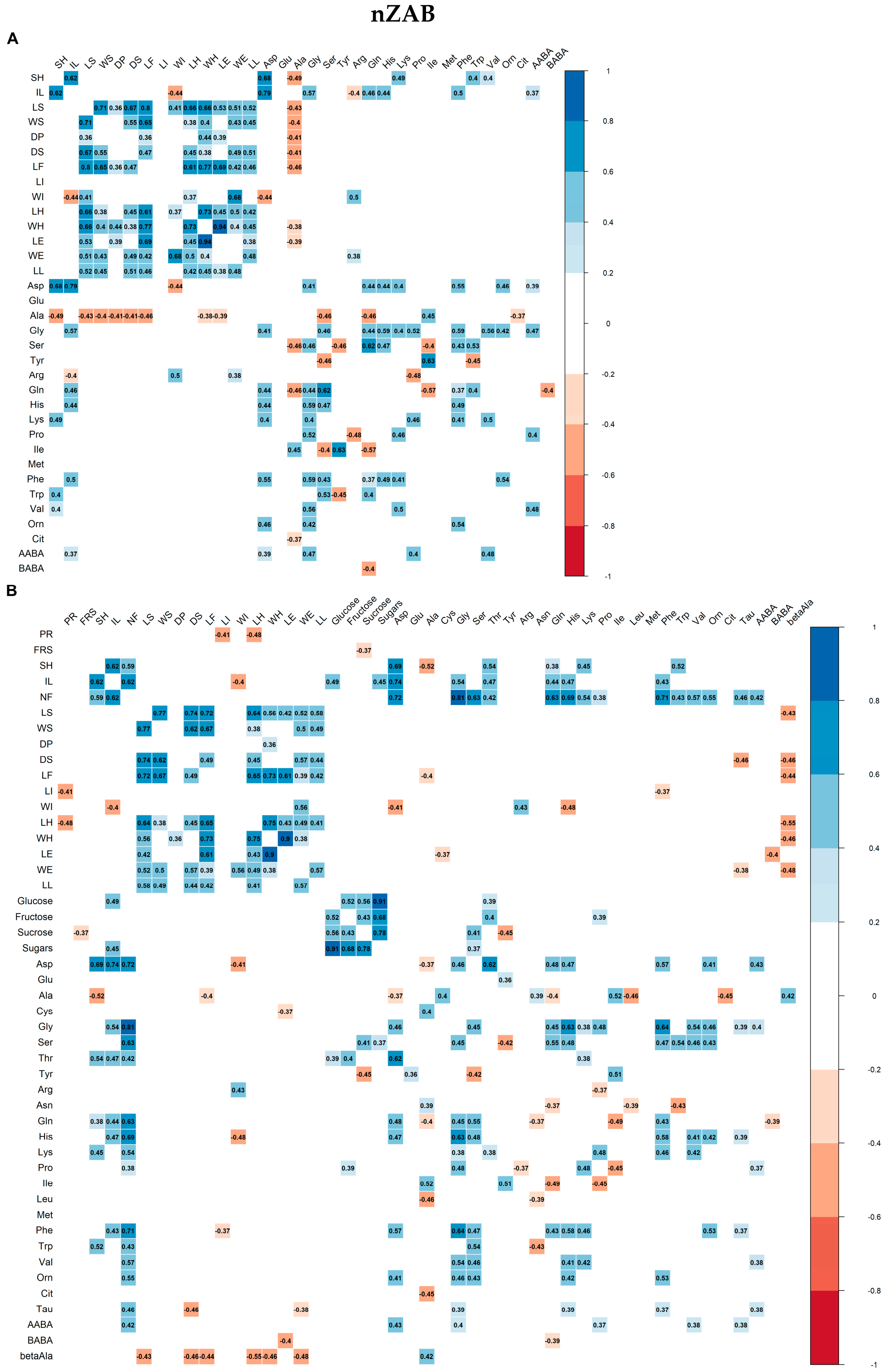

Figure S2. *Continued.*

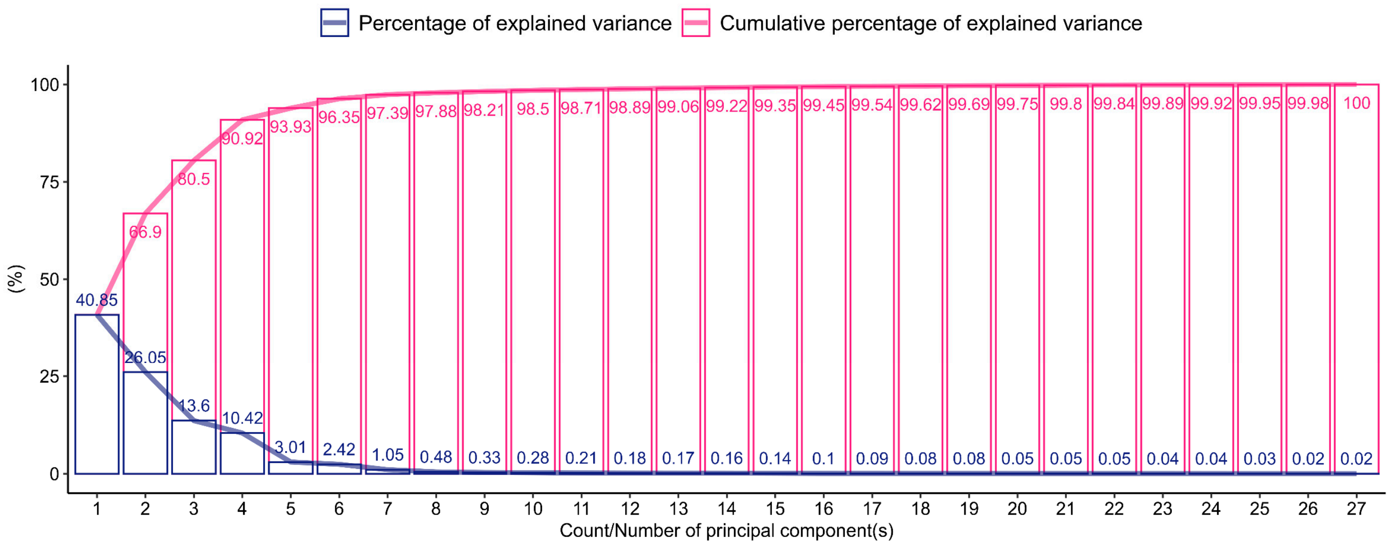

Figure S3. Scree plot showing the proportion of explained variance by the principal components of flower structure PCA model.

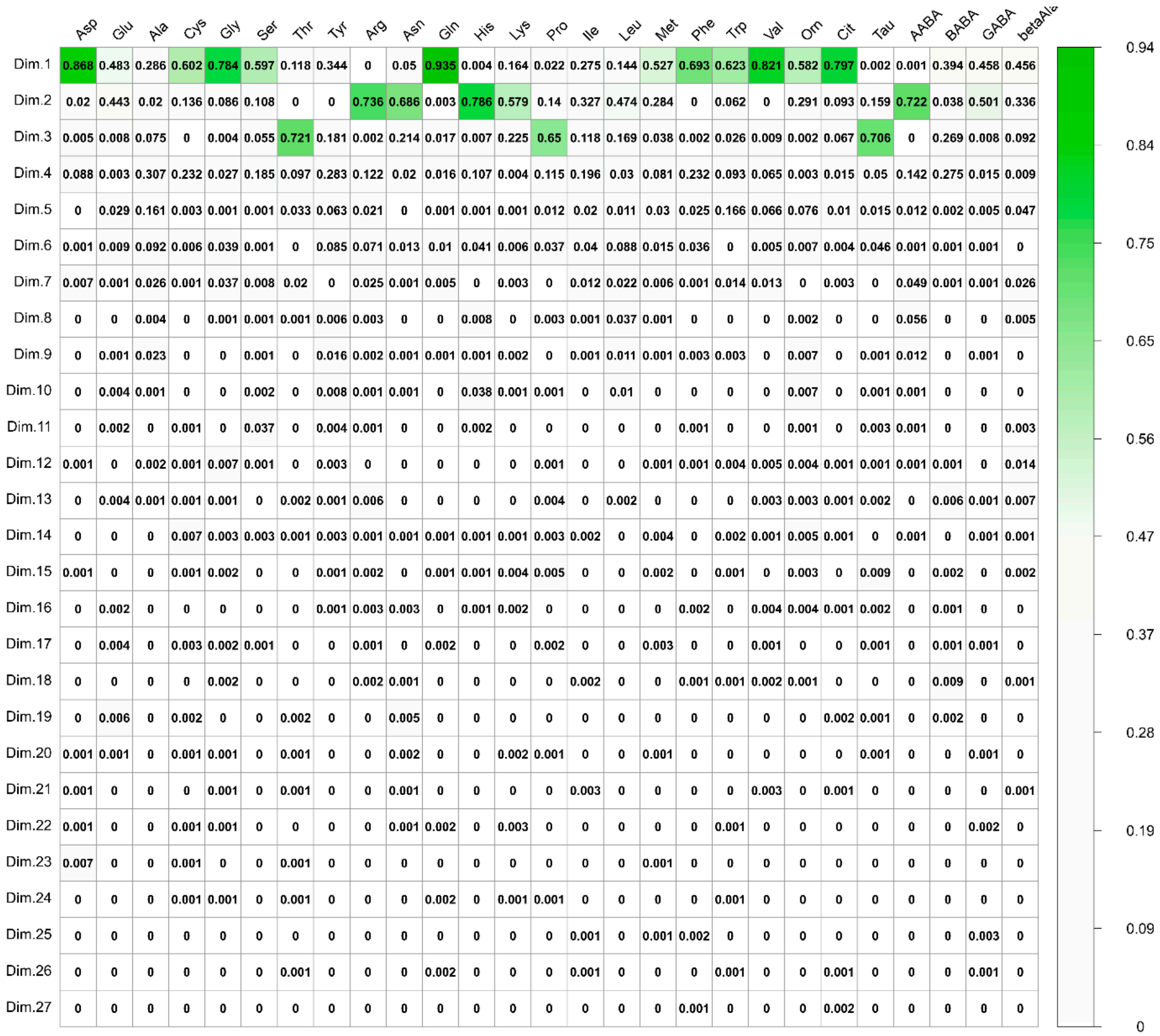

Figure S4. Cos^2 for the flower structure parameters selected as active variables in the principal component analysis model, representing the quality of representation for variables on the factor map (Dim1-16).

Table S1. Linear regression equations for |r| ≥ 0.8

**aBIA**	
LS = 4.18873711050742 + 1.40773330634875 × WS	WS = -1.56006543317214 + 0.587647539130624 × LS
LS = 2.73114979995916 + 0.447947785181096 × DS	DS = -0.326446235109149 + 1.73211624043349 × LS
LS = 2.95666365717883 + 0.700261232483824 × LF	LF = 0.646414115015877 + 1.0059461281118 × LS
WH = 3.38311751797459 + 1.13761905105747 × LE	LE = -1.42880532030627 + 0.690105141433625 × WH
**aCAR**	
WH = 3.48199772419708 + 1.15544616246949 × LE	LE = -0.408451185290991 + 0.561809505544144 × WH
**aGON**	
No correlations	
**aSOS**	
SH = 22.9306321472107 + 2.157659488013 × IL	IL = -2.44237963707565 + 0.324901227229818 × SH
LS = 3.07870593781206 + 0.460806254879927 × DS	DS = 2.02572806165663 + 1.4754661906588 × LS
LS = 3.72311640781233 + 0.693695320065387 × LF	LF = 0.156879233237415 + 1.00084733721607 × LS
WH = 4.19685191396242 + 1.01613376628778 × LE	LE = -2.17376213988751 + 0.75486645706989 × WH
**aSUR**	
SH = 20.0027544252461 + 2.15247349076929 × IL	IL = -0.875792281062978 + 0.321398653626831 × SH
LS = 2.70107314300113 + 0.673674228706618 × LF	LF = -0.105390869293299 + 1.12007504690431 × LS
LH = 0.619191115381828 + 0.406953359720849 × WH	WH = 1.88286125418483 + 1.57933604723163 × LH
WH = 2.46466757032763 + 1.34186080937916 × LE	LE = -0.602088525877769 + 0.591035576272149 × WH
**nPOG1**	
LS = 5.22634831198183 + 0.356724782183142 × DS	DS = -2.63703500161716 + 1.84499039980993 × LS
WH = 3.3387115384419 + 1.33041572139649 × LE	LE = -0.743998542899719 + 0.549141719024182 × WH
**nPOG2**	
LS = 1.74522391338346 + 0.523837868516764 × DS	DS = 2.56403556530146 + 1.38990469641038 × LS
LS = 1.99650186330575 + 1.14147626472965 × WH	WH = 1.71785896771178 + 0.57116701276861 × LS
LH = 0.372860063193264 + 0.461610189092301 × WH	WH = 1.51952075062236 + 1.60741289689113 × LH
WH = 2.22984022944677 + 1.47769064526452 × LE	LE = -0.373379466248213 + 0.538453055711908 × WH
**nZAB**	
WH = 3.46966974702284 + 1.22992216181589 × LE	LE = -1.98614123436906 + 0.715690549802021 × WH
